# Gut Microbial Topology and Metabolic Signatures Associated With Colorectal Neoplasia

**DOI:** 10.1002/advs.77991

**Published:** 2026-09-28

**Authors:** Kai Song, Youwen Qin, Jiahui Luo, Liping Liu, Chenyu Luo, Yinbin Qiu, Yiyi Zhong, Huanzi Zhong, Kui Wu, Meng Ni, Dong Wu, Min Dai, Shida Zhu, Hongda Chen

**Affiliations:** ^1^ Center for Prevention and Early Intervention National Infrastructures for Translational Medicine Institute of Clinical Medicine Peking Union Medical College Hospital Chinese Academy of Medical Sciences & Peking Union Medical College Beijing China; ^2^ Department of Gastroenterology & Department of Internal Medicine Peking Union Medical College Hospital Chinese Academy of Medical Sciences & Peking Union Medical College Beijing China; ^3^ Shenzhen Hospital Southern Medical University Shenzhen China; ^4^ BGI Genomics Shenzhen China; ^5^ Clin Lab BGI Genomics Harbin China; ^6^ Department of Cancer Epidemiology National Cancer Center/National Clinical Research Center for Cancer/Cancer Hospital Chinese Academy of Medical Sciences & Peking Union Medical College Beijing China; ^7^ BGI Precision Nutrition (Shenzhen) Technology Co., Ltd Shenzhen China

**Keywords:** colorectal neoplasia, human gut microbiome, microbiota‐derived metabolites, risk stratification, topologies

## Abstract

Alterations of gut microbial communities impact health. However, the structure and function of community‐level topologies related to colorectal neoplasia (CRN) remain unclear. We analyzed 3807 newly sequenced stool metagenomes from participants (2725 healthy controls, 759 non‐advanced adenomas, 297 advanced adenomas, and 26 colorectal cancers) in a multicenter TARGET‐C screening trial and validated our findings in multiple independent cohorts. We identified a CRN‐associated network (14 species, including *Clostridium symbiosum*) and a negatively associated network (37 species, including *Roseburia* and *Lachnospira*), forming a “seesaw‐like” microbial association pattern characterized by within‐group co‐occurrence and between‐group co‐exclusion. The structures were stable across the independent datasets. A composite score derived from the microbial topology stratified CRN risk, and diagnostic models based solely on the presence/absence status of the topological species achieved moderate accuracy across the cohorts (area under the curve ranging from 0.66 to 0.87). Functionally, changes in CRN‐related microbial association patterns were associated with microbe‐derived metabolites. Our findings provide novel insights into the microbial topology associated with CRN and support its potential application in non‐invasive risk stratification. However, the utility of these topological features in colorectal cancer remains exploratory and requires further validation in larger colorectal cancer cohorts.

## Graphical abstract

The gut microbiota organizes into two robust association patterns linked to colorectal neoplasia risk. Topological shifts in microbial networks are associated with metabolite alterations and may shape host responses during disease development.

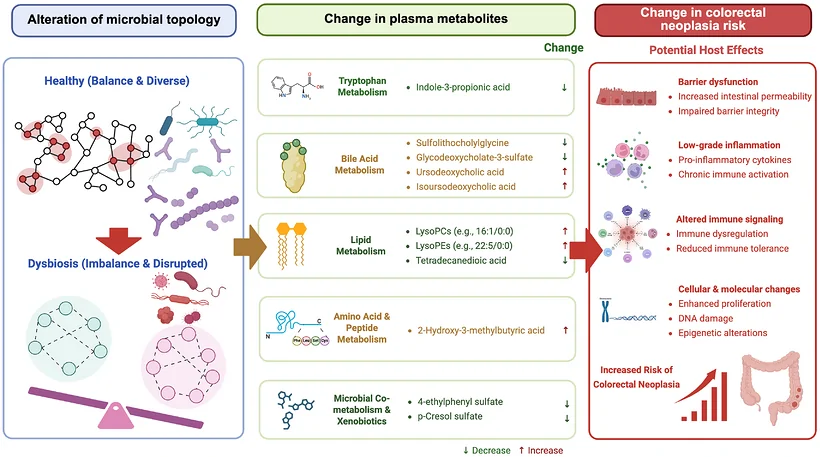

## 1 Introduction

Colorectal cancer (CRC) was the third most frequently diagnosed cancer worldwide in 2022, accounting for 9.60% of all new cancer cases. It remains a major cause of cancer‐related morbidity and mortality, imposing a substantial health and socioeconomic burden [1, 2]. The prevention and early detection of colorectal neoplasia (CRN), which includes CRC and colorectal adenoma (CRA) [3, 4, 5, 6, 7], has been shown to be effective in reducing the disease burden [8, 9, 10, 11].

Accumulating evidence has linked the development of CRN, primarily CRC, to alterations in gut microbial composition [12]. Several bacterial species, including *Fusobacterium nucleatum* subspecies *animalis*, *Bacteroides fragilis*, and *Clostridium symbiosum*, have been associated with colorectal tumorigenesis in both epidemiological and experimental studies [13, 14, 15, 16]. Clinical observations consistently show co‐abundance shifts among multiple taxa in participants with CRN [14], suggesting that microbial communities may act cooperatively or antagonistically to influence disease risk. However, most mechanistic insights from germ‐free or specific pathogen‐free mouse models have focused on a single species, thus failing to capture complex interspecies interactions [14, 17]. This complexity, confounded by interindividual variability in microbial composition, presents a major challenge for microbiome‐based diagnostics and risk stratification [17].

Recent studies have demonstrated the diagnostic potential of microbial association patterns, also known as microbial topologies, in several complex diseases. For example, analyses of microbial association patterns in diabetes and non‐small cell lung cancer have revealed distinct topological clusters predictive of disease risk or treatment response [18, 19]. Such microbial association patterns may offer more robust and generalizable biomarkers than single taxon abundance measures [18]. Notably, previous studies have observed the depletion of health‐associated species such as *Faecalibacterium prausnitzii* [20] and *Roseburia intestinalis* [21] in participants with CRN, which may imply competitive exclusion from pathogenic bacteria [14]. It remains unclear whether microbial associations in CRN are systematically organized into stable patterns. However, whether such presumed competitive relationships reflect a broader organizing principle, whereby microbial associations in CRN form stable patterns of co‐occurrence and co‐exclusion, remains unclear.

To investigate these associations, we analyzed fecal metagenomic data using a microbial network approach from a multicenter CRC screening cohort [18]. Two stable microbial association patterns were identified: a neoplasia‐associated network and a negatively associated network. These microbial association patterns were characterized by within‐group co‐occurrence and between‐group co‐exclusion, forming a seesaw‐like topological pattern. To quantify individual microbial association patterns, we developed a composite score and evaluated the utility of topological features for risk stratification across multiple external cohorts. Our findings demonstrate that microbial topologies represent a clinically relevant dimension of the gut microbiome, with potential applications in non‐invasive CRN risk stratification.

## 2 Results

### 2.1 Microbial Compositions in Participants With CRN

As shown in Figure 1, we conducted shotgun metagenomic sequencing and analyses for 3807 stool samples taken from the following participants: 2725 healthy controls (HC), 759 participants with non‐advanced adenoma (NAA), 297 participants with advanced adenoma (AA), and 26 participants with CRC (baseline characteristics are shown in Tables S1,S2). An average of 16.7 GB of high‐quality data was generated per sample (see Methods). Microbial α‐diversity based on species‐level abundance profiles, calculated using MetaPhlAn 4.0 [22], did not differ significantly between groups (analysis of covariance [ANCOVA] adjusted for covariates, *P* > 0.050; Figure S1A,B) [23]. While principal coordinate analysis revealed visual separation between the CRC group and others (Figure S1C), permutational multivariate analysis of variance (PERMANOVA) adjusted for covariates indicated that diagnostic grouping explained only 0.03% of the overall microbial variation, which was not significant (*P* = 0.153). The gut microbiomes clustered into two enterotypes, with enterotype 2 being characterized by a predominance of *Bacteroides* (Figure S1D–F). Subgroup analysis of the enterotypes indicated that disease diagnosis explained only a small proportion of the variance in the gut microbial composition (Figure S1G).

**FIGURE 1:**
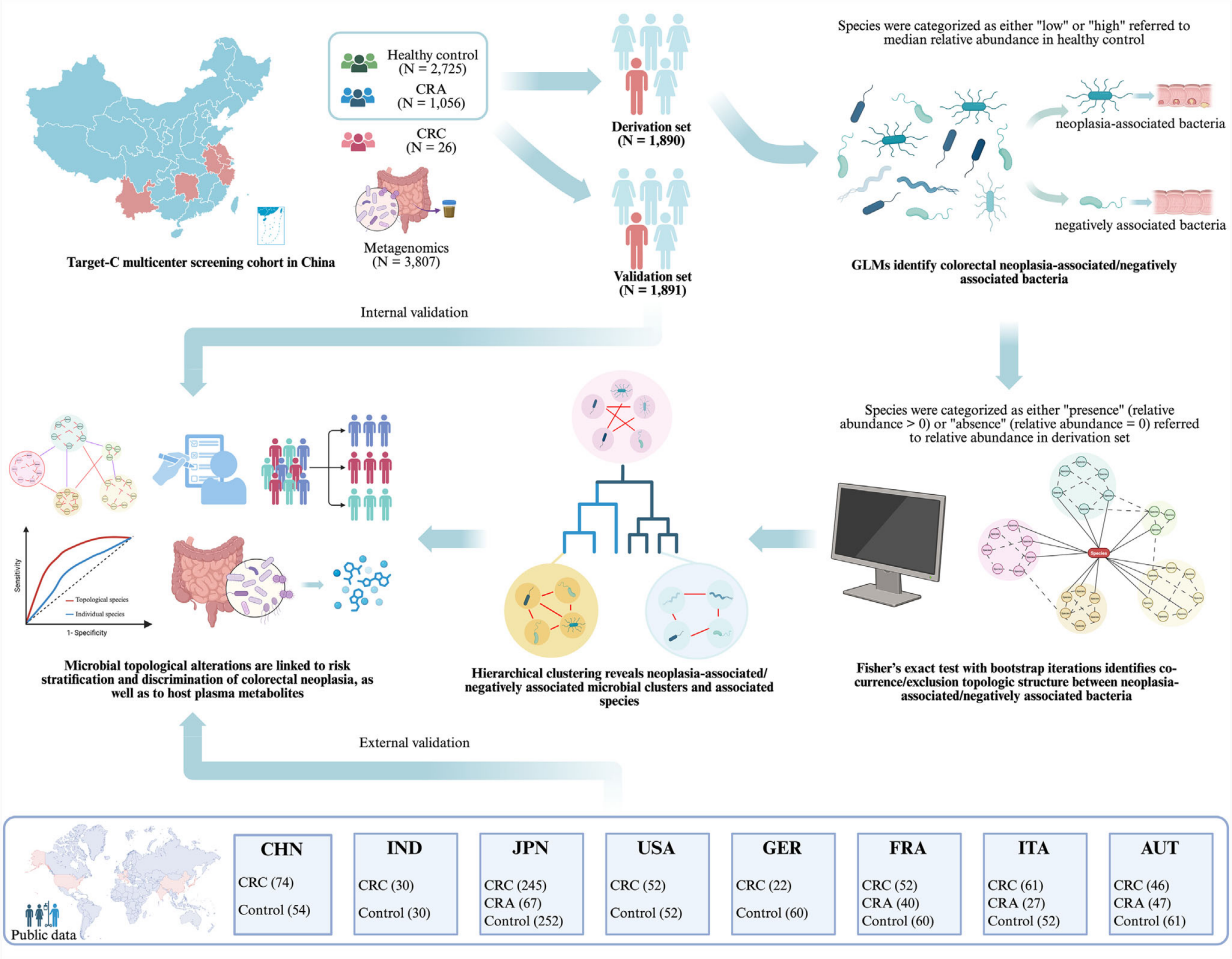
Study flowchart. CRA, colorectal adenoma; CRC, colorectal cancer; GLM, generalized linear model.

The 15 most abundant and prevalent bacterial species largely overlapped among the HC, NAA, and AA groups (Figure S2A,B), including *F. prausnitzii* (a known colorectal neoplasia‐suppressing species [20]) and *B. fragilis* (an opportunistic pathogen [24]). Linear discriminant analysis effect size (LEfSe) analysis revealed the enrichment of several pro‐tumorigenic species in NAA and AA (an absolute linear discriminant analysis [LDA] score [log10] ≥ 2, and nominal *P* < 0.050) [25, 26], such as *Fusobacterium mortiferum* [27] and *Tyzzerella nexilis* [28] in AA (Figure S2C,D). In contrast, beneficial short‐chain fatty acid (SCFA)‐producing species, including *Gemmiger formicilis* [29] in NAA and *Roseburia* spp. [30, 31] in both groups, were significantly reduced in NAA and AA content (Figure S2C,D). Several taxa showed progressive changes in abundance from HC to NAA to AA (Figure 2A). The abundances of *Phocaeicola dorei*, *Faecalibacterium SGB15346*, and *F. prausnitzii* were reduced, suggesting a stepwise change in the development of CRN. ANCOM‐BC2 [32] (Analysis of Compositions of Microbiomes with Bias Correction 2) further confirmed these gradual abundance changes after false discovery rate (FDR) correction (FDR < 0.050): *F. prausnitzii* (NAA vs. HC, Log FC [fold change] = −0.16; AA vs. HC, Log FC = −0.36), *F. SGB15346* (NAA vs. HC, Log FC = −0.04; AA vs. HC, Log FC = −0.30), and *P. dorei* (NAA vs. HC, Log FC = −0.40; AA vs. HC, Log FC = −0.73) (Figure 2B; Table S3; and Figure S2 for pairwise comparisons). These results support the consistent role of these key microbes in the adenoma‐carcinoma sequence.

**FIGURE 2:**
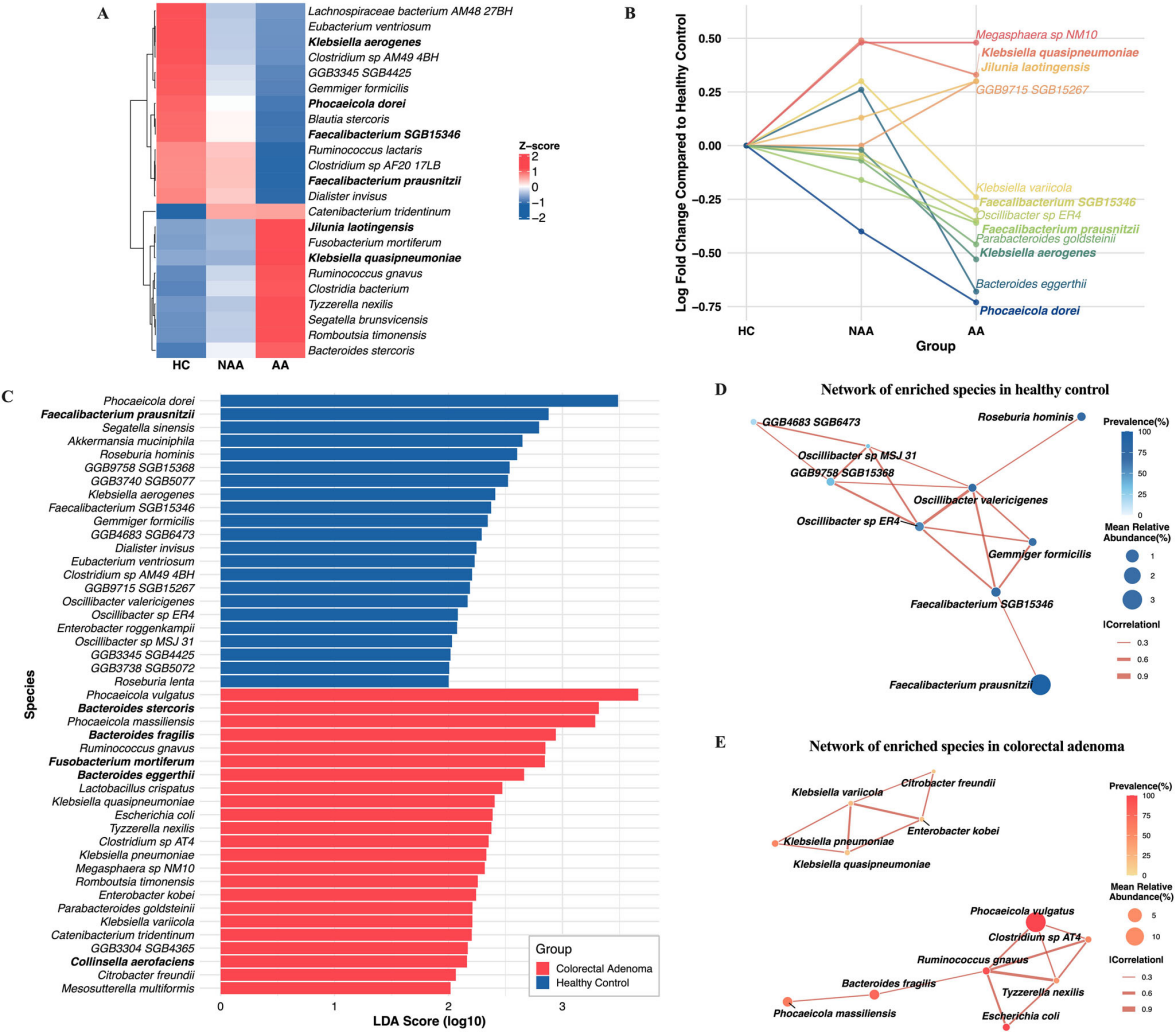
Taxonomic features of gut microbiome in CRA. (A) Heatmap of differential species between the NAA and AA groups versus healthy controls as identified by LEfSe (*LDA* score > 2, *P* < 0.050). Species identified as significant by ANCOM‐BC2 are highlighted in bold. (B) Trend analysis of differential taxa across HC, NAA, and AA using ANCOM‐BC2; species overlapping with panel (A) are in bold. (C) Differential species between CRA and HC identified by LEfSe; previously reported neoplasia‐associated taxa are shown in bold. The co‐abundance network of enriched species in (D) HC and (E) CRA, calculated using SparCC. Significant co‐abundance is displayed with a correlation coefficient >0.30 and false discovery rate (FDR) < 0.050 using 100× permutation. Blue nodes indicate species enriched in HC, whereas red nodes represent species enriched in CRA. Node size represents mean relative abundance, whereas node color intensity reflects prevalence. The linewidth indicates the correlation strength (absolute value).

### 2.2 The Dilemma of Microbial Signatures in CRN

Because LEfSe analysis identified no species that differed significantly between the AA and NAA groups, we combined these participants into the CRA group. Several species differed in abundance between the CRA and HC groups. Known CRC‐associated pathogenic species were enriched, including *B. fragilis* [14], *Collinsella aerofaciens* [33], *Bacteroides stercoris* [34], *Bacteroides eggerthii* [34], and *F. mortiferum* [27], while beneficial species such as *F. prausnitzii* [20] and *Roseburia* spp. [30] were depleted (Figure 2C). Co‐abundance correlation networks constructed with Sparse Correlations for Compositional data (SparCC) [35] (|correlation| > 0.30, FDR < 0.050) suggested that altered association patterns may contribute to CRA development (Figure 2D,E). Using multivariable association with linear model 2 (MaAsLin2) [36], we identified species that were significantly associated with CRA or CRC (FDR < 0.050; Figure 3A,B). However, the specific species associated with CRA or CRC, particularly CRA, varied considerably across cohorts analyzed using the same method.

**FIGURE 3:**
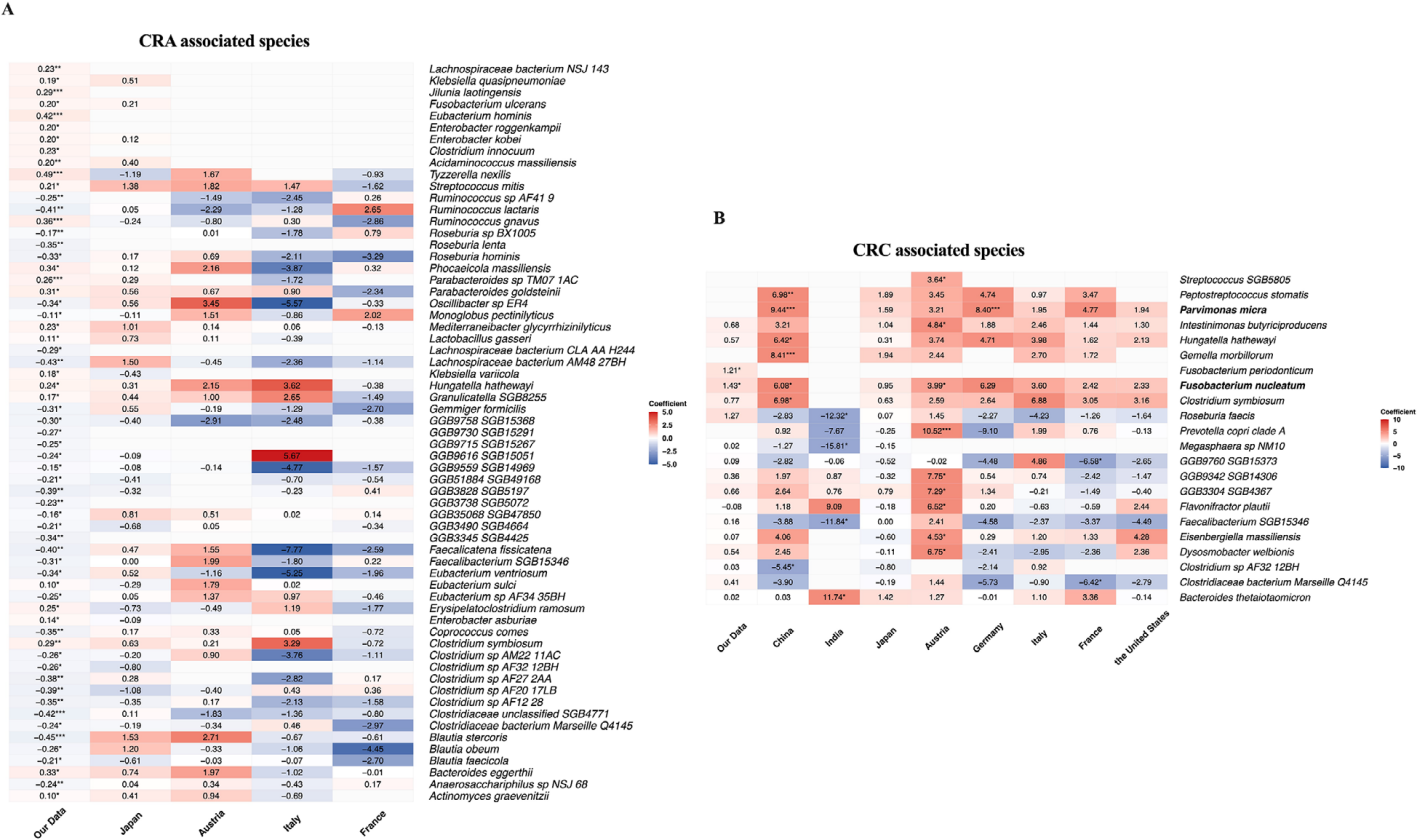
Associations between gut bacterial species and CRN. Associations between gut bacterial species with (A) CRA and (B) CRC across our cohort and external datasets, assessed using MaAsLin2 (adjusted for sex, age, and BMI). Red and blue squares denote positive and negative associations, respectively, with the corresponding color intensity and numerical values indicating the coefficient magnitude. Species significant (FDR < 0.050) in at least two datasets are shown in bold. Asterisks indicate FDR thresholds (*** < 0.001, ** < 0.010, and * < 0.050). To correct for an imbalance between colorectal cancer cases and controls, MaAsLin2 was conducted after 1:3 nearest‐neighbor matching by age, sex, and BMI.

These results highlight a dilemma: despite the identification of differentially abundant species, their inconsistent performance across cohorts limits their robustness as CRA biomarkers. This inconsistency motivated us to investigate higher‐order microbial community structures that may exhibit greater conservation.

### 2.3 Altered Microbial Topology Underlying CRA

Given the limited explanatory power of individual species abundance, and inspired by the Ren et al. study [37], which showed that the topology of the gut microbiota is associated with clinical outcomes, we hypothesized that community‐level microbial association patterns may underlie CRA development. We examined the changes in the microbial association patterns identified by SparCC [35] (|correlation| > 0.30, FDR < 0.050; Figure S3A,B). The patterns in participants with CRA consisted of 353 species and 2594 association pairs, whereas the HC network was larger yet less dense, comprising 416 species and 3011 association pairs. Positive associations predominated in both networks (75% in CRA, 73.20% in HC). A comparison of the topology metrics between the HC and CRA groups revealed differences (Table S4), indicating a fundamental reorganization of the microbial association patterns. To identify the taxa driving this reorganization, we examined the variations in the composition of topologically central species using a composite score derived from the weighted sum of the degree, betweenness, closeness, and eigenvector centralities (Figures S3A,B,S4A,B and Table S5). This analysis showed that during the transition from HC to CRA, the centrality of health‐associated taxa from the *Oscillospiraceae* family [38] declined, whereas neoplasia‐associated species, including *Erysipelatoclostridium ramosum*, *Ruminococcus gnavus*, and *Faecalimonas umbilicata*, gained prominence in centrality. Within‐module (Zi) and among‐module (Pi) connectivity further indicated that protective bacteria lost network connections (Figure S4C,D) [39]. Intriguingly, some taxa, such as *Clostridium paraputrificum* [40], maintained central positions within the topological organization of the microbial association network underlying CRA. In addition, certain species, such as *E. ramosum*, occupied central positions within the microbial association network despite not being significantly enriched in abundance in the CRA group, suggesting that network analysis can capture information that abundance‐based comparisons miss.

To further identify and quantify CRA‐related microbial association patterns, we constructed association networks based on species co‐occurrence and co‐exclusion, as proposed by Derosa et al. [18], rather than relative abundance correlations. A comparison between the derivation and validation sets is presented in Table S6. From 22 895 initial association pairs, we identified 927 robust co‐occurrence/co‐exclusion associations involving 77 CRA‐related species (odds ratio [OR] ≥ 1.10 or OR ≤ 0.80 in generalized linear models [GLM]; see Methods for details), which were retained for downstream network analyses (Table S7). Hierarchical clustering of these associations revealed 12 clusters (C1–C12). Three of the clusters (C9, C10, and C12) contained 14 species (T1 species) that were all positively associated with CRA (GLM OR ≥ 1.10), thus defining a neoplasia‐associated network (T1 topology). Four of the clusters (C4, C6, C7, and C8) contained 37 species (T2 species), most (94.59%) negatively associated with CRA (GLM OR < 0.80), thus defining a negatively associated network (T2 topology) (Figure 4; Table S8). Almost half of the T1 species (6/14) had a prevalence of less than 20% in participants with CRA, whereas 59% of the T2 species had a prevalence greater than 30%. The T1 topology encompassed several well‐known species associated with CRC risk, including *C. symbiosum* and *Hungatella hathewayi* [41, 42], underscoring their potential role as early drivers of colorectal tumorigenesis. In contrast, the T2 topology was predominantly composed of SCFA‐producing or health‐associated species with documented protective effects. These species included members of the genera *Lachnospira and Roseburia*, which have previously been associated with maintaining intestinal health and suppressing neoplastic development [43, 44, 45].

**FIGURE 4:**
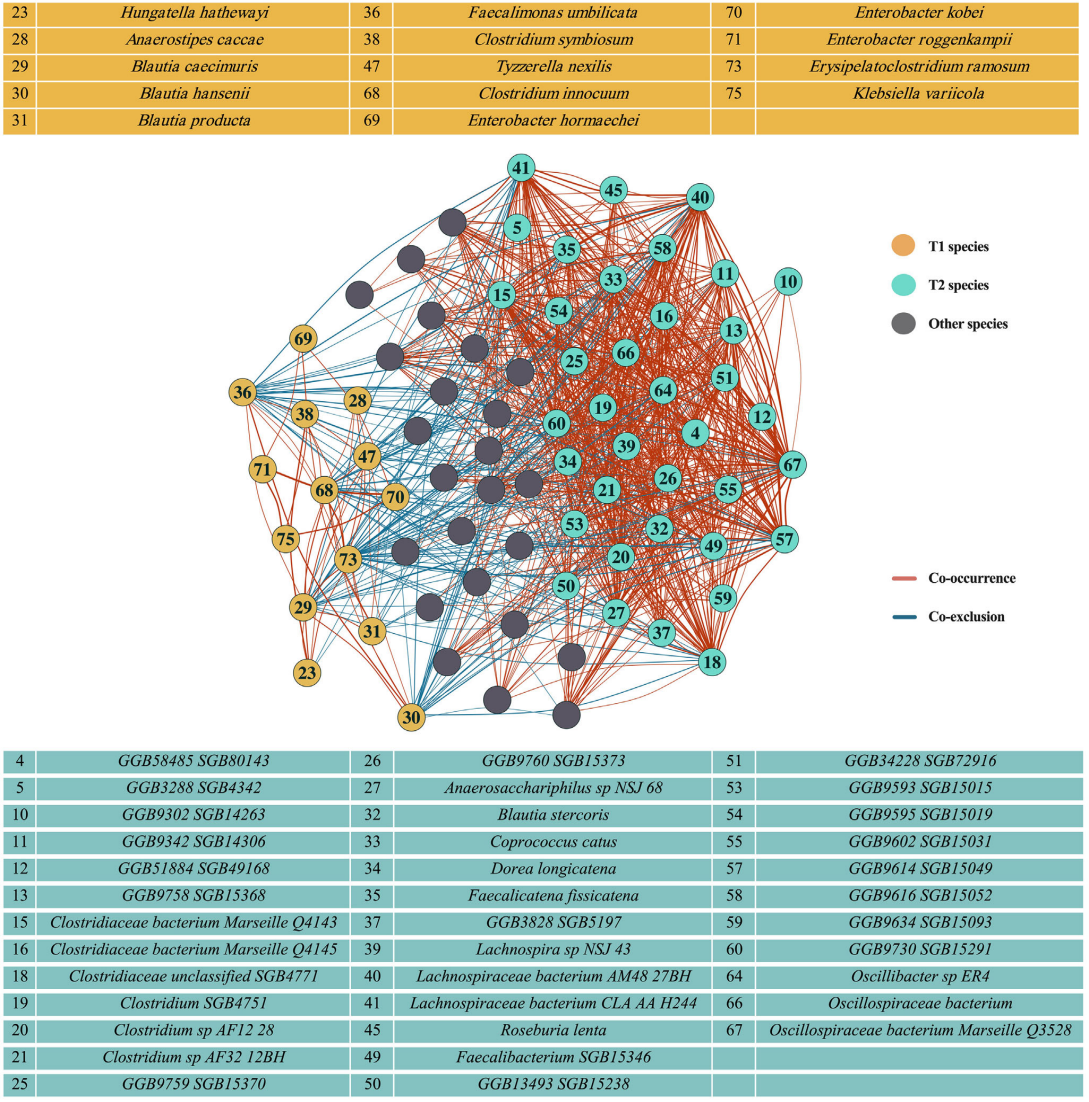
Reciprocal topological organization of CRA‐related microbial association patterns. Microbial association patterns of 77 species in the derivation set. Edges represent significant pairwise associations identified using Fisher's exact test with Bonferroni correction (adjusted *P* < 0.050). Red and blue lines indicate co‐occurrence and co‐exclusion, respectively. Yellow and green nodes represent species belonging to the neoplasia‐associated network (T1 topology) and the negatively associated network (T2 topology), respectively, whereas gray nodes indicate species not included in the neoplasia‐related microbial association patterns.

Microbial association patterns between species from the T1 and T2 topologies were exclusively negative, whereas patterns within each topological group were consistently positive, indicating significant topological separation between the two networks (Figure 4).

### 2.4 Validation of CRA‐Related Microbial Topologies

To validate these CRA‐related microbial association patterns, we examined an internal cohort comprising 533 patients with CRA and 1358 HC. The topological patterns were consistent with those identified in the derivation set (Figure S5): within the T1 and T2 species groups, all of the associations represented co‐occurrence relationships, whereas all of the associations between T1 and T2 represented co‐exclusion relationships. Notably, the identified association patterns were robust to different OR thresholds: OR ≥ 1.10/≤0.90 (Figure S6), OR ≥ 1.05/≤0.95 (Figure S7), and OR > 1/<1 (Figure S8).

Furthermore, we validated these microbial association patterns in participants with CRA or CRC across eight external cohorts from China, India, Japan, the United States, Germany, France, Italy, and Austria (participant details are provided in Table S9). In this integrated external CRN cohort (181 CRA and 582 CRC), 39 topological species were detected, representing 76.47% of all topological species (Table S10). Conserved topological patterns were observed in participants with CRA and in those with CRC (Bonferroni‐adjusted *P* < 0.050; Figure S9A,B), characterized by within‐group co‐occurrence (T1 and T2) and between‐group co‐exclusion (T1 vs. T2). Most topological species maintained their association patterns in the integrated external cohort, and all topological species were consistently categorized into T1 or T2 groups (Figure S9A,B). We further validated the microbial association patterns identified above (Figure S9C) in an independent external cohort comprising 460 participants with CRC from the Guangzhou Center.

Taken together, the microbial association patterns identified in participants with CRA were also observed in external CRC cohorts, suggesting that CRA‐associated microbial dysbiosis may be involved in the adenoma–carcinoma sequence. The reproducibility of these microbial association patterns across geographically diverse cohorts supports the potential for CRN risk stratification.

### 2.5 Microbial Topological Alterations Associated With Risk of CRA and CRC

To quantify alterations in microbial association patterns, we followed the approach of Derosa et al. [18] and constructed a unidimensional topology score (T‐score) to characterize each individual's microbial association patterns. We observed significantly elevated T‐scores in participants with CRA compared with HCs in both the derivation and validation sets (Figure 5A–D). Multivariable analysis confirmed that the T‐score was an independent risk factor for CRA. In the derivation set, each 0.01‐unit increase in the T‐score was associated with a 1% increase in the odds of CRA (OR 1.01, 95% CI: 1.01–1.02; Table 1). This unit increase corresponded to the smallest change induced by adding one T1 species or removing one T2 species from an individual network. The CRA risk increased across T‐score quartiles in both the derivation (Q4 vs. Q1: OR, 1.70; Q3 vs. Q1: OR, 1.26) and validation sets (Figure 5E,F). After integrating data from participants with CRC at the Guangzhou Center, T‐scores differed significantly among the HC, CRA, and CRC groups (Kruskal–Wallis test, *P* < 0.001). Post‐hoc pairwise comparisons using Dunn's test with Bonferroni correction showed higher T‐scores in participants with CRC than in the HC and CRA groups (Figure 5G). This stepwise increase in T‐scores across the HC, CRA, and CRC groups was confirmed using the Jonckheere–Terpstra test (*P* = 0.001). Multivariable analysis (adjusted for age, sex, and BMI, the only covariates consistently available for the Guangzhou cohort) confirmed that T‐score was an independent risk factor for CRC (OR 1.02, 95% CI: 1.01–1.02).

**FIGURE 5:**
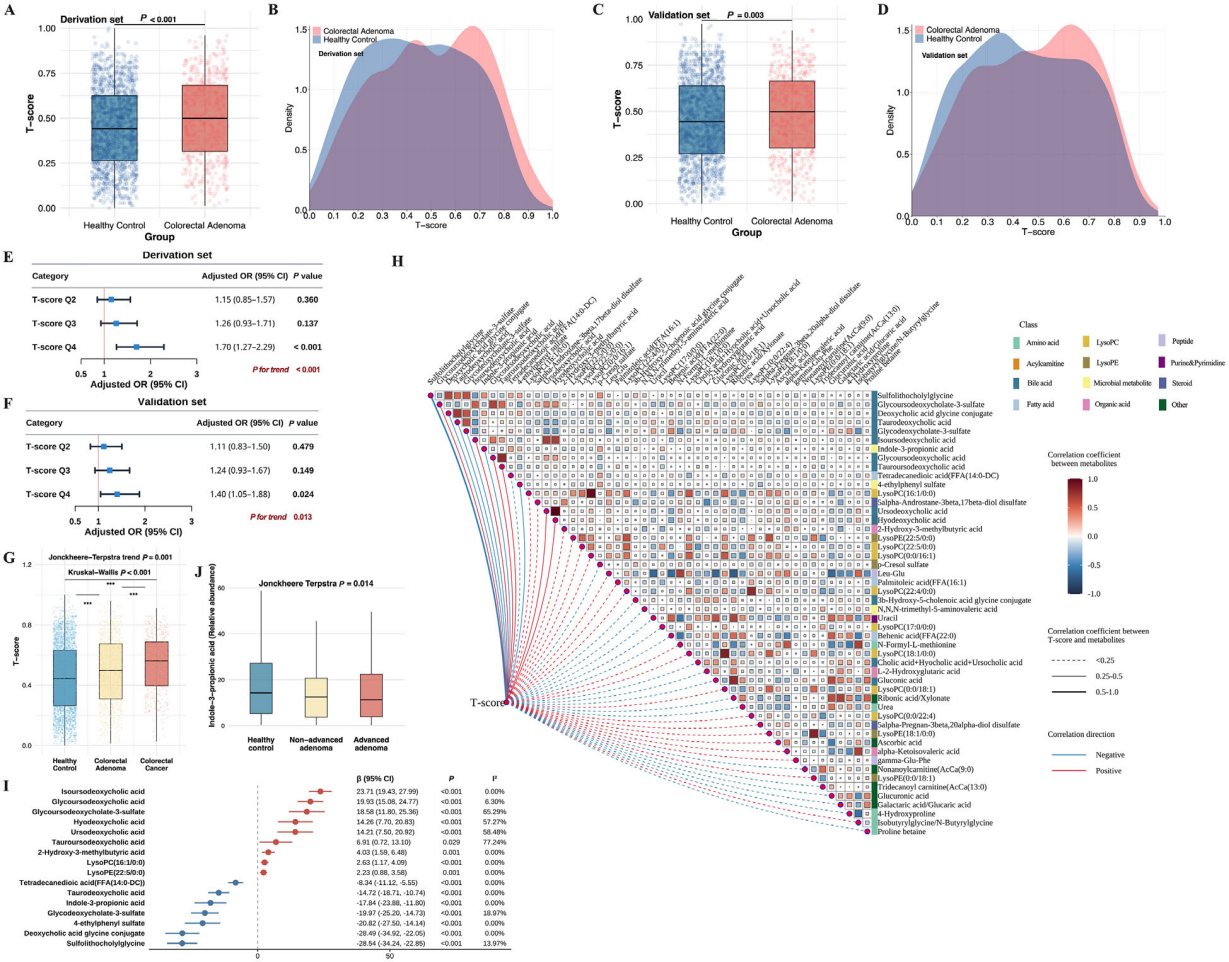
Comparison of topology scores and the associations with CRA and plasma metabolites. Topology score (T‐score) distributions in the (A) derivation and (C) validation sets. Kernel density estimation (KDE) of T‐scores comparing participants with CRA and HCs in the (B) derivation and (D) validation sets. CRA risk was stratified by T‐score quartiles in the (E) derivation and (F) validation sets, with the first quartile as the reference. (G) T‐score comparison between CRC participants from a collaborating center and participants with CRA and HCs from the internal cohort. The results of the Kruskal–Wallis test for intergroup comparisons and the Jonckheere–Terpstra test for trend analyses are shown. *** indicates *P* < 0.001; ** indicates *P* < 0.010; * indicates *P* < 0.050. (H) Spearman correlation analysis between T‐scores and plasma metabolites as well as inter‐metabolite correlations. Significant correlations (false discovery rate < 0.050) were observed. Red and blue lines indicate positive and negative correlations between T‐scores and metabolites, respectively, with line thickness and type reflecting the correlation strength. Squares represent inter‐metabolite correlations, with color and size denoting direction and magnitude, respectively. (I) Pooled associations between T‐scores and plasma metabolites across internal validation centers. Forest plot showing the pooled effect estimates (*β*) and 95% CIs for the plasma metabolites significantly associated with the T‐score (pooled *P* < 0.050). Red and blue indicate positive and negative associations with the T‐score, respectively. (J) Jonckheere–Terpstra trend test of plasma IPA levels across healthy controls, participants with NAA, and participants with AA; horizontal lines above boxes indicate the third quartile plus 1.5 × interquartile range. CI, confidence interval; OR, odds ratio; Q2, second quartile; Q3, third quartile; Q4, fourth quartile.

**TABLE 1:** Association between topology score and CRN risk stratification.

Dataset	Type of CRN	OR (95% CI)^a^, ^b^	*P*‐value^a^
Derivation set	Colorectal adenoma	1.01 (1.01–1.02)	<0.001
Validation set	Colorectal adenoma	1.01 (1–1.01)	0.005
Austria	Colorectal adenoma	1.00 (0.96–1.04)	0.958
Italy	Colorectal adenoma	1.04 (0.98–1.11)	0.221
France	Colorectal adenoma	1.01 (0.96–1.06)	0.679
Japan	Colorectal adenoma	1.00 (0.98–1.02)	0.940
China	Colorectal cancer	1.05 (1.02–1.08)	0.002
India	Colorectal cancer	1.39 (1.13–1.87)	0.010
United States	Colorectal cancer	1.05 (1.02–1.09)	0.004
Germany	Colorectal cancer	1.10 (1.04–1.17)	0.001

^a^ In our cohort, the association was adjusted for age, sex, body mass index, recruitment region, family history of colorectal cancer in first‐degree relatives, smoking status, drinking status, diabetes, hypertension, dietary pattern, and regular use of antibiotics and probiotics. In the external datasets, the association was adjusted for age, sex, body mass index.

^b^ Change in colorectal neoplasia risk per 0.01‐unit increase in the T‐score (corresponding to the minimal increase induced by the addition of one T1 species or removal of one T2 species from the microbial association pattern).

CI: confidence interval; CRN: colorectal neoplasia; OR: odds ratio.

Although the T1/T2 groups were validated externally, the T‐score itself did not show a significant difference between participants with CRA and HC in the four independent adenoma cohorts (Table 1). Nevertheless, the T‐score demonstrated discriminatory ability for CRC risk stratification across multiple external CRC cohorts (Table 1; Figure S10A–H). Across a diverse range of cohorts from China, the United States, Germany, and India, T‐scores were significantly higher in participants with CRC than in HCs. Moreover, after adjusting for age, sex, and BMI, the T‐score remained associated with an increased CRC risk. Specifically, for every 0.01‐unit increase in the T‐score, the risk of CRC increased by 5%–39% (Table 1).

### 2.6 Associations Between CRA‐Related Microbial Association Patterns and Plasma Metabolite Profiles

Given the link between the T‐score, CRA, and CRC risk, plasma metabolomic data from 806 participants (255 NAA, 279 AA, and 272 HC) were utilized to investigate associations between the T‐score and metabolite profiles. In the derivation set, Spearman's correlation analysis revealed significant associations between the T‐score and multiple metabolites (FDR < 0.050), including bile acids (e.g., isoursodeoxycholic acid) and microbe‐derived metabolites (e.g., indole‐3‐propionic acid [IPA] and 4‐ethylphenyl sulfate) (Figure 5H; Table S11). For instance, the T‐score was positively correlated with isoursodeoxycholic acid (*r* = 0.50, FDR < 0.001), a metabolite linked to postprandial inflammation [46], suggesting that alterations in the microbial topology could influence bile acid metabolism to promote tumorigenesis.

Among the microbe‐derived metabolites, IPA exhibited the strongest inverse association with the T‐score (*r* = −0.31, FDR < 0.001), followed by 4‐ethylphenyl sulfate (*r* = −0.28, FDR < 0.001). IPA has been linked to multiple health benefits, while 4‐ethylphenyl sulfate has been reported to alleviate colonic inflammation in animal models [47, 48, 49]. In the validation, center‐specific multivariable linear regression analyses followed by random‐effects meta‐analysis showed that 16 plasma metabolites remained significantly associated with the T‐score (pooled *P* < 0.050; Figure 5I; Table S12).

Notably, plasma IPA levels were significantly lower in participants with CRA than in HCs (Wilcoxon rank‐sum test, *P* = 0.021), and the Jonckheere–Terpstra trend analysis showed that IPA decreased progressively from HCs to NAA to AA (*P* = 0.014; Figure 5J). When stratified by the median plasma IPA level in HCs, participants with low IPA had a significantly higher risk of CRA than those with high IPA (adjusted OR 1.45, 95% CI: 1.04–2.14).

Collectively, these findings suggest that CRA‐related microbial association patterns may be associated with tumorigenesis through alterations in microbial‐derived metabolites and host bile acid metabolism.

### 2.7 Discrimination Performance of CRC Based on Microbial Topological Features

Given the encouraging performance of CRA‐related microbial association patterns in CRC risk stratification in public datasets, we explored whether topology‐derived features could discriminate CRC using these datasets. Random forest modelling was performed using eight public datasets. For each dataset, using a leave‐one‐out cross‐validation scheme, the predictors were either the presence/absence status or relative abundance of the topological species. Across external cohorts from China, Germany, India, Austria, Italy, and France, models based on presence/absence status achieved areas under the receiver operating characteristic curves (AUCs) of 0.66–0.87 (Figure 6A; Figure S11A,B), while those based on relative abundance achieved AUCs of 0.61–0.87 (Figure 6B; Figure S11C,D). The model showed satisfactory calibration in most cohorts (Spiegelhalter's z‐test, *P* > 0.050; Table S13). For the Austrian and German datasets, satisfactory calibration was achieved after recalibration using Platt scaling. The results of the decision curve analysis are shown in Figure S12. Some species, including *C. symbiosum*, *H. hathewayi*, *Faecalicatena fissicatena*, *Lachnospira sp. NSJ 43*, and *Oscillibacter sp. ER4*, consistently ranked high in variable importance across most of the datasets (Figure 6C,D). Several species exhibiting consistent CRC‐associated trends across cohorts also remained influential in the models built on topological species, such as *C. symbiosum* and *H. hathewayi* (Figure 3B). In addition, *E. ramosum*, which was identified as a topologically central node in the SparCC co‐abundance network (Figure S4B), continued to show prominent importance in models based on topological species (Figure 6C,D). The inclusion of clinical variables (age, BMI, and sex) further improved the discriminative performance of the model (Figure S13). The consistency of these findings supports the reproducibility of the identified topological species across external cohorts. Nevertheless, the application of topology‐derived features for CRC discrimination should be considered exploratory given the limited number of CRC cases in our derivation set, and warrants further validation in larger CRC cohorts.

**FIGURE 6:**
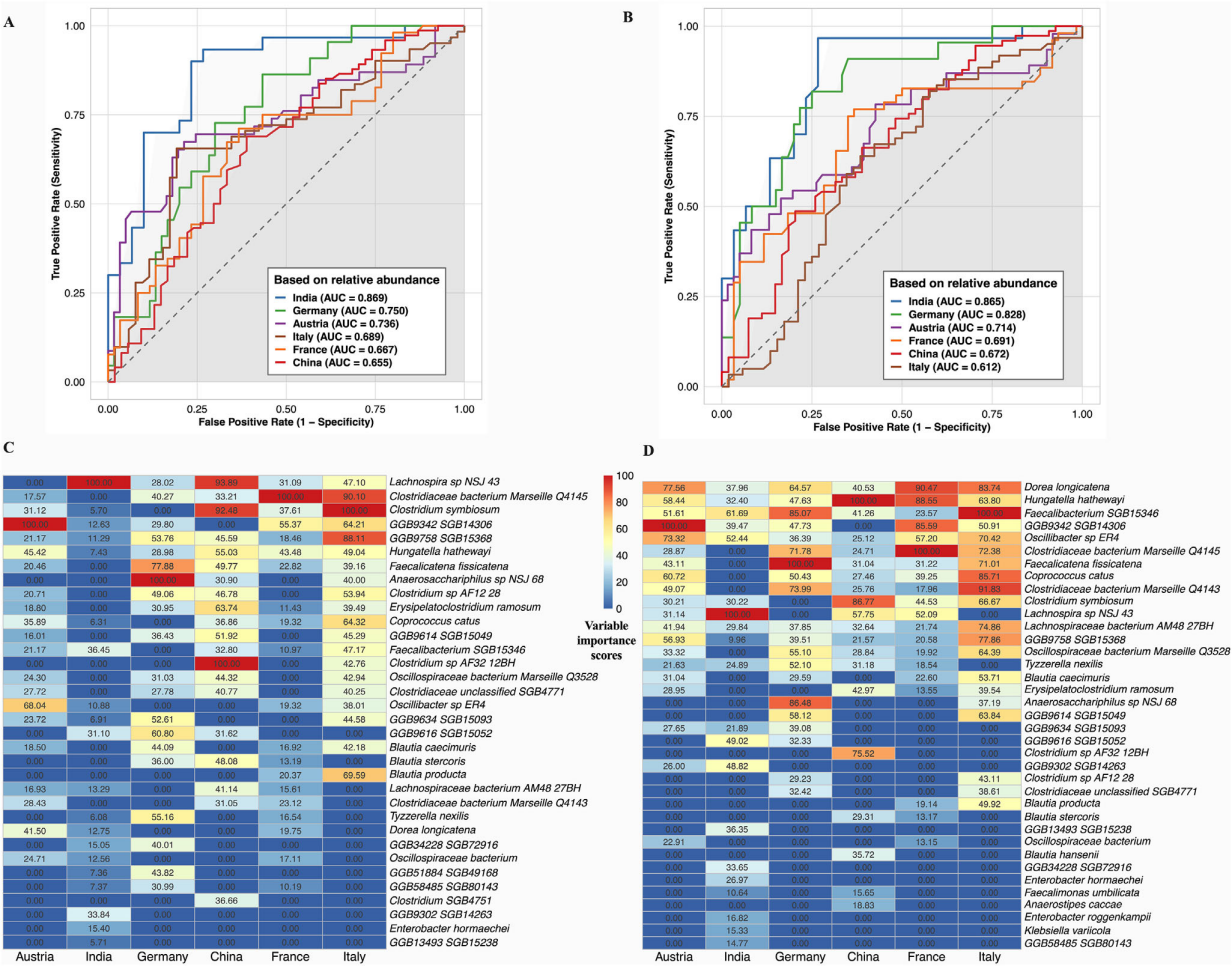
Performance of the discriminatory model based on topological species for CRC. (A) Performance of the random forest models based on the presence or absence of topological species (leave‐one‐out cross‐validation [LOOCV], 1000 trees) in datasets from India, Germany, Austria, Italy, France, and China. (B) Models based on relative abundance of species (LOOCV, 1000 trees) in the same datasets. (C) Top 20 species ranked by variable importance scores in random forest models based on species presence/absence in Austria, India, Germany, China, France, and Italy. (D) Top 20 species in the models based on the relative abundance of topology‐associated species. The numbers inside the boxes represent the variable importance scores, with zero indicating that the species was not among the top 20 in that dataset. The color gradient from blue to red reflects increasing importance scores. AUC, area under the receiver operating characteristic curve.

Models based solely on topological species exhibited unsatisfactory discriminative power for CRC detection. Among all species consistently associated with CRC across at least three cohorts, *F. nucleatum*, *Parvimonas micra*, and *Gemella morbillorum* exhibited the strongest associations [50, 51]. To improve performance, we identified 208 conserved association pairs (involving 44 topological species) (Table S14) and developed a novel microbial association strength (MAS) metric. For each individual, MAS was calculated as the sum of the β‐coefficients of all conserved association pairs present in that individual, with the sign inverted for T2 pairs (see Methods). We incorporated the MAS metric into prediction models based on these species. This consistently improved the discriminatory performance across most external cohorts (Figure S14). A comparison with the SPIEC‐EASI network also demonstrated the substantial reproducibility of the inferred microbial topology. Specifically, 50 of the 51 topological taxon nodes overlapped (98.04%), the same T1/T2 two‐community association pattern was reproduced, and 167 overlapping association pairs were observed (Table S15).

## 3 Discussion

In this study, we revealed the characteristic microbial association patterns defined by within‐group co‐occurrence and between‐group co‐exclusion in participants with CRA. These CRA‐related microbial association patterns comprised two distinct networks: a neoplasia‐associated network (including known CRC‐promoting species such as *C. symbiosum* and *H. hathewayi*), and a negatively associated network (including health‐associated species such as *Roseburia* sp. and *Lachnospira* sp.). The association patterns identified were validated using external datasets. These topological features demonstrate the potential for clinical applications across external cohorts. The T‐scores derived from these microbial association patterns showed the potential for CRN risk stratification, whereas discrimination models based on topological species demonstrated moderate performance, supporting the potential utility of the identified microbial association patterns. Integration analyses of microbial topologies and plasma metabolites suggested that alterations in microbial association patterns might be associated with tumorigenesis through changes in bile acids and microbiota‐derived metabolites. Collectively, our findings provide a novel perspective on the role of the gut microbiome in tumorigenesis and highlight microbial topology as a promising next‐generation biomarker.

We employed a network‐based analytical approach to investigate microbial topologies [18]. Species co‐occurrence and co‐exclusion relationships were identified using Fisher's exact test, revealing consistent association patterns in gut microbiota that reflect “seesaw” dynamics between the two networks in the development of CRA and CRC. Although 22,895 potential association pairs were initially identified, only 927 (approximately 4.05%) remained statistically significant after multiple testing corrections (Bonferroni‐adjusted *P* < 0.050). Under a more stringent threshold (Bonferroni‐adjusted *P* < 0.001), only 634 association pairs (approximately 2.77%) showed consistent directional effects across bootstrap iterations. This finding underscores that meaningful bacterial relationships are rare.

Among the two distinct microbial association networks identified (T1 and T2), the T1 network included several well‐documented neoplasia‐associated species, such as *H. hathewayi* [41], *C. symbiosum* [42], and *T. nexilis* [28], as well as species linked to intestinal inflammation, such as *Clostridium innocuum* [52, 53]. In contrast, the T2 topology was predominantly characterized by members of the *Lachnospiraceae* (10/37) and *Oscillospiraceae* (13/37) families. These taxa are primarily associated with SCFA production, and they demonstrate strong correlations with healthy immune homeostasis, thus offering protection against inflammatory disorders and CRC [54, 55, 56]. Comparing microbial species in the T1 and T2 networks with those in the Gut Microbiome Health Index [57] and Gut Microbiome Wellness Index 2 [58] revealed partial overlaps between T1 species and non‐health‐associated microbial markers (e.g., *Blautia producta* (*B. producta*) and *Hungatella* spp.). T2 species were found to be aligned with probiotic species, including *Oscillibacter* spp. *and Roseburia* spp. Both indices predict disease likelihood based on fecal microbiota. Moreover, a critical partial overlap was observed between our microbial association patterns and the immunotherapy efficacy‐associated microbial networks reported by Derosa et al. [18] in non‐small cell lung cancer. Species positively correlated with therapeutic efficacy showed partial alignment with our T2 species, whereas species negatively associated with treatment response partially overlapped with our T1 species. This overlap may indicate conserved microbial association patterns across the microecology of various tumors.

Our analysis also revealed paradoxical patterns in the microbial topology. Specifically, the T1 topology contained species that are associated with protective SCFA production or enrichment in healthy individuals, such as *B. producta* [59], which parallels the contradictions reported in previous studies. For instance, Derosa et al. observed that an immunotherapy‐suppressive network incorporated *B. producta*, which engaged in positive associations with other pathogenic bacteria, and was associated with reduced treatment efficacy [18]. Likewise, Wu et al. identified detrimental microbial guilds containing SCFA‐producing *Blautia* spp. in SparCC‐based co‐abundance network analysis [19, 60], and Chang et al. included pathogenic *Alistipes* spp. as health‐associated markers in the Gut Microbiome Wellness Index 2 [58, 61]. These discrepancies between single‐species functional characterizations and their roles within microbial topology suggest that network‐level associations may modify the functional effects of individual species or influence metabolite‐mediated effects, warranting further mechanistic investigations.

Building on CRA‐related microbial association patterns, we developed a streamlined topological score. The score is based on the proportions of T1 and T2 species present in each individual, reflecting their relative dominance within the gut microbiota. This score demonstrated consistent performance in our derivation set and across multiple external validation datasets, with significantly higher values in participants with CRA or CRC than in HC, indicating a shift toward a neoplasia‐associated microbial topology. The association between higher scores and CRA/CRC status underscores its potential utility in risk stratification. Furthermore, the incorporation of microbial associations through the MAS metric moderately improved the performance of CRC discrimination models that relied solely on single‐species abundance. This suggests that microbial topology captures complementary risk information beyond that of individual taxa, potentially reflecting a more stable signature of neoplasia‐associated microbiomes. This consistent performance across cohorts highlights the potential of topological features for population‐level CRN risk stratification, and supports further evaluation in larger CRC cohorts.

However, reduced generalizability was observed in the Japanese, Austrian, French, and Italian datasets, suggesting that geographic, ethnic, or environmental factors may influence microbial topology. Several factors may contribute to these discrepancies. First, although robust microbial topological relationships were identified in our derivation cohort, simplifying these complex networks into a unidimensional score inevitably obscured critical topological characteristics, such as association strength. This underscores the need to develop more sophisticated approaches that potentially involve SHAP‐based microbial networks to fully capture and utilize microbial topological information for future applications [62]. Second, regional variations in environmental factors, particularly diet, are important sources of gut microbiota heterogeneity [63, 64, 65]. Validation performance was lower in two datasets from populations with a Mediterranean dietary pattern (e.g., France and Italy) [66]. This diet, characterized by high consumption of vegetables, legumes, fruits, cereals, and olive oil, moderate fish intake, limited dairy (primarily cheese/yogurt) and meat, and modest wine consumption [67], differs substantially from the dietary patterns in the other study populations. It may therefore contribute to marked differences in gut microbial composition and topology [68, 69]. For instance, in populations following this diet, health‐promoting bacteria (*F. prausnitzii* and *Roseburia* spp.) are more abundant, whereas potentially harmful bacteria (such as *R. gnavus*) are less abundant [70, 71]. These microbial changes are also associated with elevated levels of beneficial SCFAs and reduced concentrations of harmful metabolic byproducts, including ethanol, p‐cresols, and carbon dioxide. Furthermore, differences among the cohorts in clinical features, such as colorectal tumor stage and anatomical location, may limit the generalizability of our findings.

Several sources of cross‐cohort heterogeneity warrant consideration when interpreting external validation results. Differences in sequencing platforms, library preparation protocols, and bioinformatics pipelines can introduce batch effects that affect microbial abundance profiles and association patterns [72, 73]. To partially mitigate these effects, all external datasets in our study were processed on Illumina platforms and uniformly profiled with MetaPhlAn (version 4.0) [74]. Additional variation may be due to differences in sample storage conditions, fecal sampling procedures, and DNA extraction methods across studies [74, 75]. Despite these challenges, the reproducibility of the identified microbial association patterns across cohorts indicates that the observed patterns are not solely artifacts of batch effects.

The CRA‐specific microbial association patterns identified in our study have potential implications for understanding CRN and future microbiome‐based applications. Our findings revealed stable relationships between T1 and T2 species, suggesting that microbial biomarkers incorporating multispecies association patterns may provide more robust and generalizable information than those based on individual species. Across multiple external datasets in which model performance ranged from moderate to good, several bacterial species consistently ranked among the most important features in the models. These findings suggest that these species may represent important microbial features associated with colorectal tumorigenesis, providing a basis for future mechanistic investigations. Furthermore, targeting groups of microorganisms with stable microbial association patterns may represent a promising strategy for microbiome‐based interventions, including the rational design of multi‐strain probiotic formulations [76, 77].

Altered microbial topology may be associated with CRN development through metabolite‐mediated host responses, such as epithelial barrier disruption, chronic inflammation, immune dysregulation, and carcinogenic signaling [78, 79, 80, 81, 82]. In our study, microbial topology‐associated metabolites were mainly related to the metabolism of tryptophan, bile acids, lysophospholipids, and amino acids/peptides. Tryptophan‐derived indole metabolites, including indole‐3‐propionic acid, may influence epithelial barrier integrity and mucosal immune homeostasis, thereby potentially modulating early neoplastic transformation [83, 84]. Microbiota‐modified bile acids can regulate inflammatory signaling, epithelial stress responses, and antitumor immunity in the colorectal microenvironment [85]. In addition, lipid‐related metabolites, including lysophospholipids and fatty acid metabolites, may reflect host–microbiota metabolic remodeling and may be associated with the proliferative and inflammatory signaling pathways involved in colorectal carcinogenesis [86]. Collectively, these metabolite alterations may contribute to increased CRN risk. Based on these findings, we propose a potential microbiota–metabolite–host axis linking gut microbiota topology alterations with CRN development (Figure S15). However, the causal mediation analysis did not identify any individual metabolite as a significant mediator, suggesting that the association between microbial topology and CRN is likely mediated through a complex network of interacting microbial, metabolic, and host factors rather than a single metabolite. Therefore, the proposed axis should be regarded as a conceptual framework based on observed associations rather than demonstrated causality. Further validation using high‐dimensional mediation analyses and subsequent in vivo functional studies is warranted.

In conclusion, using a large multicenter CRC screening cohort from China, we demonstrated that neoplasia‐related microbial association patterns are characterized by a seesaw‐like topological organization, thus providing new insights into microbial alterations during CRN development. These topology‐derived features, including the T‐score, demonstrated discriminatory ability for non‐invasive CRN risk stratification and were reproducibly observed across multiple external cohorts from different geographic regions. Their application, however, particularly for CRC discrimination, remains exploratory and requires further validation in larger prospective cohorts. Performance also varied across populations, potentially influenced by geographic and dietary variations in gut microbiota composition. This highlights the need for population‐specific recalibration before clinical translation.

### 3.1 Limitations of the Study

Although we identified CRA‐related microbial association patterns and validated topology scores across multiple independent cohorts, several important limitations of this study should be acknowledged. First, while our analysis leveraged metagenomic sequencing data, the resolution was constrained to species‐level taxonomy. Strain‐level topological analysis may yield additional insights into microbial ecological networks. Second, our current framework does not fully incorporate key network features such as pairwise association strengths. These limitations highlight the need for advanced analytical methods to better characterize higher‐order microbial associations in participants with CRN [87, 88]. Third, the limited number of CRC cases in our internal cohort reduced the robustness of the CRC‐specific conclusions. Although the large external CRC dataset from our collaborating center partially mitigates this limitation, residual batch effects and inter‐cohort heterogeneity cannot be excluded. Fourth, several potentially relevant covariates, such as defecation habits and recent gastrointestinal infections, were unavailable in the external cohort and could not be adjusted. Finally, although experimental studies have validated the effects of individual species on the development of neoplasia within these topological groups, the collective ecological and mechanistic effects of these complex polymicrobial networks on colorectal carcinogenesis remain to be elucidated.

## 4 Methods

### 4.1 Study Population and Sample Collection

This study was based on the multicenter TARGET‐C cohort (ChiCTR1800015506) that was initiated in May 2018 across five provinces in China (Jiangsu, Zhejiang, Anhui, Hunan, and Yunnan). A total of 19 373 asymptomatic participants aged >50 years underwent colonoscopy‐based CRC screening. Details of the study protocol have been previously published [89, 90, 91]. Participants aged 50–74 years were recruited via community outreach and excluded if they had prior CRC, colorectal surgery, cancer treatment, recent CRC screening, lower gastrointestinal symptoms requiring diagnostic evaluation, or comorbidities that contraindicated screening.

A total of 5179 stool samples were collected within 24 h prior to bowel preparation, following standardized protocols. Written informed consent was obtained from all the participants, and the study was approved by the Ethics Committee of the National Cancer Center (approval number: 18‐013/1615).

### 4.2 External Metagenomic Datasets

To validate our findings, we further obtained raw metagenomic sequencing data from eight independent datasets (totaling 582 participants with CRC, 181 adenoma cases, and 621 HCs) from the Sequence Read Archive and the European Nucleotide Archive: China (CHN, PRJEB10878, 74 CRC cases, 54 HCs) [92], India (IND, PRJNA531273, 30 CRC cases, 30 HCs) [93], Japan (JPN, PRJDB4176, 245 CRC cases, 67 CRA cases, 252 HCs) [94], the United States (USA, PRJEB12449, 52 CRC cases, 52 HCs) [95], Austria (AUS, ERP008729, 46 CRC cases, 47 CRA cases, 61 HCs) [96], Italy (ITA, PRJNA447983, 61 CRC cases, 27 CRA cases, 52 HCs) [51], France (FRA, ERP005534, 52 CRC cases, 40 CRA cases, 60 HCs) [97], and Germany (GER, PRJEB27928, 22 CRC cases, 60 HCs) [98]. The metadata were manually curated from relevant original publications and referred to in two published articles [99, 100]. Only samples from participants with CRC, adenoma, and HC were retained (Table S9).

To examine whether the identified topological structures extended to CRC participants, we analyzed an additional external cohort of 460 CRC participants from a collaborating center in Guangzhou, China (Genome Sequence Archive accession HRA004617) [41].

### 4.3 DNA Extraction, Shotgun Metagenomic Sequencing, and Taxonomic Profiling

Stool samples were collected in fecal collection tubes and stored at −80°C until use. Fecal DNA was extracted using an MGIEasy Fecal DNA (meta) sample collection kit (MGI Tech, Shenzhen, China). Sequencing libraries were generated using the MGIEasy Digesting DNA Library Preparation Kit (V2.0; MGI Tech, Shenzhen, China). After library quality control, high‐throughput 2 × 150 bp paired‐end sequencing was performed using the MGI platform. Shotgun metagenomic sequencing of stool samples generated a total of 86.5 TB of high‐quality data (quality control was conducted following the metapi workflow [https://github.com/ohmeta/metapi]), with an average yield of 16.7 GB per sample.

The MetaPhlAn 4.0 tool (https://huttenhower.sph.harvard.edu/metaphlan/, database mpa_vJun23_CHOCOPhlAnSGB_202403) was used to profile the composition of the microbial communities in our cohort and in the external datasets [22, 101]. A total of 3637 bacterial species were identified in our cohort. Of these, 600 species were detected in more than 5% of the participants.

We excluded 1372 samples: (i) 16 samples with low alignment reads (<1 000 000) [102]; (ii) 16 samples with a non‐host read ratio below 70% (upper bound of outliers); (iii) 1 sample with a duplication rate greater than 20% (upper bound of outliers); (iv) 466 samples with a relative abundance of *Escherichia coli* > 5% (threshold set according to the upper bound of outliers observed in external CRC datasets to minimize potential contamination); (v) 660 participants with missing values for any clinical characteristic (including those who ultimately declined colonoscopy); (vi) 114 samples collected at follow‐up rather than baseline; (vii) 99 participants with serrated adenomas, polyps without pathological confirmation, or neuroendocrine tumors. In the end, 3807 samples remained.

### 4.4 Alpha and Beta Diversity Analysis

All analyses were performed using R 4.3.1 (R Foundation for Statistical Computing, Vienna, Austria). For microbial characteristics across different groups, α‐diversity metrics (within‐sample diversity), including the Shannon index and richness, were calculated using the vegan package (v2.6.10), with ANCOVA adjusted for sex, age, BMI, family history of CRC, recruitment region, alcohol consumption, smoking status, hypertension, and diabetes (hereafter referred to as covariates) [23]. β‐diversity (between‐sample diversity) was assessed using Bray–Curtis dissimilarity via the vegan package (v2.6.10). PERMANOVA (999 permutations) using species‐level taxonomic profiles was employed to evaluate microbial compositional differences across groups, and the results were visualized using principal coordinate analysis ordination plots [103, 104]. Enterotype analysis was performed according to an established method [105].

### 4.5 Differential Abundance Analysis

Differential abundance analysis was applied to 600 species (16.50% of 3637 detected species) that were present in >5% of individuals [106, 107, 108, 109]. Differential species between groups were identified using LEfSe implemented via the microeco package (v1.13.19) [26, 110], with significance thresholds set at an absolute LDA score (log10) ≥ 2, and nominal *P* < 0.050 [25]. ANCOM‐BC2 [32] was used to identify differential abundance changes across disease groups after adjusting for covariates. Statistical significance was defined as Benjamini–Hochberg FDR < 0.050. This method internally applies a log transformation with a pseudo‐count of 1e‐07 to the relative abundance data. MaAsLin2 [36] was applied to log‐transformed relative abundance data (with a pseudo‐count of 1e‐07), as implemented in the MaAsLin package, to assess the association between the gut microbiota and CRN (FDR < 0.050). To correct for an imbalance between colorectal cancer cases and controls in our dataset, MaAsLin2 was conducted after 1: 3 nearest‐neighbor matching by age, sex, and BMI. The MaAsLin2 model was adjusted for sex, age, and BMI in our cohort and external datasets to identify microbial signatures, followed by cross‐cohort comparisons.

### 4.6 Co‐Abundance and Co‐Occurrence Network Construction

Co‐abundance networks were constructed for species with prevalence >5% in each group using SparCC, implemented as fast sparCC in Python (https://github.com/shafferm/fast_sparCC) [35]. Significant co‐abundance associations were defined as those with an absolute correlation coefficient >0.30 and an FDR < 0.05 based on 100 permutations [111, 112]. Network topology metrics were calculated using the package igraph (v2.1.4) in R. Hub scores were calculated by integrating degree, betweenness, closeness, and eigenvector centralities into a weighted composite metric, and the top 10 species with the highest scores were identified in each network [113, 114]. Co‐abundance networks were visualized using the package visNetwork (v2.1.2) [115].

To characterize CRA‐related microbial association patterns, we applied the framework proposed by Derosa et al. [18], which constructs microbial topologies from species co‐occurrence and co‐exclusion relationships rather than abundance correlations. We randomly divided our cohort into derivation and validation sets in a 5:5 ratio and focused exclusively on participants with conventional CRA in the derivation set, as the limited number of CRC cases might have introduced a potential bias in the identification of microbial association patterns. To obtain reproducible results, the set.seed() function was used for dataset division.

After excluding microbial species with a prevalence <5% in participants with CRA [106, 107, 108, 109], we dichotomized the relative abundance of each remaining bacterial species in every participant as either ‘low’ (≤median abundance in healthy controls) or ‘high’ (>median). When a species exhibited predominantly zero abundance (i.e., median = 0), this approach effectively corresponded to a binary classification of ‘absence’ versus ‘presence’. We initially selected species consistently related to CRA risk based on effect size thresholds using GLM with 10 iterative repetitions (neoplasia‐associated: OR ≥ 1.10; negatively associated: OR ≤ 0.80), without considering statistical significance to maximize sensitivity for potential associations. The selected species were subsequently analyzed to identify CRA‐related microbial association patterns based on species co‐occurrence and co‐exclusion relationships.

For these selected microbial species, we reclassified their abundance in the derivation set as ‘absence’ (relative abundance = 0) or ‘presence’ (relative abundance > 0), following the approach described by Derosa et al. [18]. Using Fisher's exact tests, we analyzed all possible pairwise combinations of these species to identify co‐occurrence or co‐exclusion relationships specifically in participants with CRA (to investigate neoplasia‐related microbial associations). Each microbial association was quantified by an association score: −log10(*P*) × sign (*OR* − 1), where *P* represents Fisher's exact test *P‐*value and OR is the odds ratio derived from the 2 × 2 table. Negative scores indicated co‐exclusion relationships (*OR *< 1), whereas positive values indicated co‐occurrence patterns (*OR* > 1). To ensure the robustness of the identified microbial association patterns, we performed 50 bootstrap iterations [116, 117, 118]. Instead of the previously used FDR method, the Bonferroni correction was employed here to select more stable microbial associations and reduce the likelihood of false‐positive results [119]. Only associations meeting both of the following criteria were considered: consistency, defined as a Bonferroni‐adjusted *P* < 0.050 across all iterations; and network relevance, defined as participation in >5 significant associations. These robust associations were used for the subsequent analyses.

### 4.7 The Identification and Validation of Microbial Association Patterns

Using the association scores obtained from Fisher's exact test, we first calculated the median association score for each microbial association pair. These scores were then subjected to hierarchical clustering using Ward's method with Manhattan distance. Based on the resulting clusters, we further classified them into distinct microbial network groups according to the proportions of neoplasia‐associated and negatively associated species within each cluster. This final classification yielded two primary microbial association patterns: a CRN‐associated network (T1 topology) and a negatively associated network (T2 topology) [18]. Co‐occurrence networks were visualized using the visNetwork package (v2.1.2) [115].

We validated the identified microbial association patterns in both the validation and public datasets. Because most public datasets contain only a limited number of participants with CRA and CRC (fewer than 100 in most cases), we combined participants from eight public datasets to create an integrated neoplasia cohort for validation. First, the species were preprocessed using the same strategy based on relative abundance and categorized into ‘presence’ and ‘absence’. Fisher's exact test was then employed to validate the co‐occurrence and co‐exclusion relationships between the topological species. Association pairs with Bonferroni‐adjusted *P* < 0.050 were retained and visualized using the package visNetwork (v2.1.2) [115].

For sensitivity analyses, we re‐performed the GLM‐based species preselection using three alternative effect‐size thresholds for neoplasia‐associated and negatively associated species, respectively: (a) OR ≥ 1.10 and ≤0.90; (b) OR ≥ 1.05 and ≤0.95; and (c) OR > 1 and <1. Co‐occurrence networks were reconstructed from preselected species and visualized using the visNetwork R package (v2.1.2) [115].

In addition to the SparCC network, SPIEC‐EASI was used to assess the reproducibility of microbial network nodes and associations, as it was specifically designed for compositional data [120]. SPIEC‐EASI networks were inferred using the SpiecEasi R package based on unnormalized count data. Network inference was performed using the sparse Meinshausen–Bühlmann neighborhood selection method (mb) [120, 121]. Given the relatively high density of the networks, the scaling factor controlling the minimum sparsity level (lambda.min.ratio) was set to 0.001. To approach the target stability threshold of 0.050, the number of regularization parameters (nlambda) was set to 50.

### 4.8 Quantification and Clinical Potential of Microbial Topology

Based on the presence of species belonging to the T1 and T2 topologies, we derived a unidimensional topology score (T‐score) to quantify each participant's CRA‐related microbial network profile [18]. Specifically, the number of detected T1 species in each participant was divided by the total number of T1 topology species (NT1 = 14), while the number of detected T2 species was divided by the total number of T2 topology species (NT2 = 37). The T‐score was calculated as follows: T‐score = (#T1/NT1 − #T2/NT2 + 1)/2, where #T1 and #T2 represent the numbers of T1 and T2 topology species with relative abundance >0 in an individual participant's stool sample, respectively, and NT1 and NT2 represent the total numbers of species included in the corresponding topologies. A brief schematic workflow summarizing the major analytical steps, from species‐level microbial profiling to T‐score calculations, is shown in Figure S16.

The T‐scores ranged from 0 to 1. A score of 1 indicated that all species in the T1 topology were detectable but none in the T2 topology, whereas a score of 0 indicated the opposite. Thus, higher T‐scores reflect a stronger representation of the neoplasia‐associated microbial topology. For our derivation, validation, and external public datasets, we employed a covariate‐adjusted GLM based on each participant's topology score to validate its correlation with CRA or CRC risk. Models were adjusted for sex, age, BMI, family history of CRC, recruitment region, alcohol consumption, smoking status, hypertension, diabetes, dietary pattern, and regular use of antibiotics and probiotics in our cohort, and for age, sex, and BMI in the external datasets.

Based on the identified CRA‐related microbial association patterns, we constructed random forest classification models (1000 trees) with leave‐one‐out cross‐validation using both the relative abundances and presence/absence status of topology‐derived microbial features [19]. These models were developed using external datasets, aiming to evaluate the discriminatory performance of topological species in distinguishing participants with CRC from HCs. Performance was evaluated using discrimination (AUC) and calibration (Spiegelhalter's z‐test) [122, 123, 124]. Recalibration was performed using the Platt scaling method [122, 125]. To evaluate the contribution of individual topological species to model performance, variable importance was calculated using the varImp function in the caret package (v6.0.94), scaled to a comparable range. The top 20 features were ranked based on their overall importance.

Based on the overlapping and directionally consistent association pairs identified from both the co‐abundance network (via SparCC) and the co‐occurrence network (via Fisher's exact test), we developed a novel MAS metric to quantify microbial association patterns and strengths. For each individual, the MAS was calculated by summing the β‐coefficients derived from the log‐transformed mean ORs of all association pairs (based on Fisher's exact tests for co‐occurrence), with inverted coefficients applied to association pairs between two T2 species (negatively associated with neoplasia). The same predefined algorithm was applied to independently calculate the MAS for each external dataset. A higher positive MAS value indicated a stronger neoplasia‐associated microbial topology within the gut microbiome.

We evaluated whether incorporating MAS as an independent predictor in multivariable logistic regression models could improve discriminatory power. The comparison models were based solely on three CRC‐associated species, which were selected for their strong cross‐cohort reproducibility, literature support, and strength of association with CRC [50, 51].

### 4.9 Identification of Dietary Patterns

To adjust for confounding dietary factors, a posteriori dietary patterns were derived from nine food groups using factor analysis with principal component extraction. Detailed information on dietary assessment and pattern derivation was described in our previous study based on this cohort [126]. Briefly, orthogonal varimax rotation was applied to obtain a mutually independent factor structure and improve interpretability. The optimal number of factors was determined by combining multiple criteria: scree plot inspection, parallel analysis (comparing the observed data to random matrices), factor interpretability, and the proportion of variance explained. A three‐factor solution was selected. Based on the resulting weighted factor scores, participants were assigned to one of the three dietary pattern groups using k‐means clustering.

### 4.10 Plasma Metabolome Profiling and Data Analysis

Liquid chromatography–tandem mass spectrometry‐based untargeted metabolomic profiling was applied to plasma samples from 806 participants in our multicenter cohort, matched to the fecal samples analyzed in this study. Samples were collected from 255 participants with NAA, 279 participants with AA, and 272 HC. Detailed procedures for sample collection and handling were described in a previous publication [89]. Briefly, thawed plasma samples were centrifuged, and the resulting supernatants were used for metabolite detection. Raw data were processed to extract the exact molecular mass (*m*/*z*) values, followed by annotation against the HMDB database and rigorous quality control procedures that were performed according to established protocols [127, 128]. Peak area data for annotated metabolites were used in subsequent analyses. A total of 681 metabolites were identified. Metabolite measurements were set to missing if they were below the lower limit of detection or quantification, or if they were identified as outliers (interquartile range method) [129]. Stringent quality filters excluded metabolites with a coefficient of variation > 30% in quality control samples based on the raw peak area, as well as those with a missing rate exceeding 80% across all samples. After filtering, 643 high‐quality metabolites were retained for downstream statistical analysis. The remaining missing values were imputed with the median [130].

For the integrated analysis, the center with the largest sample size in our cohort (Taizhou Center in Zhejiang Province; *n* = 346) was designated as the derivation set, whereas participants from the remaining centers were used for validation. In the derivation set, Spearman's correlation analysis was performed to identify plasma metabolites associated with the T‐score. *P*‐values were adjusted using the Benjamini–Hochberg method, with FDR < 0.050 considered statistically significant. Among the significant metabolites, the top 50, ranked by the absolute values of their Spearman correlation coefficients, were subsequently evaluated for validation. Multivariable linear regression analyses were performed separately within each validation center, adjusting for age, sex, BMI, family history of CRC in first‐degree relatives, smoking status, alcohol use, diabetes, hypertension, dietary pattern, and regular use of antibiotics and probiotics. Center‐specific effect estimates and the corresponding standard errors were pooled using a random‐effects meta‐analysis. Metabolites with a pooled *P*‐value < 0.050 were considered significantly associated with the T‐score.

## Author Contributions

K.S.: conceptualization, data curation, formal analysis, and writing the original draft. Y.W.Q.: conceptualization, data curation, formal analysis and writing the review & editing. J.H.L., and L.P.L.: conceptualization, data curation, and formal analysis. C.Y.L., Y.B.Q., Y.Y.Z., H.Z.Z., K.W., M.N., and D.W.: data curation and writing the review & editing. S.D.Z., M.D. and H.D.C.: conceptualization, data curation, funding acquisition, and writing the review & editing.

## Conflicts of Interest

The authors declare no conflicts of interest.

## Supporting information




**Supporting File**: advs77991‐sup‐0001‐SuppMat.docx.

## Data Availability

The raw metagenomic sequence data reported in this paper have been deposited in the Genome Sequence Archive at the National Genomics Data Center, China National Center for Bioinformation / Beijing Institute of Genomics, Chinese Academy of Sciences, and are publicly accessible at https://ngdc.cncb.ac.cn/gsa/ (accession number: CRA026961). Additional information required to reanalyze the data reported in this study is available upon request.
