# Supplementary material for: Gut Microbial Topology and Metabolic Signatures Associated With Colorectal Neoplasia
Source: Adv Sci (Weinh). 2026 Sep 28:e77991. Online ahead of print. doi: 10.1002/advs.77991 (PMC13620265; doi:10.1002/advs.77991)
Supplement: Supplementary file 1 [file ADVS-9999-e77991-s001.docx]

## Supplemental information

##### Figure S1. Overall patterns of gut microbiome in healthy control, colorectal adenoma and cancer.

(A, B) The alpha diversity of different groups was measured using the Shannon index and richness. The adjusted *P* value (two-sided test) was calculated using analysis of covariance (ANCOVA) adjusted for sex, age, body mass index, family history of colorectal cancer, recruitment region, alcohol consumption, smoking status, hypertension, diabetes. (C) Principal coordinate analysis (PCoA) based on species abundance profiles, with group differences tested by PERMANOVA (999 permutations, two-sided). (D) PCoA plot based on Bray–Curtis distances revealed two enterotypes. The optimal number of clusters was determined using Partitioning Around Medoids (PAM) clustering with silhouette width analysis, which identified two enterotypes. Each point represents an individual sample, and shaded ellipses denote the 80% confidence interval for each enterotype cluster. (E) Genus-level composition across different enterotypes, shown as percentage stacked bar plots. (F) Comparison of the relative abundances of the four key genera distinguishing the enterotypes. (G) PCoA plot based on Bray–Curtis distances of species-level relative abundance profiles after stratifying patients by enterotype. The R^2^ value shown in the plot indicates the proportion of variance in microbial community structure explained by disease diagnosis, as assessed by PERMANOVA.

##### Figure S2. Comparison of microbial species across groups. Related to Figure 2

(A) The profiles of the top 15 species, identified according to their mean relative abundance in the healthy control group, across all four study groups. (B) The profiles of the top 15 species, identified according to their prevalence in the healthy control group, across all four study groups. Species shown in bold represent taxa that are shared between (A) and (B). Differential bacterial species between patients with advanced adenoma (C) or non-advanced adenoma (D) and healthy controls identified by linear discriminant analysis (LDA) effect size (LEfSe) (LDA score >2.0, p <0.05, false discovery rate <20%). Species shown in bold represent taxa that are shared between (C) and (D). (E) Pairwise comparison of differential species using Analysis of Compositions of Microbiomes with Bias Correction 2 (ANCOM-BC2), applied to differential species identified by LefSe. The pairwise comparisons were conducted among three groups: healthy controls vs. non-advanced adenoma, healthy controls vs. advanced adenoma, and non-advanced adenoma vs. advanced adenoma. AA, advanced adenoma; HC, healthy control; NAA, non-advanced adenoma.

##### Figure S3. Co-abundance network of gut microbiome in CRN.

Co-abundance network of microbial species in HC (A) and CRA (B) calculated by SparCC. Significant co-abundance was displayed with correlation coefficient > 0.30 and false discovery rate (FDR) < 5% using 100× permutation. Only species connected to the top 10 species with highest scores are shown. Red nodes represent the top 10 species within the correlation network. Cyan nodes indicate taxa present in both networks, green nodes denote taxa unique to the healthy control network, and yellow nodes denote taxa unique to the adenoma network. Red edges represent positive associations, whereas blue edges represent negative associations.

##### Figure S4. Topologically central species in the co-abundance networks of healthy controls and colorectal adenomas.

The top 10 topologically central species in the co-abundance networks of healthy controls (A) and colorectal adenomas (B) groups are shown, based on hub scores calculated from a weighted combination of degree centrality, betweenness centrality, closeness centrality, and eigenvector centrality. The topologically central species in the co-abundance networks of healthy controls (C) and colorectal adenomas (D) groups are shown, based on within-module (Zi) and among-module (Pi) connectivity.

**Figure S5. CRA-related microbial association patterns in validation set.**

CRN-related microbial association patterns identified in the derivation set exhibit robust associations in patients with CRN from the validation set. Edges represent significant pairwise associations identified by Fisher’s exact test with Bonferroni correction (adjusted *P* < 0.05). Red and blue lines indicate co-occurrence and co-exclusion relationships, respectively. Orange and green nodes represent species belonging to the neoplasia-associated (T1) and negatively associated (T2) networks, respectively.

**Figure S6. CRA-related microbial association patterns in sensitivity analyses.**

CRN-related microbial association patterns identified in the derivation set exhibited robust associations in patients with CRN in sensitivity analyses based on GLM-based species preselection thresholds (neoplasia-associated: ≥ 1.10; negatively associated: OR ≤ 0.90). Edges represent significant pairwise associations identified by Fisher's exact test with Bonferroni correction (adjusted *P* < 0.05). Red and blue lines indicate co-occurrence and co-exclusion relationships, respectively. Orange and green nodes represent species belonging to the neoplasia-associated (T1) and negatively associated (T2) networks, respectively, while grey nodes indicate species not included in the neoplasia-related microbial association patterns.

**Figure S7. CRA-related microbial association patterns in sensitivity analyses.**

CRN-related microbial association patterns identified in the derivation set exhibited robust associations in patients with CRN in sensitivity analyses based on GLM-based species preselection thresholds (neoplasia-associated: ≥ 1.05; negatively associated: OR ≤ 0.95). Edges represent significant pairwise associations identified by Fisher's exact test with Bonferroni correction (adjusted *P* < 0.05). Red and blue lines indicate co-occurrence and co-exclusion relationships, respectively. Orange and green nodes represent species belonging to the neoplasia-associated (T1) and negatively associated (T2) networks, respectively, while grey nodes indicate species not included in the neoplasia-related microbial association patterns.

**Figure S8. CRA-related microbial association patterns in sensitivity analyses.**

CRN-related microbial association patterns identified in the derivation set exhibited robust associations in patients with CRN in sensitivity analyses based on GLM-based species preselection thresholds (neoplasia-associated: > 1.00; negatively associated: OR < 1.00). Edges represent significant pairwise associations identified by Fisher's exact test with Bonferroni correction (adjusted *P* < 0.05). Red and blue lines indicate co-occurrence and co-exclusion relationships, respectively. Orange and green nodes represent species belonging to the neoplasia-associated (T1) and negatively associated (T2) networks, respectively, while grey nodes indicate species not included in the neoplasia-related microbial association patterns.

**Figure S9. CRA-related microbial association patterns among topological species in patients with CRN from external dataset.**

Species in colorectal neoplasia-related microbial association patterns identified in the derivation set exhibit robust associations (Bonferroni adjusted *P* < 0.05) in the patients with CRA (A), CRC (B) from the integrated external dataset and patients with CRC from Guangzhou center (C). Orange nodes represent species in the neoplasia-associated network (T1), while light blue nodes represent species in the negatively associated network (T2). Blue and red edges represent co-exclusion and co-occurrence relationships, respectively.

**Figure S10. Comparison of topology scores between patients with CRN and healthy controls across external datasets.**

Comparison of topology scores (T-scores) between the various groups in the external datasets from India, the United States, France, Italy, Germany, China, Japan and Australia (A-H).

**Figure S11. Performance of the random forest model based on topological species for distinguishing CRC. Related to Figure 6.**

(A, B) Random forest models (leave-one-out cross-validation, LOOCV, 1000 trees) based on the presence/absence status of topological species for distinguishing CRC from the United States and Japan.

(C, D) Random forest models based on the relative abundance of topological species for distinguishing CRC from the United States and Japan.

**Figure S12. Decision curve analysis of the random forest model based on topological species for distinguishing CRC.**

(A-F) Decision curve analysis of random forest models based on the presence/absence status of topological species for distinguishing CRC across different datasets. Panels (A) through (F) represent the results for France, Austria, India, Germany, Italy, and China, respectively.

(G-L) Decision curve analysis of random forest models based on the relative abundance of topological species for distinguishing CRC across different datasets. Panels (A) through (F) represent the results for France, Austria, India, Germany, Italy, and China, respectively.

**Figure S13. Performance of the discriminatory model based on topological species and demographic data for CRC.**

(A) Performance of random forest models based on the presence/absence of topological species (leave-one-out cross-validation [LOOCV], 1000 trees) in datasets from India, Germany, Austria, France, and China. (B) Models based on the relative abundance of species (LOOCV, 1000 trees) in the same cohorts. The dataset from Italy was excluded from this analysis due to extensive missing values in demographic data.

**Figure S14. Comparison of CRC discrimination performance across cohorts by integrating microbial association strength with CRC-associated species.**

Using three cross-cohort colorectal cancer-associated species with the strongest correlations identified from the literature, models were evaluated separately in external cohorts from Australia (A), India (B), Italy (C), the United States (D), Germany (E), China (F), France (G), and Japan (H).

For each cohort, seven models were constructed: Model 1: Based on demographic variables only (age, body mass index, sex); Model 2: Model 1 plus relative abundance of *Fusobacterium nucleatum*; Model 3: Model 1 plus relative abundance of *Parvimonas micra*; Model 4: Model 1 plus relative abundance of *Gemella morbillorum*; Model 5: Model 2 plus the Microbial Association Strength (MAS) metric; Model 6: Model 3 plus the MAS metric; Model 7: Model 4 plus the MAS metric. AUC, area under the receiver operating characteristic curve.

**Figure S15. Schematic diagram of the proposed microbiota–metabolite–host axis linking gut microbial association patterns, plasma metabolites, and colorectal neoplasia risk.**

**Figure S16. Schematic workflow of microbial association patterns identification and T-score calculation.**

##### Table S1. Baseline characteristics of participants.

| **Variable** | **Healthy control (n = 2,725)** | **Non-advanced adenoma**  **(n = 759)** | **Advanced adenoma**  **(n= 297)** | **Colorectal cancer**  **(n = 26)** |
| --- | --- | --- | --- | --- |
| Age, mean (SD) | 59.59 (6.26) | 60.73 (6.15) | 61.25 (6.04) | 61.88 (6.88) |
| Female/Male | 1205 / 1520 | 460 / 299 | 205 / 92 | 17 / 9 |
| BMI (kg/m^2^), mean (SD) | 24.07 (2.88) | 24.33 (2.81) | 24.52 (2.99) | 25.21 (2.31) |
| Family history of CRC in first-degree relatives, n (%) | 260 (9.54) | 77 (10.14) | 39 (13.13) | 6 (23.08) |
| Smoking status, n (%) |  |  |  |  |
| Never | 2028 (74.42) | 511 (67.33) | 168 (56.57) | 15 (57.69) |
| Ever | 165 (6.06) | 49 (6.46) | 22 (7.41) | 2 (7.69) |
| Sometimes | 76 (2.79) | 27 (3.56) | 15 (5.05) | 0 (0.00) |
| Every day | 456 (16.73) | 172 (22.66) | 92 (30.98) | 9 (34.62) |
| Drinking frequency, n (%) |  |  |  |  |
| Never | 1740 (63.85) | 491 (64.69) | 146 (49.16) | 17 (65.38) |
| Once a month or less | 272 (9.98) | 68 (8.96) | 34 (11.45) | 3 (11.54) |
| 2–4 times per month | 187 (6.85) | 52 (6.85) | 24 (8.08) | 0 (0.00) |
| 2–3 times per week | 134 (4.92) | 33 (4.35) | 15 (5.05) | 1 (3.85) |
| 4 or more times per week | 392 (14.39) | 115 (15.15) | 78 (26.26) | 5 (19.23) |
| Diabetes mellitus, n (%) | 164 (6.02) | 53 (6.98) | 23 (7.74) | 3 (11.54) |
| Hypertension, n (%) | 608 (22.31) | 170 (22.40) | 90 (30.30) | 8 (30.77) |
| Regular use of antibiotics, n (%) | 44 (1.61) | 6 (0.79) | 1 (0.34) | 0 (0.00) |
| Regular use of probiotics, n (%) | 33 (1.21) | 6 (0.79) | 1 (0.34) | 0 (0.00) |
| Dietary pattern, n (%) |  |  |  |  |
| Cluster A | 1,026 (37.65) | 322 (42.42) | 138 (46.46) | 13 (50.00) |
| Cluster B | 789 (28.95) | 204 (26.88) | 96 (32.32) | 7 (26.92) |
| Cluster C | 910 (33.39) | 233 (30.70) | 63 (21.21) | 6 (23.08) |

##### BMI: body mass index; SD: standard deviation. Table S2. Dietary patterns identified by K-means clustering.

| **Food group** | **Cluster A** | **Cluster B** | **Cluster C** |
| --- | --- | --- | --- |
| Red meat | 8.58 ± 3.82 | 5.20 ± 3.71 | 6.73 ± 3.73 |
| White meat | 6.03 ± 3.91 | 2.02 ± 2.00 | 5.37 ± 3.51 |
| Eggs | 4.51 ± 2.77 | 2.44 ± 2.56 | 7.04 ± 3.39 |
| Dairy | 1.07 ± 1.95 | 0.31 ± 1.02 | 5.97 ± 3.60 |
| Processed meat | 3.08 ± 3.98 | 0.10 ± 0.52 | 0.24 ± 1.01 |
| Refined grain | 13.63 ± 1.64 | 12.56 ± 3.40 | 8.83 ± 4.38 |
| Whole grain | 3.20 ± 2.95 | 1.33 ± 2.32 | 6.69 ± 3.94 |
| Fruits | 4.83 ± 4.34 | 3.04 ± 3.52 | 7.79 ± 4.03 |
| Vegetables | 12.87 ± 2.68 | 11.39 ± 3.86 | 9.13 ± 4.14 |

##### Table S3. ANCOM-BC2 Analysis of Species-Level Microbial Abundance Across Groups. Related to Figure 2.

| **Species** | **Log FC (NAA vs. HC)** | **Log FC (AA vs. HC)** | **SE (NAA vs. HC)** | **SE (AA vs. HC)** | ***P* value^*^** | **Adjusted *P* value^#^** | **Sensitivity analysis** |
| --- | --- | --- | --- | --- | --- | --- | --- |
| *Bacteroides eggerthii* | 0.26 | -0.68 | 0.12 | 0.19 | ＜0.001 | ＜0.001 | Passed |
| *Faecalibacterium SGB15346* | -0.04 | -0.30 | 0.10 | 0.17 | ＜0.001 | ＜0.001 | Not passed |
| *Faecalibacterium prausnitzii* | -0.16 | -0.36 | 0.12 | 0.19 | ＜0.001 | ＜0.001 | Passed |
| *GGB9715 SGB15267* | 0.00 | 0.30 | 0.11 | 0.17 | ＜0.001 | ＜0.001 | Not passed |
| *Jilunia laotingensis* | 0.13 | 0.30 | 0.10 | 0.17 | ＜0.001 | ＜0.001 | Passed |
| *Klebsiella aerogenes* | -0.02 | -0.53 | 0.11 | 0.18 | ＜0.001 | ＜0.001 | Passed |
| *Klebsiella quasipneumoniae* | 0.49 | 0.33 | 0.11 | 0.18 | ＜0.001 | ＜0.001 | Passed |
| *Klebsiella variicola* | 0.30 | -0.24 | 0.11 | 0.17 | ＜0.001 | ＜0.001 | Passed |
| *Megasphaera sp NM10* | 0.48 | 0.48 | 0.11 | 0.18 | ＜0.001 | ＜0.001 | Passed |
| *Oscillibacter sp ER4* | -0.06 | -0.35 | 0.11 | 0.18 | ＜0.001 | ＜0.001 | Passed |
| *Parabacteroides goldsteinii* | -0.07 | -0.46 | 0.11 | 0.18 | ＜0.001 | ＜0.001 | Passed |
| *Phocaeicola dorei* | -0.40 | -0.73 | 0.16 | 0.25 | ＜0.001 | ＜0.001 | Passed |

* Adjusted for sex, age, BMI, family history of CRC, recruitment region, alcohol consumption, smoking status, hypertension, diabetes.

\# Adjusted by Benjamini - Hochberg correction.

AA, advanced adenoma; FC, fold change; HC, healthy control; NAA, not advanced adenoma; SE, standard error.

##### Table S4. Alterations of species-species networks across different groups. Related to Figure S3.

| **Network properities** | **Healthy Control^*^** | **Colorectal Adenoma** |
| --- | --- | --- |
| Nodes (N) | 416 | 353 |
| Edges (N) | 3011 | 2594 |
| Edge density | 0.03 | 0.04 |
| Average degree | 14.47 | 14.70 |
| Diameter | 3.23 | 3.00 |
| Average path length | 1.08 | 1.01 |
| Clustering coefficient | 0.44 | 0.45 |
| Modularity | 0.45 | 0.47 |

N, number.

* To minimize potential confounding effects from covariates, patients in the healthy control group were matched 1:1 with those in the colorectal adenoma group using nearest neighbor matching based on age, sex, BMI, and family history of CRC.

##### Table S5. Top 100 species and network topology metrics in significant networks across different groups. Related to Figure S4.

| **Species** | **Degree centrality** | **Betweenness centrality** | **Closeness centrality** | **Eigenvector centrality** | **Hub Score** |
| --- | --- | --- | --- | --- | --- |
| **Healthy Control** | | | | | |
| *Oscillibacter sp ER4* | 125 | 6802.0 | 0.00 | 1.00 | 4.22 |
| *Oscillibacter valericigenes* | 106 | 6982.5 | 0.00 | 0.84 | 3.82 |
| *Clostridium innocuum* | 102 | 3942.0 | 0.00 | 0.96 | 3.08 |
| *GGB9699 SGB15216* | 95 | 3863.0 | 0.00 | 0.83 | 2.81 |
| *Enterocloster bolteae* | 74 | 4371.0 | 0.00 | 0.72 | 2.54 |
| *Megasphaera micronuciformis* | 34 | 8465.5 | 0.00 | 0.12 | 2.42 |
| *Oscillospiraceae bacterium Marseille Q3528* | 90 | 2690.0 | 0.00 | 0.81 | 2.39 |
| *Erysipelatoclostridium ramosum* | 95 | 1693.5 | 0.00 | 0.91 | 2.32 |
| *GGB2998 SGB3988* | 85 | 3311.5 | 0.00 | 0.65 | 2.31 |
| *Oscillospiraceae bacterium* | 71 | 3830.0 | 0.00 | 0.55 | 2.14 |
| *Faecalimonas umbilicata* | 82 | 1594.5 | 0.00 | 0.85 | 2.04 |
| *Flavonifractor plautii* | 84 | 1245.5 | 0.00 | 0.83 | 1.95 |
| *Blautia hansenii* | 80 | 1300.0 | 0.00 | 0.82 | 1.90 |
| *Blautia producta* | 62 | 2334.5 | 0.00 | 0.67 | 1.77 |
| *Ruminococcus gnavus* | 72 | 853.0 | 0.00 | 0.81 | 1.65 |
| *Blautia caecimuris* | 63 | 1309.0 | 0.00 | 0.73 | 1.57 |
| *Oscillibacter sp MSJ 31* | 66 | 1882.5 | 0.00 | 0.52 | 1.51 |
| *Lachnospiraceae bacterium CLA AA H244* | 64 | 1029.0 | 0.00 | 0.72 | 1.49 |
| *Lachnospiraceae bacterium AM48 27BH* | 62 | 791.5 | 0.00 | 0.69 | 1.37 |
| *Clostridiaceae bacterium Marseille Q4143* | 59 | 627.0 | 0.00 | 0.67 | 1.26 |
| *Tyzzerella nexilis* | 55 | 894.0 | 0.00 | 0.65 | 1.25 |
| *Clostridiaceae bacterium AF18 31LB* | 58 | 742.5 | 0.00 | 0.63 | 1.23 |
| *Anaerostipes caccae* | 52 | 1045.5 | 0.00 | 0.60 | 1.19 |
| *GGB9345 SGB14311* | 30 | 3874.5 | 0.00 | 0.10 | 1.08 |
| *GGB9708 SGB15234* | 51 | 1582.0 | 0.00 | 0.39 | 1.08 |
| *Coprococcus catus* | 50 | 774.0 | 0.00 | 0.58 | 1.07 |
| *GGB55957 SGB77276* | 54 | 565.5 | 0.00 | 0.57 | 1.06 |
| *Faecalibacterium SGB15346* | 50 | 643.5 | 0.00 | 0.59 | 1.04 |
| *Simiaoa sunii* | 53 | 326.5 | 0.00 | 0.62 | 1.04 |
| *Vescimonas coprocola* | 49 | 730.5 | 0.00 | 0.55 | 1.01 |
| *GGB3619 SGB4894* | 49 | 1181.0 | 0.00 | 0.42 | 0.97 |
| *Blautia obeum* | 46 | 1509.0 | 0.00 | 0.37 | 0.96 |
| *Clostridiaceae unclassified SGB4771* | 51 | 177.0 | 0.00 | 0.60 | 0.94 |
| *Coprococcus eutactus* | 47 | 707.0 | 0.00 | 0.52 | 0.94 |
| *Faecalicatena fissicatena* | 50 | 352.0 | 0.00 | 0.56 | 0.93 |
| *GGB9730 SGB15291* | 48 | 92.0 | 0.00 | 0.63 | 0.92 |
| *Clostridium sp AT4* | 44 | 945.0 | 0.00 | 0.45 | 0.87 |
| *GGB9758 SGB15368* | 43 | 678.5 | 0.00 | 0.50 | 0.86 |
| *Candidatus Cibionibacter quicibialis* | 45 | 265.5 | 0.00 | 0.57 | 0.85 |
| *GGB9614 SGB15049* | 47 | 89.0 | 0.00 | 0.58 | 0.84 |
| *GGB9602 SGB15031* | 47 | 1194.0 | 0.00 | 0.29 | 0.79 |
| *Dorea formicigenerans* | 41 | 1145.5 | 0.00 | 0.36 | 0.79 |
| *Alistipes communis* | 27 | 2190.0 | 0.00 | 0.27 | 0.78 |
| *Mediterraneibacter glycyrrhizinilyticus* | 42 | 266.0 | 0.00 | 0.53 | 0.76 |
| *Coprococcus comes* | 44 | 194.0 | 0.00 | 0.52 | 0.76 |
| *Clostridium butyricum* | 10 | 3610.0 | 0.00 | 0.10 | 0.75 |
| *Phocaeicola plebeius* | 1 | 0.0 | 3.32 | 0.00 | 0.75 |
| *Phocaeicola coprocola* | 1 | 0.0 | 3.32 | 0.00 | 0.75 |
| *GGB4604 SGB6369* | 1 | 0.0 | 3.31 | 0.00 | 0.74 |
| *GGB4603 SGB6367* | 1 | 0.0 | 3.31 | 0.00 | 0.74 |
| *GGB4642 SGB6422* | 1 | 0.0 | 3.28 | 0.00 | 0.73 |
| *GGB4642 SGB6421* | 1 | 0.0 | 3.28 | 0.00 | 0.73 |
| *Faecalicoccus pleomorphus* | 1 | 0.0 | 3.27 | 0.00 | 0.73 |
| *Enorma massiliensis* | 1 | 0.0 | 3.27 | 0.00 | 0.73 |
| *GGB9707 SGB15229* | 48 | 503.0 | 0.00 | 0.37 | 0.71 |
| *Eggerthella lenta* | 37 | 984.5 | 0.00 | 0.37 | 0.70 |
| *GGB9616 SGB15052* | 41 | 390.0 | 0.00 | 0.45 | 0.69 |
| *Enterocloster aldenensis* | 35 | 815.5 | 0.00 | 0.39 | 0.65 |
| *GGB3490 SGB4664* | 1 | 0.0 | 3.07 | 0.00 | 0.65 |
| *GGB51269 SGB5062* | 1 | 0.0 | 3.07 | 0.00 | 0.65 |
| *Enterocloster clostridioformis* | 38 | 174.0 | 0.00 | 0.49 | 0.64 |
| *Romboutsia timonensis* | 1 | 0.0 | 3.03 | 0.00 | 0.64 |
| *Clostridium SGB6179* | 1 | 0.0 | 3.03 | 0.00 | 0.64 |
| *GGB2983 SGB3965* | 1 | 0.0 | 3.02 | 0.00 | 0.64 |
| *GGB2982 SGB3964* | 1 | 0.0 | 3.02 | 0.00 | 0.64 |
| *Hydrogenoanaerobacterium saccharovorans* | 1 | 0.0 | 2.90 | 0.00 | 0.59 |
| *Hydrogenoanaerobacterium SGB53821* | 1 | 0.0 | 2.90 | 0.00 | 0.59 |
| *GGB4676 SGB6465* | 11 | 3388.0 | 0.00 | 0.00 | 0.58 |
| *Phascolarctobacterium succinatutens* | 1 | 0.0 | 2.84 | 0.00 | 0.57 |
| *Phascolarctobacterium faecium* | 1 | 0.0 | 2.84 | 0.00 | 0.57 |
| *Alistipes shahii* | 29 | 1205.0 | 0.00 | 0.29 | 0.56 |
| *Hungatella hathewayi* | 32 | 741.0 | 0.00 | 0.35 | 0.54 |
| *GGB9747 SGB15356* | 38 | 1073.0 | 0.00 | 0.20 | 0.53 |
| *Clostridium paraputrificum* | 8 | 3297.0 | 0.00 | 0.00 | 0.51 |
| *Clostridium sp AF12 28* | 33 | 279.0 | 0.00 | 0.41 | 0.50 |
| *GGB9534 SGB14937* | 16 | 2490.5 | 0.00 | 0.08 | 0.49 |
| *Wujia chipingensis* | 34 | 153.0 | 0.00 | 0.40 | 0.47 |
| *Bifidobacterium pseudocatenulatum* | 1 | 0.0 | 2.57 | 0.00 | 0.47 |
| *Bifidobacterium longum* | 1 | 0.0 | 2.57 | 0.00 | 0.47 |
| *GGB9342 SGB14306* | 33 | 964.0 | 0.00 | 0.22 | 0.46 |
| *Gemmiger formicilis* | 31 | 48.0 | 0.00 | 0.42 | 0.43 |
| *Peptostreptococcus SGB749* | 20 | 1825.5 | 0.00 | 0.12 | 0.41 |
| *Fusicatenibacter sp CLA AA H277* | 30 | 18.0 | 0.00 | 0.37 | 0.35 |
| *Clostridiales bacterium KLE1615* | 29 | 671.0 | 0.00 | 0.23 | 0.33 |
| *Clostridium symbiosum* | 26 | 481.5 | 0.00 | 0.29 | 0.32 |
| *Blautia glucerasea* | 29 | 113 | 0.00 | 0.34 | 0.32 |
| *Lachnospiraceae bacterium* | 27 | 9 | 0.00 | 0.37 | 0.30 |
| *GGB4277 SGB5832* | 10 | 2177 | 0.00 | 0.06 | 0.30 |
| *Oribacterium sinus* | 23 | 1194 | 0.00 | 0.14 | 0.29 |
| *Colidextribacter sp 210702 DFI 3 9* | 26 | 73 | 0.00 | 0.34 | 0.28 |
| *Eubacterium ramulus* | 30 | 91.5 | 0.00 | 0.29 | 0.27 |
| *GGB3321 SGB4394* | 25 | 560 | 0.00 | 0.24 | 0.27 |
| *GGB9760 SGB15373* | 26 | 59 | 0.00 | 0.34 | 0.26 |
| *Anaerotignum sp MSJ 24* | 21 | 771 | 0.00 | 0.21 | 0.24 |
| *Anaerosacchariphilus sp NSJ 68* | 28 | 170 | 0.00 | 0.27 | 0.23 |
| *Lancefieldella parvula* | 27 | 1227 | 0.00 | 0.04 | 0.23 |
| *GGB9616 SGB15051* | 25 | 604 | 0.00 | 0.19 | 0.21 |
| *Brotolimicola acetigignens* | 28 | 249.5 | 0.00 | 0.23 | 0.21 |
| *GGB51441 SGB71759* | 23 | 31 | 0.00 | 0.33 | 0.20 |
| *Clostridium neonatale* | 21 | 375 | 0.00 | 0.26 | 0.19 |
| **Colorectal Adenoma** | | | | | |
| *Clostridium innocuum* | 106 | 3849.5 | 0.00 | 0.99 | 3.72 |
| *GGB9699 SGB15216* | 81 | 5548.0 | 0.00 | 0.70 | 3.68 |
| *Erysipelatoclostridium ramosum* | 107 | 3020.0 | 0.00 | 1.00 | 3.42 |
| *Ruminococcus gnavus* | 98 | 3482.0 | 0.00 | 0.94 | 3.41 |
| *Enterocloster bolteae* | 85 | 2544.0 | 0.00 | 0.84 | 2.72 |
| *Faecalimonas umbilicata* | 80 | 2538.0 | 0.00 | 0.85 | 2.66 |
| *Oscillibacter sp ER4* | 86 | 2289.5 | 0.00 | 0.84 | 2.63 |
| *Oscillibacter valericigenes* | 69 | 3175.0 | 0.00 | 0.61 | 2.45 |
| *GGB9708 SGB15234* | 52 | 2562.0 | 0.00 | 0.44 | 1.76 |
| *Enterocloster aldenensis* | 58 | 1740.0 | 0.00 | 0.62 | 1.74 |
| *Oscillibacter sp MSJ 31* | 51 | 2633.0 | 0.00 | 0.41 | 1.73 |
| *Blautia hansenii* | 65 | 741.0 | 0.00 | 0.76 | 1.63 |
| *Oscillospiraceae bacterium Marseille Q3528* | 59 | 1433.5 | 0.00 | 0.61 | 1.62 |
| *Flavonifractor plautii* | 51 | 1598.5 | 0.00 | 0.62 | 1.59 |
| *Hungatella hathewayi* | 51 | 1651.5 | 0.00 | 0.58 | 1.57 |
| *Blautia caecimuris* | 65 | 483.0 | 0.00 | 0.79 | 1.56 |
| *Coprococcus eutactus* | 52 | 1371.0 | 0.00 | 0.54 | 1.42 |
| *Lachnospiraceae bacterium AM48 27BH* | 54 | 1118.0 | 0.00 | 0.60 | 1.41 |
| *GGB55957 SGB77276* | 49 | 998.0 | 0.00 | 0.61 | 1.31 |
| *Tyzzerella nexilis* | 57 | 370.0 | 0.00 | 0.71 | 1.31 |
| *Clostridium symbiosum* | 42 | 1210.0 | 0.00 | 0.53 | 1.19 |
| *Anaerostipes caccae* | 48 | 606.0 | 0.00 | 0.63 | 1.17 |
| *Clostridiaceae bacterium AF18 31LB* | 47 | 918.0 | 0.00 | 0.51 | 1.13 |
| *Blautia producta* | 49 | 308.5 | 0.00 | 0.67 | 1.11 |
| *Clostridium sp AT4* | 42 | 1127.5 | 0.00 | 0.47 | 1.09 |
| *Clostridiaceae bacterium Marseille Q4143* | 50 | 441.0 | 0.00 | 0.57 | 1.05 |
| *GGB2998 SGB3988* | 41 | 971.0 | 0.00 | 0.42 | 0.95 |
| *Lachnospiraceae bacterium CLA AA H244* | 41 | 435.0 | 0.00 | 0.54 | 0.88 |
| *Coprococcus catus* | 36 | 701.0 | 0.00 | 0.45 | 0.81 |
| *Mediterraneibacter glycyrrhizinilyticus* | 38 | 467.0 | 0.00 | 0.50 | 0.80 |
| *Eggerthella lenta* | 36 | 586.0 | 0.00 | 0.46 | 0.78 |
| *Blautia obeum* | 35 | 1082.0 | 0.00 | 0.29 | 0.74 |
| *GGB9614 SGB15049* | 39 | 305.0 | 0.00 | 0.49 | 0.74 |
| *Mogibacterium diversum* | 32 | 1491.5 | 0.00 | 0.17 | 0.72 |
| *Candidatus Cibionibacter quicibialis* | 39 | 90.0 | 0.00 | 0.51 | 0.68 |
| *Fusobacterium ulcerans* | 37 | 148.0 | 0.00 | 0.50 | 0.67 |
| *Simiaoa sunii* | 37 | 269.0 | 0.00 | 0.46 | 0.66 |
| *GGB9707 SGB15229* | 30 | 1346.0 | 0.00 | 0.19 | 0.65 |
| *GGB9342 SGB14306* | 24 | 1748.0 | 0.00 | 0.12 | 0.63 |
| *GGB3740 SGB5077* | 1 | 0.0 | 3.33 | 0.00 | 0.63 |
| *GGB3740 SGB5076* | 1 | 0.0 | 3.33 | 0.00 | 0.63 |
| *Provencibacterium massiliense* | 1 | 0.0 | 3.27 | 0.00 | 0.60 |
| *Massiliimalia massiliensis* | 1 | 0.0 | 3.27 | 0.00 | 0.60 |
| *GGB3490 SGB4664* | 1 | 0.0 | 3.26 | 0.00 | 0.60 |
| *GGB51269 SGB5062* | 1 | 0.0 | 3.26 | 0.00 | 0.60 |
| *Escherichia coli* | 31 | 438.0 | 0.00 | 0.41 | 0.58 |
| *GGB9730 SGB15291* | 35 | 68.0 | 0.00 | 0.47 | 0.57 |
| *GGB4604 SGB6369* | 1 | 0.0 | 3.14 | 0.00 | 0.56 |
| *GGB4603 SGB6367* | 1 | 0.0 | 3.14 | 0.00 | 0.56 |
| *Faecalibacterium SGB15346* | 35 | 19.0 | 0.00 | 0.47 | 0.55 |
| *Granulicatella adiacens* | 37 | 713.0 | 0.00 | 0.23 | 0.55 |
| *Vescimonas coprocola* | 35 | 234.0 | 0.00 | 0.40 | 0.55 |
| *Coprococcus comes* | 34 | 227.0 | 0.00 | 0.41 | 0.55 |
| *Segatella sinensis* | 1 | 0.0 | 3.06 | 0.00 | 0.53 |
| *Victivallis vadensis* | 1 | 0.0 | 3.06 | 0.00 | 0.53 |
| *Segatella copri* | 1 | 0.0 | 3.06 | 0.00 | 0.53 |
| *GGB6518 SGB9205* | 1 | 0.0 | 3.06 | 0.00 | 0.53 |
| *Streptococcus cristatus* | 38 | 1068.0 | 0.00 | 0.10 | 0.53 |
| *GGB9602 SGB15031* | 27 | 1296.0 | 0.00 | 0.13 | 0.51 |
| *Phocaeicola plebeius* | 1 | 0.0 | 2.98 | 0.00 | 0.50 |
| *Phocaeicola coprocola* | 1 | 0.0 | 2.98 | 0.00 | 0.50 |
| *Fusobacterium nucleatum* | 33 | 1233.0 | 0.00 | 0.08 | 0.50 |
| *Alistipes communis* | 23 | 1114.0 | 0.00 | 0.22 | 0.49 |
| *Lachnospiraceae bacterium* | 33 | 128.0 | 0.00 | 0.40 | 0.48 |
| *Phocaeicola vulgatus* | 30 | 300.0 | 0.00 | 0.38 | 0.48 |
| *Clostridium paraputrificum* | 21 | 1285.0 | 0.00 | 0.16 | 0.46 |
| *Streptococcus mitis* | 24 | 1405.0 | 0.00 | 0.08 | 0.45 |
| *Megasphaera micronuciformis* | 29 | 1066.0 | 0.00 | 0.12 | 0.43 |
| *GGB9747 SGB15356* | 37 | 619.0 | 0.00 | 0.17 | 0.43 |
| *GGB2653 SGB3574* | 1 | 0.0 | 2.74 | 0.00 | 0.42 |
| *GGB2652 SGB3573* | 1 | 0.0 | 2.74 | 0.00 | 0.42 |
| *Enterocloster clostridioformis* | 28 | 133.0 | 0.00 | 0.40 | 0.41 |
| *Hydrogenoanaerobacterium saccharovorans* | 1 | 0.0 | 2.70 | 0.00 | 0.41 |
| *Hydrogenoanaerobacterium SGB53821* | 1 | 0.0 | 2.70 | 0.00 | 0.41 |
| *GGB3619 SGB4894* | 26 | 603.0 | 0.00 | 0.27 | 0.40 |
| *Lancefieldella parvula* | 29 | 805.0 | 0.00 | 0.17 | 0.39 |
| *Eubacterium ramulus* | 23 | 919.0 | 0.00 | 0.19 | 0.38 |
| *GGB2983 SGB3965* | 1 | 0.0 | 2.59 | 0.00 | 0.37 |
| *GGB2982 SGB3964* | 1 | 0.0 | 2.59 | 0.00 | 0.37 |
| *GGB33469 SGB15236* | 31 | 901.0 | 0.00 | 0.09 | 0.36 |
| *Roseburia hominis* | 24 | 557.0 | 0.00 | 0.27 | 0.35 |
| *Dorea formicigenerans* | 22 | 746.0 | 0.00 | 0.22 | 0.34 |
| *GGB3883 SGB5266* | 33 | 463.0 | 0.00 | 0.19 | 0.34 |
| *TM7 phylum sp oral taxon 350* | 31 | 233.0 | 0.00 | 0.26 | 0.31 |
| *Bifidobacterium pseudocatenulatum* | 1 | 0.0 | 2.39 | 0.00 | 0.30 |
| *Bifidobacterium longum* | 1 | 0.0 | 2.39 | 0.00 | 0.30 |
| *GGB9758 SGB15368* | 27 | 263.0 | 0.00 | 0.29 | 0.30 |
| *Streptococcus infantis* | 31 | 701.0 | 0.00 | 0.10 | 0.30 |
| *GGB9296 SGB14253* | 17 | 1432.0 | 0.00 | 0.03 | 0.29 |
| *Oscillospiraceae bacterium* | 26 | 337.0 | 0.00 | 0.24 | 0.25 |
| *Actinomyces sp ICM47* | 30 | 442.0 | 0.00 | 0.16 | 0.25 |
| *Lactonifactor longoviformis* | 23 | 54 | 0.00 | 0.34 | 0.23 |
| *Alistipes shahii* | 20 | 730 | 0.00 | 0.16 | 0.23 |
| *Eggerthia catenaformis* | 25 | 403 | 0.00 | 0.21 | 0.23 |
| *Schaalia odontolytica* | 29 | 267 | 0.00 | 0.20 | 0.23 |
| *Clostridiales bacterium KLE1615* | 24 | 559 | 0.00 | 0.14 | 0.19 |
| *Solobacterium SGB6829* | 29 | 55 | 0.00 | 0.24 | 0.18 |
| *Lachnospiraceae bacterium 2 1 46FAA* | 22 | 59 | 0.00 | 0.31 | 0.18 |
| *Faecalicatena fissicatena* | 22 | 145 | 0.00 | 0.28 | 0.17 |
| *GGB9616 SGB15052* | 24 | 106 | 0.00 | 0.26 | 0.16 |

##### Table S6. Comparison of baseline characteristics between the derivation and validation sets.

| **Variable** | **Derivation set (n=1,890)** | **Validation set (n=1,891)** | ***P* value** |
| --- | --- | --- | --- |
| Age, mean (SD) | 60.03 (6.22) | 59.87 (6.28) | 0.418 |
| Female/Male | 942/948 | 928/963 | 0.661 |
| BMI (kg/m^2^), mean (SD) | 24.13 (2.85) | 24.18 (2.92) | 0.600 |
| Family history of CRC in first-degree relatives, n (%) | 182 (9.63) | 194 (10.26) | 0.554 |
| Regular use of probiotics, n (%) | 21 (1.11) | 19 (1.00) | 0.872 |
| Regular use of antibiotics, n (%) | 26 (1.37) | 25 (1.32) | 0.998 |
| Smoking status, n (%) |  |  | 0.192 |
| Never | 1359 (71.90) | 1348 (71.29) |  |
| Ever | 58 (3.07) | 60 (3.17) |  |
| Sometimes | 131 (6.93) | 105 (5.55) |  |
| Every day | 342 (18.10) | 378 (19.99) |  |
| Drinking frequency, n (%) |  |  | 0.693 |
| Never | 1172 (62.01) | 1205 (63.72) |  |
| Once a month or less | 190 (10.05) | 184 (9.73) |  |
| 2–4 times per month | 140 (7.41) | 123 (6.50) |  |
| 2–3 times per week | 88 (4.66) | 94 (4.97) |  |
| 4 or more times per week | 300 (15.87) | 285 (15.07) |  |
| Diabetes mellitus, n (%) | 118 (6.24) | 122 (6.45) | 0.845 |
| Hypertension, n (%) | 426 (22.54) | 442 (23.37) | 0.568 |
| Dietary pattern, n (%) |  |  | 0.848 |
| Cluster A | 735 (38.89) | 751 (39.71) |  |
| Cluster B | 551 (29.15) | 538 (28.45) |  |
| Cluster C | 604 (31.96) | 602 (31.84) |  |
| Colorectal adenoma, n (%) | 523 (27.67) | 533 (28.19) | 0.752 |

##### Table S7. List of the 240 species retained in the general linear model, of the 77 selected in each of the twelve clusters and of 51 selected in T1 or T2 topology. Related to Figure 4.

| **Species** | **Odds ratio** | **95% Confidence interval** | **Cluster** | **Topology group** |
| --- | --- | --- | --- | --- |
| *Acidaminococcus massiliensis* | 1.63 | 1.18 – 2.24 |  |  |
| *Actinomyces bouchesdurhonensis* | 1.66 | 1.04 – 2.61 |  |  |
| *Actinomyces graevenitzii* | 1.56 | 1.12 – 2.15 | 2 |  |
| *Aggregatibacter sp 2125159857* | 1.93 | 1.16 – 3.15 |  |  |
| *Alistipes dispar* | 0.77 | 0.45 – 1.26 |  |  |
| *Allisonella histaminiformans* | 1.13 | 0.91 – 1.41 |  |  |
| *Amedibacillus dolichus* | 1.21 | 0.93 – 1.57 |  |  |
| *Amedibacterium intestinale* | 1.15 | 0.81 – 1.61 |  |  |
| *Anaerofilum hominis* | 1.34 | 0.95 – 1.88 | 5 |  |
| *Anaerosacchariphilus sp NSJ 68* | 0.78 | 0.62 – 0.98 | 7 | T2 |
| *Anaerostipes caccae* | 1.57 | 1.18 – 2.07 | 10 | T1 |
| *Anaerotruncus massiliensis* | 1.25 | 0.76 – 1.99 |  |  |
| *Bacteroides cellulosilyticus* | 1.11 | 0.91 – 1.36 | 3 |  |
| *Bacteroides faecalis* | 1.37 | 0.89 – 2.07 |  |  |
| *Bacteroides fragilis* | 1.14 | 0.93 – 1.40 |  |  |
| *Bacteroides intestinalis* | 1.22 | 1.00 – 1.49 | 3 |  |
| *Bifidobacterium dentium* | 1.15 | 0.91 – 1.46 |  |  |
| *Bittarella massiliensis* | 1.12 | 0.82 – 1.51 |  |  |
| *Blautia caecimuris* | 1.52 | 1.21 – 1.91 | 10 | T1 |
| *Blautia hansenii* | 1.14 | 0.92 – 1.42 | 10 | T1 |
| *Blautia producta* | 1.42 | 1.08 – 1.85 | 10 | T1 |
| *Blautia stercoris* | 0.68 | 0.56 – 0.84 | 8 | T2 |
| *Butyricimonas faecihominis* | 1.11 | 0.87 – 1.42 |  |  |
| *Butyricimonas synergistica* | 1.14 | 0.88 – 1.46 |  |  |
| *Campylobacter concisus* | 1.18 | 0.87 – 1.60 |  |  |
| *Campylobacter SGB19337* | 1.76 | 1.14 – 2.71 |  |  |
| *Candidatus Nanoperiomorbus periodonticus* | 1.22 | 0.85 – 1.72 |  |  |
| *Catenibacterium tridentinum* | 1.85 | 1.23 – 2.76 |  |  |
| *Christensenella minuta* | 1.14 | 0.83 – 1.53 |  |  |
| *Citrobacter freundii* | 1.24 | 0.92 – 1.67 |  |  |
| *Cloacibacillus porcorum* | 1.34 | 0.91 – 1.93 |  |  |
| *Clostridia bacterium* | 1.30 | 0.92 – 1.83 |  |  |
| *Clostridiaceae bacterium CLA AA H274* | 0.78 | 0.50 – 1.20 |  |  |
| *Clostridiaceae bacterium Marseille Q4143* | 0.76 | 0.62 – 0.93 | 7 | T2 |
| *Clostridiaceae bacterium Marseille Q4145* | 0.80 | 0.63 – 1.00 | 7 | T2 |
| *Clostridiaceae bacterium Marseille Q4149* | 1.11 | 0.89 – 1.38 | 1 |  |
| *Clostridiaceae unclassified SGB4771* | 0.69 | 0.55 – 0.85 | 7 | T2 |
| *Clostridium cuniculi* | 1.11 | 0.79 – 1.55 |  |  |
| *Clostridium disporicum* | 1.33 | 0.85 – 2.04 |  |  |
| *Clostridium innocuum* | 1.22 | 1.00 – 1.49 | 10 | T1 |
| *Clostridium methylpentosum* | 1.42 | 0.97 – 2.06 |  |  |
| *Clostridium paraputrificum* | 1.16 | 0.85 – 1.57 |  |  |
| *Clostridium scindens* | 1.30 | 1.04 – 1.62 |  |  |
| *Clostridium SGB4751* | 0.77 | 0.62 – 0.95 | 8 | T2 |
| *Clostridium SGB48024* | 0.74 | 0.48 – 1.09 |  |  |
| *Clostridium sp AF12 28* | 0.76 | 0.62 – 0.94 | 7 | T2 |
| *Clostridium sp AF27 2AA* | 0.75 | 0.61 – 0.92 |  |  |
| *Clostridium sp AF32 12BH* | 0.67 | 0.54 – 0.82 | 8 | T2 |
| *Clostridium sp AM29 11AC* | 1.20 | 0.80 – 1.76 |  |  |
| *Clostridium sp AT4* | 1.20 | 0.98 – 1.47 | 2 |  |
| *Clostridium sp Marseille P3244* | 0.74 | 0.48 – 1.11 |  |  |
| *Clostridium sp NSJ 6* | 1.32 | 0.82 – 2.09 |  |  |
| *Clostridium spiroforme* | 1.28 | 0.92 – 1.76 |  |  |
| *Clostridium symbiosum* | 1.22 | 0.99 – 1.49 | 9 | T1 |
| *Coprobacillus cateniformis* | 1.43 | 1.13 – 1.79 |  |  |
| *Coprobacter secundus* | 0.78 | 0.59 – 1.02 |  |  |
| *Coprococcus catus* | 0.79 | 0.65 – 0.97 | 7 | T2 |
| *Dialister hominis* | 1.19 | 0.93 – 1.51 |  |  |
| *Dielma fastidiosa* | 1.22 | 0.94 – 1.57 |  |  |
| *Diplocloster modestus* | 0.78 | 0.49 – 1.20 |  |  |
| *Dorea longicatena* | 1.11 | 0.91 – 1.36 | 7 | T2 |
| *Eggerthia catenaformis* | 1.40 | 0.88 – 2.17 |  |  |
| *Emergencia timonensis* | 1.30 | 0.97 – 1.74 |  |  |
| *Enterobacter asburiae* | 1.71 | 1.22 – 2.39 |  |  |
| *Enterobacter hormaechei* | 1.17 | 0.89 – 1.52 | 12 | T1 |
| *Enterobacter kobei* | 1.47 | 1.12 – 1.93 | 12 | T1 |
| *Enterobacter roggenkampii* | 1.40 | 1.08 – 1.83 | 12 | T1 |
| *Enterocloster asparagiformis* | 1.18 | 0.96 – 1.45 |  |  |
| *Enterocloster bolteae* | 1.17 | 0.96 – 1.44 |  |  |
| *Enterocloster clostridioformis* | 1.21 | 0.95 – 1.55 |  |  |
| *Erysipelatoclostridium ramosum* | 1.32 | 1.08 – 1.62 | 10 | T1 |
| *Escherichia coli* | 1.29 | 1.05 – 1.58 |  |  |
| *Eubacterium hominis* | 1.30 | 1.05 – 1.61 |  |  |
| *Eubacterium siraeum* | 1.20 | 0.94 – 1.53 | 3 |  |
| *Eubacterium sp AF34 35BH* | 0.79 | 0.64 – 0.98 | 2 |  |
| *Eubacterium sulci* | 1.41 | 1.03 – 1.92 | 2 |  |
| *Faecalibacillus faecis* | 0.66 | 0.49 – 0.90 |  |  |
| *Faecalibacterium SGB15346* | 0.74 | 0.61 – 0.91 | 7 | T2 |
| *Faecalicatena fissicatena* | 0.65 | 0.53 – 0.80 | 8 | T2 |
| *Faecalimonas umbilicata* | 1.11 | 0.89 – 1.38 | 10 | T1 |
| *Frisingicoccus caecimuris* | 1.18 | 0.79 – 1.74 |  |  |
| *Fructilactobacillus sanfranciscensis* | 0.62 | 0.37 – 0.97 |  |  |
| *Fumia xinanensis* | 0.72 | 0.50 – 1.03 |  |  |
| *Fusobacterium mortiferum* | 1.11 | 0.87 – 1.40 |  |  |
| *Fusobacterium ulcerans* | 1.29 | 0.96 – 1.73 |  |  |
| *Fusobacterium varium* | 1.18 | 0.82 – 1.69 |  |  |
| *GGB12785 SGB19823* | 1.56 | 1.01 – 2.39 |  |  |
| *GGB13489 SGB15224* | 0.72 | 0.44 – 1.14 |  |  |
| *GGB13493 SGB15238* | 0.71 | 0.51 – 0.96 | 4 | T2 |
| *GGB1495 SGB2071* | 0.75 | 0.55 – 1.02 |  |  |
| *GGB1663 SGB2279* | 1.16 | 0.86 – 1.55 |  |  |
| *GGB2652 SGB3573* | 0.76 | 0.51 – 1.10 |  |  |
| *GGB3005 SGB3996* | 1.11 | 0.78 – 1.56 |  |  |
| *GGB3109 SGB4121* | 1.15 | 0.93 – 1.40 |  |  |
| *GGB3175 SGB4191* | 0.78 | 0.57 – 1.06 |  |  |
| *GGB3278 SGB4328* | 0.77 | 0.57 – 1.04 |  |  |
| *GGB3288 SGB4342* | 0.78 | 0.57 – 1.06 | 4 | T2 |
| *GGB3304 SGB4365* | 1.31 | 0.91 – 1.87 |  |  |
| *GGB3345 SGB4425* | 0.76 | 0.60 – 0.97 |  |  |
| *GGB34228 SGB72916* | 1.12 | 0.86 – 1.45 | 4 | T2 |
| *GGB3433 SGB4573* | 1.41 | 0.84 – 2.32 |  |  |
| *GGB3486 SGB4658* | 0.78 | 0.56 – 1.06 |  |  |
| *GGB3490 SGB4664* | 0.65 | 0.49 – 0.85 |  |  |
| *GGB3588 SGB4808* | 0.71 | 0.46 – 1.06 |  |  |
| *GGB3730 SGB5060* | 1.41 | 0.92 – 2.14 |  |  |
| *GGB3738 SGB5072* | 0.61 | 0.42 – 0.85 |  |  |
| *GGB3818 SGB5183* | 1.21 | 0.92 – 1.59 |  |  |
| *GGB3819 SGB5184* | 1.12 | 0.82 – 1.51 |  |  |
| *GGB3828 SGB5197* | 0.66 | 0.53 – 0.81 | 8 | T2 |
| *GGB3883 SGB5266* | 1.61 | 0.97 – 2.63 |  |  |
| *GGB41458 SGB58520* | 0.74 | 0.59 – 0.94 | 2 |  |
| *GGB4578 SGB6328* | 0.71 | 0.50 – 0.99 |  |  |
| *GGB4585 SGB6340* | 0.79 | 0.55 – 1.10 |  |  |
| *GGB4604 SGB6369* | 0.71 | 0.46 – 1.06 |  |  |
| *GGB4682 SGB6472* | 0.77 | 0.46 – 1.23 |  |  |
| *GGB4683 SGB6473* | 0.74 | 0.56 – 0.96 | 2 |  |
| *GGB47687 SGB2286* | 1.18 | 0.80 – 1.72 |  |  |
| *GGB51269 SGB5061* | 0.80 | 0.55 – 1.14 |  |  |
| *GGB51269 SGB5062* | 0.76 | 0.55 – 1.02 |  |  |
| *GGB51884 SGB49168* | 0.72 | 0.53 – 0.96 | 6 | T2 |
| *GGB58233 SGB79883* | 1.25 | 0.98 – 1.59 |  |  |
| *GGB58485 SGB80143* | 0.74 | 0.57 – 0.97 | 4 | T2 |
| *GGB6574 SGB9290* | 0.75 | 0.52 – 1.07 |  |  |
| *GGB6649 SGB9391* | 0.80 | 0.54 – 1.16 |  |  |
| *GGB9062 SGB13981* | 0.76 | 0.55 – 1.04 |  |  |
| *GGB9063 SGB13982* | 0.73 | 0.54 – 0.97 | 5 |  |
| *GGB9237 SGB14179* | 1.18 | 0.94 – 1.49 | 1 |  |
| *GGB9252 SGB14196* | 0.65 | 0.40 – 1.01 |  |  |
| *GGB9290 SGB14247* | 1.76 | 1.21 – 2.54 | 1 |  |
| *GGB9302 SGB14263* | 0.61 | 0.42 – 0.88 | 4 | T2 |
| *GGB9342 SGB14306* | 0.76 | 0.58 – 0.98 | 4 | T2 |
| *GGB9491 SGB14888* | 0.80 | 0.64 – 0.99 |  |  |
| *GGB9501 SGB14898* | 0.59 | 0.37 – 0.90 |  |  |
| *GGB9574 SGB14987* | 1.12 | 0.74 – 1.66 |  |  |
| *GGB9575 SGB14988* | 1.10 | 0.70 – 1.71 |  |  |
| *GGB9593 SGB15015* | 0.75 | 0.57 – 0.98 | 4 | T2 |
| *GGB9595 SGB15019* | 0.65 | 0.46 – 0.89 | 4 | T2 |
| *GGB9602 SGB15031* | 0.76 | 0.59 – 0.98 | 4 | T2 |
| *GGB9608 SGB15041* | 1.14 | 0.79 – 1.64 | 1 |  |
| *GGB9614 SGB15049* | 0.75 | 0.61 – 0.91 | 7 | T2 |
| *GGB9616 SGB15052* | 0.74 | 0.60 – 0.92 | 7 | T2 |
| *GGB9634 SGB15093* | 0.78 | 0.57 – 1.05 | 6 | T2 |
| *GGB9667 SGB15164* | 0.79 | 0.56 – 1.10 |  |  |
| *GGB9712 SGB15244* | 1.25 | 0.95 – 1.65 | 1 |  |
| *GGB9715 SGB15266* | 1.26 | 0.80 – 1.94 |  |  |
| *GGB9715 SGB15267* | 0.76 | 0.58 – 0.99 |  |  |
| *GGB9719 SGB15272* | 1.17 | 0.86 – 1.58 |  |  |
| *GGB9730 SGB15291* | 0.79 | 0.64 – 0.96 | 7 | T2 |
| *GGB9758 SGB15368* | 0.76 | 0.61 – 0.94 | 4 | T2 |
| *GGB9759 SGB15370* | 0.77 | 0.58 – 1.02 | 6 | T2 |
| *GGB9760 SGB15373* | 0.75 | 0.61 – 0.93 | 4 | T2 |
| *GGB9767 SGB15385* | 0.65 | 0.42 – 1.00 |  |  |
| *GGB9813 SGB15447* | 1.39 | 1.05 – 1.83 |  |  |
| *GGB9818 SGB15459* | 1.29 | 0.83 – 1.96 |  |  |
| *Granulicatella adiacens* | 1.63 | 1.02 – 2.56 |  |  |
| *Granulicatella SGB8255* | 1.26 | 1.02 – 1.54 |  |  |
| *Haemophilus haemolyticus* | 1.35 | 0.88 – 2.04 |  |  |
| *Holdemanella biformis* | 1.42 | 1.04 – 1.94 |  |  |
| *Holdemania filiformis* | 1.12 | 0.92 – 1.37 | 3 |  |
| *Hungatella hathewayi* | 1.11 | 0.91 – 1.36 | 9 | T1 |
| *Hydrogeniiclostridium mannosilyticum* | 1.12 | 0.85 – 1.47 |  |  |
| *Jilunia laotingensis* | 1.29 | 0.99 – 1.67 |  |  |
| *Klebsiella aerogenes* | 1.18 | 0.87 – 1.60 |  |  |
| *Klebsiella michiganensis* | 1.20 | 0.77 – 1.85 |  |  |
| *Klebsiella pneumoniae* | 1.17 | 0.95 – 1.43 |  |  |
| *Klebsiella quasipneumoniae* | 1.60 | 1.21 – 2.10 |  |  |
| *Klebsiella variicola* | 1.26 | 0.97 – 1.62 | 12 | T1 |
| *Lachnospira pectinoschiza* | 1.12 | 0.92 – 1.38 |  |  |
| *Lachnospira sp NSJ 43* | 0.70 | 0.57 – 0.86 | 8 | T2 |
| *Lachnospiraceae bacterium AM48 27BH* | 0.79 | 0.65 – 0.97 | 7 | T2 |
| *Lachnospiraceae bacterium CLA AA H244* | 0.65 | 0.53 – 0.79 | 7 | T2 |
| *Lachnospiraceae bacterium NSJ 143* | 1.46 | 1.12 – 1.88 |  |  |
| *Lactobacillus crispatus* | 1.24 | 0.85 – 1.77 |  |  |
| *Lactobacillus gasseri* | 1.47 | 0.98 – 2.16 |  |  |
| *Lancefieldella parvula* | 1.52 | 1.05 – 2.16 |  |  |
| *Lawsonibacter asaccharolyticus* | 0.78 | 0.63 – 0.95 | 3 |  |
| *Lawsonibacter hominis* | 0.74 | 0.53 – 1.01 | 5 |  |
| *Longicatena caecimuris* | 1.12 | 0.86 – 1.46 |  |  |
| *Massiliimalia massiliensis* | 1.16 | 0.84 – 1.57 | 5 |  |
| *Massiliimalia timonensis* | 1.13 | 0.87 – 1.46 |  |  |
| *Mediterraneibacter glycyrrhizinilyticus* | 1.39 | 1.13 – 1.70 |  |  |
| *Mediterraneibacter massiliensis* | 1.19 | 0.79 – 1.77 |  |  |
| *Megamonas funiformis* | 1.17 | 0.96 – 1.43 |  |  |
| *Megasphaera sp NM10* | 1.25 | 0.93 – 1.68 |  |  |
| *Mesosutterella multiformis* | 1.16 | 0.92 – 1.46 |  |  |
| *Methanobrevibacter smithii* | 1.23 | 0.88 – 1.72 | 1 |  |
| *Mogibacterium diversum* | 1.45 | 0.96 – 2.17 |  |  |
| *Negativibacillus massiliensis* | 1.23 | 0.97 – 1.55 |  |  |
| *Oscillibacter hominis* | 0.78 | 0.53 – 1.11 |  |  |
| *Oscillibacter sp ER4* | 0.74 | 0.60 – 0.91 | 7 | T2 |
| *Oscillospiraceae bacterium* | 0.72 | 0.57 – 0.89 | 4 | T2 |
| *Oscillospiraceae bacterium Marseille Q3528* | 0.74 | 0.60 – 0.91 | 7 | T2 |
| *Oxalobacter formigenes* | 1.43 | 1.01 – 1.99 |  |  |
| *Parabacteroides faecis* | 1.33 | 0.97 – 1.81 |  |  |
| *Parabacteroides goldsteinii* | 1.31 | 1.07 – 1.61 |  |  |
| *Parabacteroides gordonii* | 1.14 | 0.89 – 1.47 |  |  |
| *Parabacteroides sp N37* | 1.31 | 0.91 – 1.86 |  |  |
| *Parabacteroides sp TM07 1AC* | 1.55 | 1.10 – 2.16 |  |  |
| *Phascolarctobacterium sp ET69* | 0.78 | 0.51 – 1.17 |  |  |
| *Phocaeicola coprocola* | 1.15 | 0.94 – 1.40 |  |  |
| *Phocaeicola massiliensis* | 1.21 | 0.99 – 1.48 |  |  |
| *Phocaeicola vulgatus* | 1.24 | 1.01 – 1.52 |  |  |
| *Pseudoflavonifractor phocaeensis* | 1.13 | 0.79 – 1.60 |  |  |
| *Pseudoflavonifractor sp AF19 9AC* | 0.72 | 0.50 – 1.03 |  |  |
| *Pyramidobacter porci* | 1.19 | 0.73 – 1.87 |  |  |
| *Raoultella ornithinolytica* | 1.19 | 0.84 – 1.65 |  |  |
| *Raoultibacter timonensis* | 1.62 | 1.04 – 2.48 |  |  |
| *Romboutsia timonensis* | 1.13 | 0.93 – 1.39 |  |  |
| *Roseburia hominis* | 0.73 | 0.60 – 0.90 | 11 |  |
| *Roseburia inulinivorans* | 1.13 | 0.93 – 1.39 | 11 |  |
| *Roseburia lenta* | 0.76 | 0.61 – 0.94 | 8 | T2 |
| *Roseburia sp AF02 12* | 0.80 | 0.64 – 1.00 | 11 |  |
| *Roseburia sp AM59 24XD* | 0.72 | 0.46 – 1.09 |  |  |
| *Roseburia sp BX1005* | 0.67 | 0.47 – 0.94 |  |  |
| *Ruminococcus gnavus* | 1.39 | 1.14 – 1.71 |  |  |
| *Ruminococcus lactaris* | 0.80 | 0.64 – 0.98 | 2 |  |
| *Ruminococcus sp AF41 9* | 0.61 | 0.46 – 0.80 |  |  |
| *Schaalia SGB17168* | 1.41 | 0.94 – 2.07 |  |  |
| *Segatella brunsvicensis* | 1.46 | 0.98 – 2.15 |  |  |
| *Segatella hominis* | 1.13 | 0.84 – 1.51 |  |  |
| *Segatella sinensis* | 1.19 | 0.97 – 1.45 |  |  |
| *Segatella sinica* | 1.46 | 0.99 – 2.13 |  |  |
| *Solobacterium SGB6829* | 1.20 | 0.74 – 1.91 |  |  |
| *Streptococcus cristatus* | 1.24 | 0.94 – 1.63 |  |  |
| *Streptococcus gallolyticus* | 1.15 | 0.75 – 1.73 |  |  |
| *Streptococcus mitis* | 1.35 | 1.10 – 1.66 |  |  |
| *Streptococcus parasanguinis* | 1.25 | 1.02 – 1.53 |  |  |
| *Streptococcus rubneri* | 1.11 | 0.87 – 1.40 |  |  |
| *Streptococcus thermophilus* | 0.75 | 0.53 – 1.04 |  |  |
| *Streptococcus vestibularis* | 1.23 | 0.86 – 1.72 |  |  |
| *Sutterella wadsworthensis* | 1.17 | 0.91 – 1.49 |  |  |
| *TM7 phylum sp oral taxon 352* | 1.23 | 1.01 – 1.51 |  |  |
| *Turicibacter sanguinis* | 1.19 | 0.92 – 1.53 |  |  |
| *Tyzzerella nexilis* | 1.34 | 1.09 – 1.65 | 10 | T1 |
| *Tyzzerella SGB4587* | 1.16 | 0.86 – 1.54 |  |  |
| *Victivallis lenta* | 0.71 | 0.51 – 0.98 |  |  |
| *Victivallis vadensis* | 0.73 | 0.51 – 1.03 |  |  |
| *Wansuia hejianensis* | 0.75 | 0.55 – 1.01 |  |  |
| *Weissella confusa* | 1.11 | 0.74 – 1.63 |  |  |
| *Youxingia wuxianensis* | 1.11 | 0.78 – 1.57 | 5 |  |

CI, confidence interval; OR, odds ratio.

##### Table S8. List of the bacteria in neoplasia-related microbial association patterns. Related to Figure 4.

| **Species** | **Cluster** | **Topology** |
| --- | --- | --- |
| *Anaerosacchariphilus sp NSJ 68* | 7 | T2 |
| *Blautia stercoris* | 8 | T2 |
| *Clostridiaceae bacterium Marseille Q4143* | 7 | T2 |
| *Clostridiaceae bacterium Marseille Q4145* | 7 | T2 |
| *Clostridiaceae unclassified SGB4771* | 7 | T2 |
| *Clostridium SGB4751* | 8 | T2 |
| *Clostridium sp AF12 28* | 7 | T2 |
| *Clostridium sp AF32 12BH* | 8 | T2 |
| *Coprococcus catus* | 7 | T2 |
| *Dorea longicatena* | 7 | T2 |
| *Faecalibacterium SGB15346* | 7 | T2 |
| *Faecalicatena fissicatena* | 8 | T2 |
| *GGB13493 SGB15238* | 4 | T2 |
| *GGB3288 SGB4342* | 4 | T2 |
| *GGB34228 SGB72916* | 4 | T2 |
| *GGB3828 SGB5197* | 8 | T2 |
| *GGB51884 SGB49168* | 6 | T2 |
| *GGB58485 SGB80143* | 4 | T2 |
| *GGB9302 SGB14263* | 4 | T2 |
| *GGB9342 SGB14306* | 4 | T2 |
| *GGB9593 SGB15015* | 4 | T2 |
| *GGB9595 SGB15019* | 4 | T2 |
| *GGB9602 SGB15031* | 4 | T2 |
| *GGB9614 SGB15049* | 7 | T2 |
| *GGB9616 SGB15052* | 7 | T2 |
| *GGB9634 SGB15093* | 6 | T2 |
| *GGB9730 SGB15291* | 7 | T2 |
| *GGB9758 SGB15368* | 4 | T2 |
| *GGB9759 SGB15370* | 6 | T2 |
| *GGB9760 SGB15373* | 4 | T2 |
| *Lachnospira sp NSJ 43* | 8 | T2 |
| *Lachnospiraceae bacterium AM48 27BH* | 7 | T2 |
| *Lachnospiraceae bacterium CLA AA H244* | 7 | T2 |
| *Oscillibacter sp ER4* | 7 | T2 |
| *Oscillospiraceae bacterium* | 4 | T2 |
| *Oscillospiraceae bacterium Marseille Q3528* | 7 | T2 |
| *Roseburia lenta* | 8 | T2 |
| *Anaerostipes caccae* | 10 | T1 |
| *Blautia caecimuris* | 10 | T1 |
| *Blautia hansenii* | 10 | T1 |
| *Blautia producta* | 10 | T1 |
| *Clostridium innocuum* | 10 | T1 |
| *Clostridium symbiosum* | 9 | T1 |
| *Enterobacter hormaechei* | 12 | T1 |
| *Enterobacter kobei* | 12 | T1 |
| *Enterobacter roggenkampii* | 12 | T1 |
| *Erysipelatoclostridium ramosum* | 10 | T1 |
| *Faecalimonas umbilicata* | 10 | T1 |
| *Hungatella hathewayi* | 9 | T1 |
| *Klebsiella variicola* | 12 | T1 |
| *Tyzzerella nexilis* | 10 | T1 |

##### Table S9. Subject metadata for the eight public cohorts.

| NCBI accession | Accession ID | Sex | Age | BMI | Country | Study condition |
| --- | --- | --- | --- | --- | --- | --- |
| ERR1018185 | PRJEB10878 | M | 64 | 23.1 | CHN | colorectal cancer |
| ERR1018186 | PRJEB10878 | M | 73 | 23.8 | CHN | colorectal cancer |
| ERR1018187 | PRJEB10878 | F | 67 | 24.7 | CHN | colorectal cancer |
| ERR1018188 | PRJEB10878 | M | 56 | 25.6 | CHN | colorectal cancer |
| ERR1018189 | PRJEB10878 | F | 59 | 20.7 | CHN | colorectal cancer |
| ERR1018190 | PRJEB10878 | M | 61 | 25.2 | CHN | colorectal cancer |
| ERR1018191 | PRJEB10878 | M | 60 | 23.2 | CHN | colorectal cancer |
| ERR1018192 | PRJEB10878 | F | 73 | 17.7 | CHN | colorectal cancer |
| ERR1018193 | PRJEB10878 | M | 62 | 21.7 | CHN | control |
| ERR1018194 | PRJEB10878 | F | 72 | 26.3 | CHN | colorectal cancer |
| ERR1018195 | PRJEB10878 | M | 65 | 20.7 | CHN | control |
| ERR1018196 | PRJEB10878 | M | 65 | 24.8 | CHN | colorectal cancer |
| ERR1018197 | PRJEB10878 | M | 71 | 25.8 | CHN | colorectal cancer |
| ERR1018198 | PRJEB10878 | M | 50 | 25.2 | CHN | colorectal cancer |
| ERR1018199 | PRJEB10878 | M | 55 | 22.2 | CHN | colorectal cancer |
| ERR1018200 | PRJEB10878 | M | 77 | 21.7 | CHN | colorectal cancer |
| ERR1018201 | PRJEB10878 | F | 45 | 23 | CHN | colorectal cancer |
| ERR1018202 | PRJEB10878 | F | 89 | 23.7 | CHN | colorectal cancer |
| ERR1018203 | PRJEB10878 | F | 58 | 21.6 | CHN | control |
| ERR1018204 | PRJEB10878 | F | 58 | 23.9 | CHN | colorectal cancer |
| ERR1018205 | PRJEB10878 | M | 68 | 21.7 | CHN | control |
| ERR1018206 | PRJEB10878 | F | 64 | 29.1 | CHN | colorectal cancer |
| ERR1018207 | PRJEB10878 | F | 75 | 28.7 | CHN | colorectal cancer |
| ERR1018208 | PRJEB10878 | M | 73 | 22.7 | CHN | colorectal cancer |
| ERR1018209 | PRJEB10878 | F | 62 | 21.4 | CHN | control |
| ERR1018210 | PRJEB10878 | M | 80 | 24 | CHN | colorectal cancer |
| ERR1018211 | PRJEB10878 | M | 67 | 23.1 | CHN | colorectal cancer |
| ERR1018212 | PRJEB10878 | F | 61 | 22 | CHN | colorectal cancer |
| ERR1018213 | PRJEB10878 | M | 63 | 24.4 | CHN | colorectal cancer |
| ERR1018214 | PRJEB10878 | F | 51 | 19.6 | CHN | colorectal cancer |
| ERR1018215 | PRJEB10878 | M | 64 | NA | CHN | colorectal cancer |
| ERR1018216 | PRJEB10878 | F | 74 | 28.7 | CHN | colorectal cancer |
| ERR1018217 | PRJEB10878 | M | 68 | 20.4 | CHN | control |
| ERR1018218 | PRJEB10878 | F | 83 | 30.4 | CHN | colorectal cancer |
| ERR1018219 | PRJEB10878 | M | 70 | 24.6 | CHN | colorectal cancer |
| ERR1018220 | PRJEB10878 | M | 56 | 25.9 | CHN | colorectal cancer |
| ERR1018221 | PRJEB10878 | M | 54 | 17.9 | CHN | colorectal cancer |
| ERR1018222 | PRJEB10878 | M | 74 | 26.8 | CHN | colorectal cancer |
| ERR1018223 | PRJEB10878 | M | 57 | 23.2 | CHN | colorectal cancer |
| ERR1018224 | PRJEB10878 | F | 54 | 29.7 | CHN | colorectal cancer |
| ERR1018225 | PRJEB10878 | M | 62 | 22.7 | CHN | colorectal cancer |
| ERR1018226 | PRJEB10878 | F | 54 | 25.6 | CHN | colorectal cancer |
| ERR1018227 | PRJEB10878 | M | 69 | 29 | CHN | colorectal cancer |
| ERR1018228 | PRJEB10878 | M | 64 | 24.8 | CHN | colorectal cancer |
| ERR1018229 | PRJEB10878 | M | 44 | 22.5 | CHN | colorectal cancer |
| ERR1018230 | PRJEB10878 | M | 60 | 17.3 | CHN | colorectal cancer |
| ERR1018231 | PRJEB10878 | M | 73 | 24.5 | CHN | colorectal cancer |
| ERR1018232 | PRJEB10878 | F | 62 | 32.5 | CHN | control |
| ERR1018233 | PRJEB10878 | M | 58 | 22.3 | CHN | control |
| ERR1018234 | PRJEB10878 | M | 64 | 24.8 | CHN | control |
| ERR1018235 | PRJEB10878 | F | 65 | 35.1 | CHN | control |
| ERR1018236 | PRJEB10878 | M | 62 | 23.5 | CHN | control |
| ERR1018237 | PRJEB10878 | F | 69 | 24.9 | CHN | control |
| ERR1018238 | PRJEB10878 | M | 55 | 26.4 | CHN | control |
| ERR1018239 | PRJEB10878 | F | 64 | 26.4 | CHN | control |
| ERR1018240 | PRJEB10878 | M | 59 | 25.6 | CHN | control |
| ERR1018241 | PRJEB10878 | M | 70 | 21 | CHN | colorectal cancer |
| ERR1018242 | PRJEB10878 | F | 71 | 26.7 | CHN | control |
| ERR1018243 | PRJEB10878 | M | 51 | 21.9 | CHN | control |
| ERR1018244 | PRJEB10878 | M | 50 | 20.5 | CHN | control |
| ERR1018245 | PRJEB10878 | M | 64 | 24.7 | CHN | control |
| ERR1018246 | PRJEB10878 | M | 50 | 23.3 | CHN | control |
| ERR1018247 | PRJEB10878 | F | 66 | 22.4 | CHN | control |
| ERR1018248 | PRJEB10878 | M | 52 | 28.4 | CHN | control |
| ERR1018249 | PRJEB10878 | M | 64 | 25.6 | CHN | control |
| ERR1018250 | PRJEB10878 | M | 58 | 23.1 | CHN | control |
| ERR1018251 | PRJEB10878 | F | 64 | 17.1 | CHN | control |
| ERR1018252 | PRJEB10878 | M | 65 | 22.4 | CHN | control |
| ERR1018253 | PRJEB10878 | M | 53 | 19.7 | CHN | control |
| ERR1018254 | PRJEB10878 | F | 64 | 21.1 | CHN | control |
| ERR1018255 | PRJEB10878 | F | 68 | 24.9 | CHN | control |
| ERR1018256 | PRJEB10878 | M | 53 | 23.4 | CHN | control |
| ERR1018257 | PRJEB10878 | F | 64 | 22.7 | CHN | control |
| ERR1018258 | PRJEB10878 | M | 61 | 21.5 | CHN | control |
| ERR1018259 | PRJEB10878 | F | 62 | 21.1 | CHN | control |
| ERR1018260 | PRJEB10878 | M | 64 | 24.1 | CHN | control |
| ERR1018261 | PRJEB10878 | M | 55 | 22.3 | CHN | control |
| ERR1018262 | PRJEB10878 | F | 64 | 22.6 | CHN | control |
| ERR1018263 | PRJEB10878 | F | 71 | 23 | CHN | control |
| ERR1018264 | PRJEB10878 | F | 64 | 22.2 | CHN | control |
| ERR1018265 | PRJEB10878 | M | 65 | 22.5 | CHN | control |
| ERR1018266 | PRJEB10878 | F | 73 | 24.1 | CHN | control |
| ERR1018267 | PRJEB10878 | M | 54 | 27.7 | CHN | control |
| ERR1018268 | PRJEB10878 | F | 65 | 21.2 | CHN | control |
| ERR1018269 | PRJEB10878 | M | 60 | 22.5 | CHN | control |
| ERR1018270 | PRJEB10878 | M | 58 | 26.3 | CHN | control |
| ERR1018271 | PRJEB10878 | F | 64 | 21.3 | CHN | control |
| ERR1018272 | PRJEB10878 | M | 69 | 23.8 | CHN | control |
| ERR1018273 | PRJEB10878 | F | 65 | 20 | CHN | control |
| ERR1018274 | PRJEB10878 | M | 59 | 23.7 | CHN | control |
| ERR1018275 | PRJEB10878 | F | 70 | 22.2 | CHN | control |
| ERR1018276 | PRJEB10878 | M | 61 | 23.7 | CHN | control |
| ERR1018277 | PRJEB10878 | M | 52 | 24.6 | CHN | control |
| ERR1018278 | PRJEB10878 | M | 58 | 24.5 | CHN | control |
| ERR1018279 | PRJEB10878 | M | 60 | 20 | CHN | colorectal cancer |
| ERR1018280 | PRJEB10878 | M | 77 | 24.5 | CHN | colorectal cancer |
| ERR1018281 | PRJEB10878 | F | 59 | 27 | CHN | colorectal cancer |
| ERR1018282 | PRJEB10878 | F | 71 | 21.2 | CHN | colorectal cancer |
| ERR1018283 | PRJEB10878 | M | 56 | 31.3 | CHN | colorectal cancer |
| ERR1018284 | PRJEB10878 | M | 77 | 22 | CHN | colorectal cancer |
| ERR1018285 | PRJEB10878 | M | 77 | 23.5 | CHN | colorectal cancer |
| ERR1018286 | PRJEB10878 | F | 83 | 22.8 | CHN | colorectal cancer |
| ERR1018287 | PRJEB10878 | F | 79 | 25.8 | CHN | colorectal cancer |
| ERR1018288 | PRJEB10878 | F | 69 | 28.3 | CHN | colorectal cancer |
| ERR1018289 | PRJEB10878 | M | 62 | 23.5 | CHN | colorectal cancer |
| ERR1018290 | PRJEB10878 | M | 78 | 27 | CHN | colorectal cancer |
| ERR1018291 | PRJEB10878 | F | 49 | 22.6 | CHN | colorectal cancer |
| ERR1018292 | PRJEB10878 | M | 81 | 18.2 | CHN | colorectal cancer |
| ERR1018293 | PRJEB10878 | F | 63 | 28 | CHN | colorectal cancer |
| ERR1018294 | PRJEB10878 | M | 59 | 24.5 | CHN | colorectal cancer |
| ERR1018295 | PRJEB10878 | M | 73 | 22.1 | CHN | colorectal cancer |
| ERR1018296 | PRJEB10878 | M | 76 | 21.9 | CHN | colorectal cancer |
| ERR1018297 | PRJEB10878 | F | 74 | 17.9 | CHN | colorectal cancer |
| ERR1018298 | PRJEB10878 | M | 51 | 26.9 | CHN | colorectal cancer |
| ERR1018299 | PRJEB10878 | M | 74 | 24.2 | CHN | colorectal cancer |
| ERR1018300 | PRJEB10878 | M | 77 | 20.2 | CHN | colorectal cancer |
| ERR1018301 | PRJEB10878 | M | 34 | 19.4 | CHN | colorectal cancer |
| ERR1018302 | PRJEB10878 | F | 70 | 26.7 | CHN | colorectal cancer |
| ERR1018303 | PRJEB10878 | F | 83 | 18.9 | CHN | colorectal cancer |
| ERR1018304 | PRJEB10878 | M | 68 | 21.5 | CHN | colorectal cancer |
| ERR1018305 | PRJEB10878 | M | 79 | 26.8 | CHN | colorectal cancer |
| ERR1018306 | PRJEB10878 | F | 64 | 28.3 | CHN | colorectal cancer |
| ERR1018307 | PRJEB10878 | M | 67 | 24.9 | CHN | colorectal cancer |
| ERR1018308 | PRJEB10878 | M | 61 | 23.8 | CHN | control |
| ERR1018309 | PRJEB10878 | M | 62 | 21.5 | CHN | control |
| ERR1018310 | PRJEB10878 | M | 68 | 23.7 | CHN | colorectal cancer |
| ERR1018311 | PRJEB10878 | M | 55 | 25.8 | CHN | colorectal cancer |
| ERR1018312 | PRJEB10878 | M | 63 | 22.5 | CHN | colorectal cancer |
| ERR688505 | ERP008729 | M | 64 | 29.3523545 | AUS | colorectal cancer |
| ERR688506 | ERP008729 | M | 68 | 32 | AUS | control |
| ERR688507 | ERP008729 | F | 60 | 22.1 | AUS | control |
| ERR688508 | ERP008729 | M | 70 | 34.11 | AUS | adenoma |
| ERR688509 | ERP008729 | M | 68 | 23.45 | AUS | control |
| ERR688510 | ERP008729 | M | 66 | 30.3 | AUS | control |
| ERR688511 | ERP008729 | M | 73 | 29.7 | AUS | control |
| ERR688512 | ERP008729 | M | 67 | 27.14 | AUS | adenoma |
| ERR688513 | ERP008729 | M | 73 | 17.99015276 | AUS | colorectal cancer |
| ERR688514 | ERP008729 | F | 67 | 30 | AUS | control |
| ERR688515 | ERP008729 | M | 65 | 28.02768166 | AUS | colorectal cancer |
| ERR688516 | ERP008729 | F | 74 | 28.3 | AUS | control |
| ERR688517 | ERP008729 | F | 65 | 23.30668005 | AUS | colorectal cancer |
| ERR688519 | ERP008729 | M | 62 | 26.88 | AUS | adenoma |
| ERR688520 | ERP008729 | M | 65 | 29.06 | AUS | control |
| ERR688521 | ERP008729 | M | 67 | 29.40292024 | AUS | colorectal cancer |
| ERR688522 | ERP008729 | F | 69 | 24.13 | AUS | adenoma |
| ERR688523 | ERP008729 | M | 67 | 22.79 | AUS | control |
| ERR688524 | ERP008729 | M | 62 | 30.1 | AUS | adenoma |
| ERR688525 | ERP008729 | M | 82 | 30.47052154 | AUS | colorectal cancer |
| ERR688526 | ERP008729 | M | 63 | 34.08 | AUS | adenoma |
| ERR688527 | ERP008729 | M | 84 | 27.28174603 | AUS | colorectal cancer |
| ERR688528 | ERP008729 | F | 68 | 21.7 | AUS | control |
| ERR688529 | ERP008729 | M | 63 | 30.9 | AUS | control |
| ERR688530 | ERP008729 | M | 68 | 22.2 | AUS | control |
| ERR688531 | ERP008729 | M | 62 | 35.43 | AUS | adenoma |
| ERR688532 | ERP008729 | M | 72 | 29.03 | AUS | control |
| ERR688533 | ERP008729 | M | 71 | 29.04 | AUS | control |
| ERR688534 | ERP008729 | F | 86 | 20.504934 | AUS | colorectal cancer |
| ERR688535 | ERP008729 | M | 63 | 30.4 | AUS | control |
| ERR688536 | ERP008729 | F | 68 | 34.01 | AUS | adenoma |
| ERR688537 | ERP008729 | M | 72 | 31.8 | AUS | control |
| ERR688538 | ERP008729 | F | 68 | 24.84 | AUS | adenoma |
| ERR688539 | ERP008729 | F | 69 | 30 | AUS | control |
| ERR688540 | ERP008729 | M | 71 | 27.04 | AUS | adenoma |
| ERR688541 | ERP008729 | F | 84 | 31.21748179 | AUS | colorectal cancer |
| ERR688542 | ERP008729 | M | 63 | 30.77 | AUS | adenoma |
| ERR688543 | ERP008729 | M | 67 | 23.14 | AUS | control |
| ERR688544 | ERP008729 | F | 66 | 21.7 | AUS | control |
| ERR688545 | ERP008729 | F | 72 | 27.43 | AUS | adenoma |
| ERR688546 | ERP008729 | F | 78 | 31.24 | AUS | adenoma |
| ERR688547 | ERP008729 | M | 60 | 26.64359862 | AUS | colorectal cancer |
| ERR688548 | ERP008729 | M | 68 | 32.24939532 | AUS | colorectal cancer |
| ERR688549 | ERP008729 | F | 69 | 24.4 | AUS | adenoma |
| ERR688550 | ERP008729 | M | 64 | 29.3 | AUS | control |
| ERR688551 | ERP008729 | M | 65 | 28.9 | AUS | control |
| ERR688552 | ERP008729 | F | 70 | 31 | AUS | control |
| ERR688553 | ERP008729 | F | 84 | 27.76709812 | AUS | colorectal cancer |
| ERR688554 | ERP008729 | F | 74 | 28.5 | AUS | control |
| ERR688555 | ERP008729 | M | 77 | 25.7 | AUS | adenoma |
| ERR688557 | ERP008729 | M | 66 | 29.98 | AUS | control |
| ERR688558 | ERP008729 | M | 74 | 26.36560303 | AUS | colorectal cancer |
| ERR688559 | ERP008729 | F | 72 | 28.7 | AUS | control |
| ERR688560 | ERP008729 | F | 84 | 24.7 | AUS | adenoma |
| ERR688561 | ERP008729 | M | 66 | 30.02 | AUS | control |
| ERR688562 | ERP008729 | F | 69 | 28.3 | AUS | control |
| ERR688563 | ERP008729 | F | 69 | 22.4 | AUS | control |
| ERR688564 | ERP008729 | M | 70 | 31.25 | AUS | control |
| ERR688565 | ERP008729 | M | 66 | 34.04 | AUS | control |
| ERR688566 | ERP008729 | F | 73 | 32 | AUS | control |
| ERR688567 | ERP008729 | M | 54 | 27.33564014 | AUS | colorectal cancer |
| ERR688568 | ERP008729 | F | 55 | 23.33546614 | AUS | colorectal cancer |
| ERR688569 | ERP008729 | F | 73 | 29.75206612 | AUS | colorectal cancer |
| ERR688570 | ERP008729 | F | 64 | 24.08822312 | AUS | colorectal cancer |
| ERR688571 | ERP008729 | M | 63 | 25.88057064 | AUS | colorectal cancer |
| ERR688572 | ERP008729 | M | 72 | 28.40816327 | AUS | colorectal cancer |
| ERR688573 | ERP008729 | M | 46 | 30.86419753 | AUS | colorectal cancer |
| ERR688574 | ERP008729 | M | 73 | 24.81632653 | AUS | colorectal cancer |
| ERR688575 | ERP008729 | F | 74 | 29.17488494 | AUS | colorectal cancer |
| ERR688576 | ERP008729 | M | 58 | 27.75748722 | AUS | colorectal cancer |
| ERR688577 | ERP008729 | F | 56 | 20.06920415 | AUS | colorectal cancer |
| ERR688578 | ERP008729 | F | 72 | 26.6727633 | AUS | colorectal cancer |
| ERR688579 | ERP008729 | F | 52 | 27.14158239 | AUS | colorectal cancer |
| ERR688580 | ERP008729 | M | 71 | 29.73704434 | AUS | colorectal cancer |
| ERR688581 | ERP008729 | M | 74 | 24.33747972 | AUS | colorectal cancer |
| ERR688582 | ERP008729 | F | 60 | 30.078125 | AUS | colorectal cancer |
| ERR688583 | ERP008729 | M | 43 | 22.67995241 | AUS | colorectal cancer |
| ERR688584 | ERP008729 | M | 56 | 29.41 | AUS | adenoma |
| ERR688585 | ERP008729 | F | 83 | 21.94 | AUS | adenoma |
| ERR688586 | ERP008729 | M | 67 | 33.23 | AUS | adenoma |
| ERR688587 | ERP008729 | F | 60 | 30 | AUS | adenoma |
| ERR688588 | ERP008729 | M | 67 | 28.34 | AUS | adenoma |
| ERR688589 | ERP008729 | M | 69 | 21.46 | AUS | adenoma |
| ERR688590 | ERP008729 | F | 71 | 29 | AUS | control |
| ERR688591 | ERP008729 | M | 64 | 29.38 | AUS | control |
| ERR688592 | ERP008729 | F | 64 | 20.52 | AUS | adenoma |
| ERR688593 | ERP008729 | M | 64 | 22.78 | AUS | control |
| ERR688594 | ERP008729 | F | 68 | 22.1 | AUS | control |
| ERR688595 | ERP008729 | M | 63 | 34.14 | AUS | control |
| ERR688596 | ERP008729 | M | 70 | 31.6 | AUS | control |
| ERR688597 | ERP008729 | F | 61 | 22.8 | AUS | adenoma |
| ERR688598 | ERP008729 | F | 74 | 28.5 | AUS | control |
| ERR688599 | ERP008729 | M | 63 | 30.4 | AUS | control |
| ERR688600 | ERP008729 | F | 72 | 30 | AUS | control |
| ERR688601 | ERP008729 | F | 62 | 32.8 | AUS | adenoma |
| ERR688602 | ERP008729 | M | 63 | 27.4 | AUS | adenoma |
| ERR688603 | ERP008729 | F | 71 | 29 | AUS | control |
| ERR688604 | ERP008729 | F | 52 | 32.8 | AUS | adenoma |
| ERR688605 | ERP008729 | M | 65 | 31.22 | AUS | control |
| ERR688606 | ERP008729 | F | 68 | 22.4 | AUS | control |
| ERR688607 | ERP008729 | M | 72 | 31.8 | AUS | control |
| ERR688608 | ERP008729 | M | 65 | 22.03 | AUS | control |
| ERR688609 | ERP008729 | M | 64 | 29.3 | AUS | adenoma |
| ERR688610 | ERP008729 | F | 70 | 22.67 | AUS | control |
| ERR688611 | ERP008729 | F | 78 | 24 | AUS | adenoma |
| ERR688612 | ERP008729 | F | 75 | 31 | AUS | control |
| ERR688613 | ERP008729 | M | 71 | 24.8 | AUS | adenoma |
| ERR688614 | ERP008729 | M | 70 | 23.67 | AUS | control |
| ERR688615 | ERP008729 | M | 58 | 21.7 | AUS | adenoma |
| ERR688616 | ERP008729 | M | 63 | 31.17 | AUS | control |
| ERR688617 | ERP008729 | F | 70 | 22.8 | AUS | control |
| ERR688618 | ERP008729 | F | 47 | 25.78125 | AUS | colorectal cancer |
| ERR688619 | ERP008729 | F | 67 | 22.58 | AUS | control |
| ERR688620 | ERP008729 | M | 80 | 26.47 | AUS | adenoma |
| ERR688621 | ERP008729 | F | 75 | 30.12 | AUS | adenoma |
| ERR688622 | ERP008729 | F | 62 | 22.1 | AUS | control |
| ERR688623 | ERP008729 | M | 48 | 25.61 | AUS | adenoma |
| ERR688624 | ERP008729 | M | 68 | 29.8 | AUS | control |
| ERR688625 | ERP008729 | F | 56 | 22.8 | AUS | adenoma |
| ERR688626 | ERP008729 | F | 70 | 25.2 | AUS | adenoma |
| ERR688627 | ERP008729 | M | 62 | 24.3 | AUS | adenoma |
| ERR688628 | ERP008729 | F | 63 | 30.47 | AUS | adenoma |
| ERR688629 | ERP008729 | M | 77 | 37.56 | AUS | adenoma |
| ERR688630 | ERP008729 | F | 66 | 22.65 | AUS | adenoma |
| ERR688631 | ERP008729 | F | 70 | 29.38 | AUS | adenoma |
| ERR688632 | ERP008729 | F | 74 | 24.13 | AUS | adenoma |
| ERR688633 | ERP008729 | M | 58 | 27.02 | AUS | adenoma |
| ERR688634 | ERP008729 | M | 77 | 29.41176471 | AUS | colorectal cancer |
| ERR688635 | ERP008729 | M | 52 | 25.72755521 | AUS | colorectal cancer |
| ERR688636 | ERP008729 | F | 72 | 24.21875 | AUS | colorectal cancer |
| ERR688637 | ERP008729 | M | 79 | 26.29757785 | AUS | colorectal cancer |
| ERR688638 | ERP008729 | M | 77 | 19.15708812 | AUS | colorectal cancer |
| ERR688639 | ERP008729 | M | 64 | 29.0687733 | AUS | colorectal cancer |
| ERR688640 | ERP008729 | F | 72 | 27.12031558 | AUS | colorectal cancer |
| ERR688641 | ERP008729 | M | 69 | 29.9382716 | AUS | colorectal cancer |
| ERR688642 | ERP008729 | M | 66 | 22.5981405 | AUS | colorectal cancer |
| ERR688643 | ERP008729 | F | 72 | 23.828125 | AUS | colorectal cancer |
| ERR688644 | ERP008729 | M | 65 | 29.74062741 | AUS | colorectal cancer |
| ERR688645 | ERP008729 | F | 59 | 29.2 | AUS | adenoma |
| ERR688646 | ERP008729 | M | 46 | 23.15 | AUS | control |
| ERR688647 | ERP008729 | M | 63 | 33.56401384 | AUS | colorectal cancer |
| ERR688648 | ERP008729 | M | 46 | 28.74 | AUS | control |
| ERR688649 | ERP008729 | F | 45 | 22.14532872 | AUS | colorectal cancer |
| ERR688650 | ERP008729 | M | 72 | 24.33747972 | AUS | colorectal cancer |
| ERR688651 | ERP008729 | F | 77 | 23.49523687 | AUS | colorectal cancer |
| ERR710424 | ERP008729 | M | 63 | 25.64891762 | AUS | adenoma |
| ERR710425 | ERP008729 | F | 56 | 43.57993197 | AUS | adenoma |
| ERR710426 | ERP008729 | M | 55 | 27.76342975 | AUS | control |
| ERR710427 | ERP008729 | M | 70 | 28.71972318 | AUS | control |
| ERR710428 | ERP008729 | F | 73 | 26.5625 | AUS | control |
| ERR710429 | ERP008729 | M | 68 | 23.52941176 | AUS | control |
| ERR710430 | ERP008729 | M | 78 | 31.22130395 | AUS | control |
| ERR710431 | ERP008729 | F | 68 | 27.54820937 | AUS | adenoma |
| ERR710432 | ERP008729 | F | 43 | 22.64737696 | AUS | control |
| SRR8865600 | PRJNA531273 | NA | 41 | 19.21637827 | IND | colorectal cancer |
| SRR8865596 | PRJNA531273 | M | 59 | 21.40102364 | IND | colorectal cancer |
| SRR8865598 | PRJNA531273 | M | 62 | 20.07733492 | IND | colorectal cancer |
| SRR8865599 | PRJNA531273 | F | 65 | 21.00532419 | IND | colorectal cancer |
| SRR8865601 | PRJNA531273 | M | 65 | 20.32225287 | IND | colorectal cancer |
| SRR8865594 | PRJNA531273 | M | 48 | 18.687452 | IND | colorectal cancer |
| SRR8865595 | PRJNA531273 | M | 56 | 20.44913742 | IND | colorectal cancer |
| SRR8865597 | PRJNA531273 | M | 55 | 19.13655669 | IND | colorectal cancer |
| SRR8865592 | PRJNA531273 | M | 49 | 20.82644628 | IND | colorectal cancer |
| SRR8865593 | PRJNA531273 | M | 72 | 18.21832243 | IND | colorectal cancer |
| SRR8865584 | PRJNA531273 | F | 75 | 21.20844875 | IND | colorectal cancer |
| SRR8865585 | PRJNA531273 | F | 58 | 22.66666667 | IND | colorectal cancer |
| SRR8865586 | PRJNA531273 | M | 59 | 19.10009183 | IND | colorectal cancer |
| SRR8865587 | PRJNA531273 | F | 66 | 20.504934 | IND | colorectal cancer |
| SRR8865588 | PRJNA531273 | F | 56 | 22.22222222 | IND | colorectal cancer |
| SRR5898912 | PRJNA531273 | F | 23 | 22.30814991 | IND | control |
| SRR5898911 | PRJNA531273 | F | 25 | 21.26654064 | IND | control |
| SRR5898910 | PRJNA531273 | M | 24 | 26.03749399 | IND | control |
| SRR5898909 | PRJNA531273 | F | 22 | 23.23345618 | IND | control |
| SRR5898908 | PRJNA531273 | F | 29 | 22.03172568 | IND | control |
| SRR5898920 | PRJNA531273 | F | 24 | 23.25592719 | IND | control |
| SRR5898921 | PRJNA531273 | F | 23 | 19.22768787 | IND | control |
| SRR5898922 | PRJNA531273 | F | 26 | 22.03172568 | IND | control |
| SRR5898923 | PRJNA531273 | F | 26 | 20.82093992 | IND | control |
| SRR5898924 | PRJNA531273 | M | 25 | 21.71806609 | IND | control |
| SRR5898925 | PRJNA531273 | F | 26 | 20.82093992 | IND | control |
| SRR5898926 | PRJNA531273 | M | 26 | 23.33546614 | IND | control |
| SRR5899017 | PRJNA531273 | F | 50 | 27.05515088 | IND | control |
| SRR5898966 | PRJNA531273 | F | 64 | 36.42323803 | IND | control |
| SRR5898984 | PRJNA531273 | F | 62 | 22.94811574 | IND | control |
| SRR5898985 | PRJNA531273 | M | 57 | 17.94108149 | IND | control |
| SRR5898986 | PRJNA531273 | F | 57 | 20.83252961 | IND | control |
| SRR5898987 | PRJNA531273 | M | 71 | 19.21667085 | IND | control |
| SRR5898980 | PRJNA531273 | F | 52 | 23.00415033 | IND | control |
| SRR5898981 | PRJNA531273 | M | 62 | 20.81806008 | IND | control |
| SRR5898982 | PRJNA531273 | F | 50 | 17.92821543 | IND | control |
| SRR5898983 | PRJNA531273 | M | 58 | 19.21667085 | IND | control |
| SRR5898978 | PRJNA531273 | M | 61 | 19.48772644 | IND | control |
| SRR5898979 | PRJNA531273 | F | 58 | 19.93693029 | IND | control |
| SRR5898954 | PRJNA531273 | F | 53 | 26.83817539 | IND | control |
| SRR5898950 | PRJNA531273 | F | 50 | 25.12911004 | IND | control |
| SRR5898948 | PRJNA531273 | F | 58 | 25.6543724 | IND | control |
| SRR5899005 | PRJNA531273 | M | 30 | 24.97704316 | IND | control |
| SRR5899007 | PRJNA531273 | M | 26 | 24.67550027 | IND | control |
| SRR5899006 | PRJNA531273 | M | 25 | 24.8015873 | IND | control |
| SRR8865589 | PRJNA531273 | F | 47 | 23.80540166 | IND | colorectal cancer |
| SRR8865590 | PRJNA531273 | F | 53 | 22.9719382 | IND | colorectal cancer |
| SRR8865591 | PRJNA531273 | M | 61 | 19.46740129 | IND | colorectal cancer |
| SRR8865582 | PRJNA531273 | M | 57 | 21.69663983 | IND | colorectal cancer |
| SRR8865583 | PRJNA531273 | M | 64 | 20.07733492 | IND | colorectal cancer |
| SRR8865577 | PRJNA531273 | M | 71 | 18.06616734 | IND | colorectal cancer |
| SRR8865576 | PRJNA531273 | F | 58 | 21.49028551 | IND | colorectal cancer |
| SRR8865579 | PRJNA531273 | M | 54 | 18.96192742 | IND | colorectal cancer |
| SRR8865578 | PRJNA531273 | F | 62 | 20.02535977 | IND | colorectal cancer |
| SRR8865573 | PRJNA531273 | F | 66 | 18.73048907 | IND | colorectal cancer |
| SRR8865572 | PRJNA531273 | F | 59 | 20.34279778 | IND | colorectal cancer |
| SRR8865575 | PRJNA531273 | M | 60 | 18.32991985 | IND | colorectal cancer |
| SRR8865574 | PRJNA531273 | M | 58 | 19.921875 | IND | colorectal cancer |
| SRR8865581 | PRJNA531273 | M | 71 | 20.02884153 | IND | colorectal cancer |
| SRR8865580 | PRJNA531273 | M | 67 | 17.68978885 | IND | colorectal cancer |
| SRR6915091 | PRJNA447983 | M | 77 | 23 | ITA | colorectal cancer |
| SRR6915092 | PRJNA447983 | F | 60 | 20 | ITA | control |
| SRR6915093 | PRJNA447983 | M | 69 | 27 | ITA | colorectal cancer |
| SRR6915094 | PRJNA447983 | M | 82 | 28 | ITA | colorectal cancer |
| SRR6915095 | PRJNA447983 | M | 84 | 22 | ITA | colorectal cancer |
| SRR6915096 | PRJNA447983 | M | 60 | 24 | ITA | adenoma |
| SRR6915097 | PRJNA447983 | F | 80 | 32 | ITA | control |
| SRR6915098 | PRJNA447983 | M | 74 | 23 | ITA | colorectal cancer |
| SRR6915099 | PRJNA447983 | M | 63 | 25 | ITA | colorectal cancer |
| SRR6915100 | PRJNA447983 | M | 80 | 24 | ITA | control |
| SRR6915101 | PRJNA447983 | F | 84 | 23 | ITA | colorectal cancer |
| SRR6915102 | PRJNA447983 | M | 61 | 23 | ITA | colorectal cancer |
| SRR6915103 | PRJNA447983 | NA | NA | NA | ITA | colorectal cancer |
| SRR6915104 | PRJNA447983 | NA | NA | NA | ITA | colorectal cancer |
| SRR6915105 | PRJNA447983 | NA | NA | NA | ITA | colorectal cancer |
| SRR6915106 | PRJNA447983 | NA | NA | NA | ITA | control |
| SRR6915107 | PRJNA447983 | NA | NA | NA | ITA | colorectal cancer |
| SRR6915108 | PRJNA447983 | NA | NA | NA | ITA | control |
| SRR6915109 | PRJNA447983 | NA | NA | NA | ITA | colorectal cancer |
| SRR6915110 | PRJNA447983 | NA | NA | NA | ITA | control |
| SRR6915111 | PRJNA447983 | NA | NA | NA | ITA | control |
| SRR6915112 | PRJNA447983 | NA | NA | NA | ITA | control |
| SRR6915113 | PRJNA447983 | F | 77 | NA | ITA | control |
| SRR6915114 | PRJNA447983 | NA | NA | NA | ITA | control |
| SRR6915115 | PRJNA447983 | F | 83 | 19 | ITA | colorectal cancer |
| SRR6915116 | PRJNA447983 | F | 70 | 25 | ITA | control |
| SRR6915117 | PRJNA447983 | NA | NA | NA | ITA | control |
| SRR6915118 | PRJNA447983 | M | 67 | 26 | ITA | adenoma |
| SRR6915119 | PRJNA447983 | M | 66 | 24 | ITA | colorectal cancer |
| SRR6915120 | PRJNA447983 | M | 64 | 23 | ITA | colorectal cancer |
| SRR6915121 | PRJNA447983 | M | 66 | 34 | ITA | control |
| SRR6915122 | PRJNA447983 | M | 64 | 26 | ITA | control |
| SRR6915123 | PRJNA447983 | M | 78 | 26 | ITA | colorectal cancer |
| SRR6915124 | PRJNA447983 | M | 77 | 36 | ITA | adenoma |
| SRR6915125 | PRJNA447983 | NA | NA | NA | ITA | colorectal cancer |
| SRR6915126 | PRJNA447983 | NA | NA | NA | ITA | control |
| SRR6915127 | PRJNA447983 | NA | NA | NA | ITA | colorectal cancer |
| SRR6915128 | PRJNA447983 | NA | NA | NA | ITA | colorectal cancer |
| SRR6915129 | PRJNA447983 | NA | NA | NA | ITA | colorectal cancer |
| SRR6915130 | PRJNA447983 | NA | NA | NA | ITA | colorectal cancer |
| SRR6915131 | PRJNA447983 | NA | NA | NA | ITA | control |
| SRR6915132 | PRJNA447983 | NA | NA | NA | ITA | colorectal cancer |
| SRR6915133 | PRJNA447983 | NA | NA | NA | ITA | colorectal cancer |
| SRR6915134 | PRJNA447983 | NA | NA | NA | ITA | control |
| SRR6915135 | PRJNA447983 | NA | NA | NA | ITA | colorectal cancer |
| SRR6915136 | PRJNA447983 | NA | NA | NA | ITA | colorectal cancer |
| SRR6915137 | PRJNA447983 | F | 69 | 29 | ITA | colorectal cancer |
| SRR6915138 | PRJNA447983 | F | 58 | 25 | ITA | adenoma |
| SRR6915139 | PRJNA447983 | M | 58 | 23 | ITA | adenoma |
| SRR6915140 | PRJNA447983 | M | 77 | 24 | ITA | adenoma |
| SRR6915141 | PRJNA447983 | M | 58 | 26 | ITA | adenoma |
| SRR6915142 | PRJNA447983 | F | 63 | 21 | ITA | adenoma |
| SRR6915143 | PRJNA447983 | M | 79 | 22 | ITA | adenoma |
| SRR6915144 | PRJNA447983 | F | 68 | 22 | ITA | control |
| SRR6915145 | PRJNA447983 | M | 60 | 22 | ITA | control |
| SRR6915146 | PRJNA447983 | F | 53 | 23 | ITA | adenoma |
| SRR6915147 | PRJNA447983 | NA | NA | NA | ITA | colorectal cancer |
| SRR6915148 | PRJNA447983 | NA | NA | NA | ITA | control |
| SRR6915149 | PRJNA447983 | NA | NA | NA | ITA | control |
| SRR6915150 | PRJNA447983 | NA | NA | NA | ITA | colorectal cancer |
| SRR6915151 | PRJNA447983 | NA | NA | NA | ITA | colorectal cancer |
| SRR6915152 | PRJNA447983 | NA | NA | NA | ITA | colorectal cancer |
| SRR6915153 | PRJNA447983 | NA | NA | NA | ITA | colorectal cancer |
| SRR6915154 | PRJNA447983 | NA | NA | NA | ITA | colorectal cancer |
| SRR6915155 | PRJNA447983 | NA | NA | NA | ITA | colorectal cancer |
| SRR6915156 | PRJNA447983 | NA | NA | NA | ITA | colorectal cancer |
| SRR6915157 | PRJNA447983 | M | 70 | 28 | ITA | adenoma |
| SRR6915158 | PRJNA447983 | F | 71 | 30 | ITA | colorectal cancer |
| SRR6915159 | PRJNA447983 | M | 58 | 25 | ITA | colorectal cancer |
| SRR6915160 | PRJNA447983 | F | 67 | 21 | ITA | colorectal cancer |
| SRR6915161 | PRJNA447983 | M | 61 | 29 | ITA | adenoma |
| SRR6915162 | PRJNA447983 | M | 49 | 21 | ITA | adenoma |
| SRR6915163 | PRJNA447983 | M | 57 | 29 | ITA | adenoma |
| SRR6915164 | PRJNA447983 | M | 58 | 24 | ITA | control |
| SRR6915165 | PRJNA447983 | F | 61 | NA | ITA | control |
| SRR6915166 | PRJNA447983 | M | 70 | 24 | ITA | colorectal cancer |
| SRR6915167 | PRJNA447983 | NA | NA | NA | ITA | control |
| SRR6915168 | PRJNA447983 | NA | NA | NA | ITA | control |
| SRR6915169 | PRJNA447983 | NA | NA | NA | ITA | control |
| SRR6915170 | PRJNA447983 | NA | NA | NA | ITA | control |
| SRR6915171 | PRJNA447983 | NA | NA | NA | ITA | control |
| SRR6915172 | PRJNA447983 | NA | NA | NA | ITA | control |
| SRR6915173 | PRJNA447983 | NA | NA | NA | ITA | control |
| SRR6915174 | PRJNA447983 | NA | NA | NA | ITA | control |
| SRR6915175 | PRJNA447983 | NA | NA | NA | ITA | control |
| SRR6915176 | PRJNA447983 | NA | NA | NA | ITA | control |
| SRR6915177 | PRJNA447983 | NA | NA | NA | ITA | colorectal cancer |
| SRR6915178 | PRJNA447983 | NA | NA | NA | ITA | colorectal cancer |
| SRR6915179 | PRJNA447983 | NA | NA | NA | ITA | colorectal cancer |
| SRR6915180 | PRJNA447983 | NA | NA | NA | ITA | colorectal cancer |
| SRR6915181 | PRJNA447983 | NA | NA | NA | ITA | colorectal cancer |
| SRR6915182 | PRJNA447983 | NA | NA | NA | ITA | colorectal cancer |
| SRR6915183 | PRJNA447983 | NA | NA | NA | ITA | colorectal cancer |
| SRR6915184 | PRJNA447983 | NA | NA | NA | ITA | colorectal cancer |
| SRR6915185 | PRJNA447983 | NA | NA | NA | ITA | colorectal cancer |
| SRR6915186 | PRJNA447983 | NA | NA | NA | ITA | control |
| SRR6915187 | PRJNA447983 | M | 64 | 25 | ITA | control |
| SRR6915188 | PRJNA447983 | M | 60 | 22 | ITA | colorectal cancer |
| SRR6915189 | PRJNA447983 | F | 81 | 28 | ITA | control |
| SRR6915190 | PRJNA447983 | M | 78 | 24 | ITA | colorectal cancer |
| SRR6915191 | PRJNA447983 | M | 69 | 24 | ITA | control |
| SRR6915192 | PRJNA447983 | M | 64 | 27 | ITA | control |
| SRR6915193 | PRJNA447983 | M | 69 | 30 | ITA | control |
| SRR6915194 | PRJNA447983 | M | 71 | 24 | ITA | control |
| SRR6915195 | PRJNA447983 | NA | NA | NA | ITA | colorectal cancer |
| SRR6915196 | PRJNA447983 | NA | NA | NA | ITA | control |
| SRR6915197 | PRJNA447983 | NA | NA | NA | ITA | control |
| SRR6915198 | PRJNA447983 | NA | NA | NA | ITA | control |
| SRR6915199 | PRJNA447983 | NA | NA | NA | ITA | control |
| SRR6915200 | PRJNA447983 | NA | NA | NA | ITA | control |
| SRR6915201 | PRJNA447983 | F | 83 | 24 | ITA | colorectal cancer |
| SRR6915202 | PRJNA447983 | M | 61 | 22 | ITA | control |
| SRR6915203 | PRJNA447983 | M | 67 | NA | ITA | adenoma |
| SRR6915204 | PRJNA447983 | F | 53 | 21 | ITA | adenoma |
| SRR6915205 | PRJNA447983 | F | 56 | 30 | ITA | adenoma |
| SRR6915206 | PRJNA447983 | M | 59 | 26 | ITA | control |
| SRR6915207 | PRJNA447983 | F | 63 | 28 | ITA | control |
| SRR6915208 | PRJNA447983 | M | 76 | 26 | ITA | colorectal cancer |
| SRR6915209 | PRJNA447983 | M | 57 | 30 | ITA | colorectal cancer |
| SRR6915210 | PRJNA447983 | M | 74 | 25 | ITA | colorectal cancer |
| SRR6915211 | PRJNA447983 | M | 64 | 30 | ITA | colorectal cancer |
| SRR6915212 | PRJNA447983 | F | 64 | 25 | ITA | adenoma |
| SRR6915213 | PRJNA447983 | M | 78 | 30 | ITA | colorectal cancer |
| SRR6915214 | PRJNA447983 | M | 50 | 26 | ITA | adenoma |
| SRR6915215 | PRJNA447983 | M | 57 | 29 | ITA | adenoma |
| SRR6915216 | PRJNA447983 | M | 59 | 22 | ITA | adenoma |
| SRR6915217 | PRJNA447983 | F | 67 | 24 | ITA | control |
| SRR6915218 | PRJNA447983 | M | 63 | 37 | ITA | colorectal cancer |
| SRR6915219 | PRJNA447983 | M | 77 | 22 | ITA | colorectal cancer |
| SRR6915220 | PRJNA447983 | M | 67 | 35 | ITA | colorectal cancer |
| SRR6915221 | PRJNA447983 | F | 70 | 21 | ITA | adenoma |
| SRR6915222 | PRJNA447983 | F | 70 | 20 | ITA | control |
| SRR6915223 | PRJNA447983 | M | 75 | NA | ITA | colorectal cancer |
| SRR6915224 | PRJNA447983 | F | 77 | 20 | ITA | adenoma |
| SRR6915225 | PRJNA447983 | F | 77 | 25 | ITA | control |
| SRR6915226 | PRJNA447983 | F | 68 | 30 | ITA | adenoma |
| SRR6915227 | PRJNA447983 | M | 71 | 25 | ITA | control |
| SRR6915228 | PRJNA447983 | F | 73 | 31 | ITA | adenoma |
| SRR6915229 | PRJNA447983 | F | 62 | 29 | ITA | adenoma |
| SRR6915230 | PRJNA447983 | M | 55 | 19 | ITA | adenoma |
| ERR1293497 | PRJEB12449 | M | 65 | 23.5 | USA | control |
| ERR1293878 | PRJEB12449 | M | 65 | 29.6 | USA | control |
| ERR1293874 | PRJEB12449 | M | 51 | 25.8 | USA | colorectal cancer |
| ERR1293545 | PRJEB12449 | M | 53 | 25 | USA | colorectal cancer |
| ERR1293810 | PRJEB12449 | F | 84 | NA | USA | colorectal cancer |
| ERR1293914 | PRJEB12449 | F | 64 | 35.7 | USA | colorectal cancer |
| ERR1293738 | PRJEB12449 | M | 53 | 27.9 | USA | colorectal cancer |
| ERR1293814 | PRJEB12449 | F | 63 | 16.8 | USA | control |
| ERR1293618 | PRJEB12449 | M | 69 | 24.1 | USA | control |
| ERR1293565 | PRJEB12449 | M | 42 | 22 | USA | colorectal cancer |
| ERR1293758 | PRJEB12449 | F | 62 | 20.7 | USA | control |
| ERR1293650 | PRJEB12449 | M | 56 | 23 | USA | control |
| ERR1293581 | PRJEB12449 | M | 70 | 25.1 | USA | colorectal cancer |
| ERR1293702 | PRJEB12449 | F | 60 | 20.5 | USA | control |
| ERR1293750 | PRJEB12449 | F | 54 | 23.5 | USA | colorectal cancer |
| ERR1293710 | PRJEB12449 | M | 49 | 33.4 | USA | control |
| ERR1293674 | PRJEB12449 | M | 73 | 25.8 | USA | colorectal cancer |
| ERR1293670 | PRJEB12449 | M | 82 | 20.5 | USA | control |
| ERR1293666 | PRJEB12449 | M | 48 | 23.1 | USA | control |
| ERR1293790 | PRJEB12449 | M | 71 | 24.7 | USA | colorectal cancer |
| ERR1293862 | PRJEB12449 | M | 72 | 23.1 | USA | control |
| ERR1293818 | PRJEB12449 | M | 63 | 31.6 | USA | control |
| ERR1293509 | PRJEB12449 | M | 48 | 26.4 | USA | control |
| ERR1293846 | PRJEB12449 | F | 76 | 22.1 | USA | control |
| ERR1293794 | PRJEB12449 | F | 55 | 23.8 | USA | control |
| ERR1293890 | PRJEB12449 | F | 70 | 28.6 | USA | control |
| ERR1293662 | PRJEB12449 | M | 39 | 23.5 | USA | control |
| ERR1293870 | PRJEB12449 | F | 76 | 26.9 | USA | control |
| ERR1293798 | PRJEB12449 | F | 54 | 24 | USA | colorectal cancer |
| ERR1293678 | PRJEB12449 | M | 58 | 21.1 | USA | colorectal cancer |
| ERR1293894 | PRJEB12449 | M | 65 | 31.4 | USA | colorectal cancer |
| ERR1293906 | PRJEB12449 | M | 71 | 22.6 | USA | colorectal cancer |
| ERR1293726 | PRJEB12449 | F | 63 | 25.7 | USA | colorectal cancer |
| ERR1293730 | PRJEB12449 | M | 40 | 17.4 | USA | colorectal cancer |
| ERR1293622 | PRJEB12449 | F | 59 | 20.4 | USA | colorectal cancer |
| ERR1293826 | PRJEB12449 | F | 66 | 31.5 | USA | colorectal cancer |
| ERR1293577 | PRJEB12449 | M | 74 | 23.6 | USA | colorectal cancer |
| ERR1293746 | PRJEB12449 | M | 67 | 33.2 | USA | colorectal cancer |
| ERR1293926 | PRJEB12449 | F | 45 | 22.6 | USA | control |
| ERR1293614 | PRJEB12449 | M | 65 | 21.1 | USA | colorectal cancer |
| ERR1293557 | PRJEB12449 | M | 61 | 24.4 | USA | colorectal cancer |
| ERR1293609 | PRJEB12449 | M | 63 | 25.4 | USA | control |
| ERR1293694 | PRJEB12449 | M | 63 | 24 | USA | control |
| ERR1293634 | PRJEB12449 | M | 55 | 29.6 | USA | colorectal cancer |
| ERR1293722 | PRJEB12449 | M | 43 | 32 | USA | colorectal cancer |
| ERR1293754 | PRJEB12449 | M | 51 | 24.8 | USA | control |
| ERR1293593 | PRJEB12449 | M | 67 | 30.5 | USA | control |
| ERR1293597 | PRJEB12449 | M | 58 | 24.5 | USA | control |
| ERR1293573 | PRJEB12449 | M | 85 | 22.8 | USA | colorectal cancer |
| ERR1293930 | PRJEB12449 | F | 62 | 25.8 | USA | control |
| ERR1293854 | PRJEB12449 | F | 70 | 31.2 | USA | control |
| ERR1293537 | PRJEB12449 | F | 69 | 20.8 | USA | colorectal cancer |
| ERR1293549 | PRJEB12449 | M | 60 | 33.4 | USA | control |
| ERR1293834 | PRJEB12449 | M | 71 | 27.8 | USA | colorectal cancer |
| ERR1293525 | PRJEB12449 | F | 63 | 28.3 | USA | control |
| ERR1293529 | PRJEB12449 | F | 89 | 22.7 | USA | colorectal cancer |
| ERR1293698 | PRJEB12449 | M | 63 | 24.8 | USA | control |
| ERR1293910 | PRJEB12449 | M | 72 | 23.6 | USA | control |
| ERR1293690 | PRJEB12449 | M | 65 | 32.1 | USA | colorectal cancer |
| ERR1293505 | PRJEB12449 | M | 58 | 26.8 | USA | control |
| ERR1293553 | PRJEB12449 | M | 79 | 35.2 | USA | control |
| ERR1293654 | PRJEB12449 | M | 53 | 25.4 | USA | colorectal cancer |
| ERR1293626 | PRJEB12449 | M | 59 | 23.6 | USA | control |
| ERR1293630 | PRJEB12449 | F | 83 | 16.8 | USA | colorectal cancer |
| ERR1293605 | PRJEB12449 | M | 44 | 23.4 | USA | colorectal cancer |
| ERR1293569 | PRJEB12449 | M | 74 | NA | USA | colorectal cancer |
| ERR1293766 | PRJEB12449 | M | 63 | 21.3 | USA | colorectal cancer |
| ERR1293770 | PRJEB12449 | M | 47 | 24.4 | USA | control |
| ERR1293658 | PRJEB12449 | M | 40 | 23.7 | USA | colorectal cancer |
| ERR1293517 | PRJEB12449 | M | 31 | 19.6 | USA | colorectal cancer |
| ERR1293786 | PRJEB12449 | M | 78 | 22.6 | USA | colorectal cancer |
| ERR1293830 | PRJEB12449 | M | 65 | 24.3 | USA | colorectal cancer |
| ERR1293714 | PRJEB12449 | M | 66 | 28.2 | USA | control |
| ERR1293646 | PRJEB12449 | M | 31 | 19.6 | USA | colorectal cancer |
| ERR1293922 | PRJEB12449 | M | 64 | 32.4 | USA | control |
| ERR1293513 | PRJEB12449 | M | 57 | 25.1 | USA | control |
| ERR1293882 | PRJEB12449 | M | 54 | 18.6 | USA | colorectal cancer |
| ERR1293886 | PRJEB12449 | M | 57 | 26.4 | USA | colorectal cancer |
| ERR1293533 | PRJEB12449 | M | 68 | 22.1 | USA | control |
| ERR1293686 | PRJEB12449 | F | 67 | 18.6 | USA | control |
| ERR1293902 | PRJEB12449 | M | 56 | 25.8 | USA | colorectal cancer |
| ERR1293734 | PRJEB12449 | M | 66 | 21.6 | USA | colorectal cancer |
| ERR1293778 | PRJEB12449 | M | 68 | 23 | USA | control |
| ERR1293706 | PRJEB12449 | M | 71 | 23.2 | USA | control |
| ERR1293682 | PRJEB12449 | M | 79 | 21.3 | USA | control |
| ERR1293521 | PRJEB12449 | M | 65 | 25.1 | USA | colorectal cancer |
| ERR1293718 | PRJEB12449 | F | 55 | 31.6 | USA | colorectal cancer |
| ERR1293866 | PRJEB12449 | M | 42 | 27.6 | USA | control |
| ERR1293782 | PRJEB12449 | F | 73 | 23.9 | USA | control |
| ERR1293774 | PRJEB12449 | M | 37 | 22.5 | USA | colorectal cancer |
| ERR1293585 | PRJEB12449 | M | 36 | 21.3 | USA | control |
| ERR1293842 | PRJEB12449 | F | 72 | 27.8 | USA | colorectal cancer |
| ERR1293589 | PRJEB12449 | F | 42 | 27.2 | USA | control |
| ERR1293638 | PRJEB12449 | F | 71 | NA | USA | colorectal cancer |
| ERR1293806 | PRJEB12449 | M | 60 | 23.1 | USA | control |
| ERR1293742 | PRJEB12449 | M | 76 | 23.8 | USA | control |
| ERR1293898 | PRJEB12449 | M | 48 | 21.5 | USA | control |
| ERR1293802 | PRJEB12449 | M | 45 | 24.4 | USA | control |
| ERR1293601 | PRJEB12449 | F | 68 | 38.4 | USA | control |
| ERR1293822 | PRJEB12449 | M | 66 | 22.3 | USA | colorectal cancer |
| ERR1293561 | PRJEB12449 | F | 69 | 27.8 | USA | colorectal cancer |
| ERR1293850 | PRJEB12449 | M | 56 | 22.7 | USA | control |
| ERR1293934 | PRJEB12449 | M | 84 | 23.5 | USA | colorectal cancer |
| ERR1293501 | PRJEB12449 | F | 67 | 30 | USA | colorectal cancer |
| ERR2726404 | PRJEB27928 | M | 45 | 30.7 | GER | control |
| ERR2726405 | PRJEB27928 | F | 62 | 28.5 | GER | control |
| ERR2726406 | PRJEB27928 | M | 74 | 22.9 | GER | control |
| ERR2726407 | PRJEB27928 | F | 59 | 22.5 | GER | control |
| ERR2726408 | PRJEB27928 | F | 59 | 22.7 | GER | control |
| ERR2726409 | PRJEB27928 | F | 62 | 27 | GER | control |
| ERR2726410 | PRJEB27928 | F | 63 | 25.8 | GER | colorectal cancer |
| ERR2726414 | PRJEB27928 | F | 85 | 19.7 | GER | colorectal cancer |
| ERR2726418 | PRJEB27928 | M | 80 | 28.4 | GER | colorectal cancer |
| ERR2726419 | PRJEB27928 | F | 50 | 23.4 | GER | control |
| ERR2726420 | PRJEB27928 | M | 61 | 29.9 | GER | control |
| ERR2726421 | PRJEB27928 | F | 59 | 22.1 | GER | control |
| ERR2726422 | PRJEB27928 | M | 50 | 21.6 | GER | control |
| ERR2726426 | PRJEB27928 | M | 81 | 26.1 | GER | colorectal cancer |
| ERR2726427 | PRJEB27928 | F | 55 | 18 | GER | control |
| ERR2726428 | PRJEB27928 | M | 73 | 24.8 | GER | control |
| ERR2726429 | PRJEB27928 | M | 45 | 22.1 | GER | control |
| ERR2726430 | PRJEB27928 | M | 51 | 26.3 | GER | control |
| ERR2726431 | PRJEB27928 | F | 48 | 25.4 | GER | control |
| ERR2726432 | PRJEB27928 | M | 64 | 26.8 | GER | control |
| ERR2726433 | PRJEB27928 | M | 75 | 21.5 | GER | control |
| ERR2726434 | PRJEB27928 | F | 57 | 22.2 | GER | control |
| ERR2726435 | PRJEB27928 | M | 64 | 27.6 | GER | control |
| ERR2726436 | PRJEB27928 | F | 60 | 25.8 | GER | control |
| ERR2726440 | PRJEB27928 | M | 61 | 25.2 | GER | colorectal cancer |
| ERR2726441 | PRJEB27928 | F | 29 | 30.5 | GER | control |
| ERR2726442 | PRJEB27928 | M | 44 | 25.1 | GER | control |
| ERR2726443 | PRJEB27928 | M | 76 | 29.5 | GER | control |
| ERR2726444 | PRJEB27928 | M | 56 | 25 | GER | control |
| ERR2726445 | PRJEB27928 | M | 53 | 18.5 | GER | control |
| ERR2726446 | PRJEB27928 | F | 61 | 25.4 | GER | control |
| ERR2726447 | PRJEB27928 | M | 67 | 24.4 | GER | control |
| ERR2726448 | PRJEB27928 | F | 67 | 30.1 | GER | colorectal cancer |
| ERR2726449 | PRJEB27928 | M | 53 | 26.2 | GER | control |
| ERR2726450 | PRJEB27928 | M | 75 | 30.1 | GER | control |
| ERR2726451 | PRJEB27928 | F | 53 | 24.1 | GER | control |
| ERR2726452 | PRJEB27928 | M | 41 | 23.8 | GER | control |
| ERR2726456 | PRJEB27928 | M | 61 | 21.6 | GER | colorectal cancer |
| ERR2726457 | PRJEB27928 | M | 46 | 26.3 | GER | control |
| ERR2726458 | PRJEB27928 | M | 64 | 24.6 | GER | control |
| ERR2726459 | PRJEB27928 | F | 63 | 20.2 | GER | control |
| ERR2726460 | PRJEB27928 | M | 75 | 22.9 | GER | control |
| ERR2726461 | PRJEB27928 | M | 50 | 22.6 | GER | control |
| ERR2726462 | PRJEB27928 | M | 65 | 32.7 | GER | control |
| ERR2726463 | PRJEB27928 | M | 79 | 22.4 | GER | colorectal cancer |
| ERR2726470 | PRJEB27928 | M | 81 | 24 | GER | colorectal cancer |
| ERR2726471 | PRJEB27928 | M | 61 | 27 | GER | control |
| ERR2726472 | PRJEB27928 | F | 75 | 19.3 | GER | control |
| ERR2726473 | PRJEB27928 | F | 57 | 21.2 | GER | control |
| ERR2726474 | PRJEB27928 | M | 66 | 30.3 | GER | control |
| ERR2726475 | PRJEB27928 | M | 64 | 25.4 | GER | control |
| ERR2726479 | PRJEB27928 | F | 61 | 24.1 | GER | colorectal cancer |
| ERR2726483 | PRJEB27928 | F | 63 | 28.8 | GER | colorectal cancer |
| ERR2726484 | PRJEB27928 | F | 50 | 32.1 | GER | colorectal cancer |
| ERR2726485 | PRJEB27928 | F | 64 | 21.1 | GER | control |
| ERR2726486 | PRJEB27928 | M | 73 | 24.5 | GER | control |
| ERR2726487 | PRJEB27928 | F | 64 | 22.8 | GER | control |
| ERR2726488 | PRJEB27928 | F | 56 | 30 | GER | colorectal cancer |
| ERR2726492 | PRJEB27928 | F | 60 | 35.8 | GER | colorectal cancer |
| ERR2726493 | PRJEB27928 | F | 58 | 23.8 | GER | control |
| ERR2726497 | PRJEB27928 | M | 60 | 19.7 | GER | colorectal cancer |
| ERR2726498 | PRJEB27928 | F | 57 | 22.2 | GER | control |
| ERR2726499 | PRJEB27928 | M | 48 | 23 | GER | control |
| ERR2726500 | PRJEB27928 | F | 53 | 23 | GER | control |
| ERR2726501 | PRJEB27928 | F | 62 | 21.3 | GER | colorectal cancer |
| ERR2726502 | PRJEB27928 | F | 60 | 23 | GER | control |
| ERR2726503 | PRJEB27928 | F | 60 | 21.9 | GER | colorectal cancer |
| ERR2726504 | PRJEB27928 | F | 69 | 25.3 | GER | control |
| ERR2726505 | PRJEB27928 | M | 28 | 24.6 | GER | control |
| ERR2726506 | PRJEB27928 | F | 54 | 24.6 | GER | control |
| ERR2726510 | PRJEB27928 | M | 52 | 29.9 | GER | colorectal cancer |
| ERR2726511 | PRJEB27928 | F | 51 | 25.6 | GER | control |
| ERR2726512 | PRJEB27928 | F | 58 | 27 | GER | control |
| ERR2726516 | PRJEB27928 | F | 66 | 13.3 | GER | colorectal cancer |
| ERR2726517 | PRJEB27928 | F | 45 | 27.2 | GER | control |
| ERR2726521 | PRJEB27928 | M | 84 | 24 | GER | colorectal cancer |
| ERR2726525 | PRJEB27928 | M | 73 | 26.6 | GER | colorectal cancer |
| ERR2726526 | PRJEB27928 | F | 59 | 32.3 | GER | control |
| ERR2726530 | PRJEB27928 | M | 59 | 26.5 | GER | colorectal cancer |
| ERR2726531 | PRJEB27928 | M | 37 | 24.5 | GER | control |
| ERR2726532 | PRJEB27928 | M | 39 | 24.9 | GER | control |
| ERR2726533 | PRJEB27928 | M | 70 | 26.7 | GER | control |
| ERR478962 | ERP005534 | M | 53 | 32 | FRA | control |
| ERR478965 | ERP005534 | M | 35 | 23 | FRA | control |
| ERR478969 | ERP005534 | M | 67 | 28 | FRA | colorectal cancer |
| ERR478971 | ERP005534 | M | 74 | 27 | FRA | control |
| ERR478975 | ERP005534 | M | 29 | 24.49 | FRA | control |
| ERR478978 | ERP005534 | M | 61 | 23 | FRA | control |
| ERR478982 | ERP005534 | F | 65 | 24 | FRA | control |
| ERR478984 | ERP005534 | M | 58 | 24 | FRA | colorectal cancer |
| ERR478987 | ERP005534 | M | 63 | NA | FRA | control |
| ERR478991 | ERP005534 | F | 77 | 22 | FRA | control |
| ERR478994 | ERP005534 | F | 62 | 21 | FRA | control |
| ERR478999 | ERP005534 | M | 63 | 26 | FRA | adenoma |
| ERR479003 | ERP005534 | M | 67 | 21 | FRA | control |
| ERR479004 | ERP005534 | M | 25 | 24.15 | FRA | control |
| ERR479006 | ERP005534 | M | 64 | 26 | FRA | adenoma |
| ERR479011 | ERP005534 | M | 62 | 26 | FRA | colorectal cancer |
| ERR479013 | ERP005534 | M | 58 | 22 | FRA | adenoma |
| ERR479015 | ERP005534 | M | 67 | 27 | FRA | control |
| ERR479020 | ERP005534 | F | 44 | 26 | FRA | control |
| ERR479023 | ERP005534 | F | 56 | NA | FRA | control |
| ERR479024 | ERP005534 | F | 81 | 38.71 | FRA | colorectal cancer |
| ERR479029 | ERP005534 | M | 51 | 30 | FRA | colorectal cancer |
| ERR479035 | ERP005534 | M | 70 | 22 | FRA | control |
| ERR479038 | ERP005534 | F | 59 | 20 | FRA | colorectal cancer |
| ERR479040 | ERP005534 | F | 56 | NA | FRA | colorectal cancer |
| ERR479042 | ERP005534 | F | 37 | 18 | FRA | control |
| ERR479043 | ERP005534 | F | 46 | 29 | FRA | control |
| ERR479047 | ERP005534 | F | 61 | 24 | FRA | control |
| ERR479050 | ERP005534 | M | 54 | 26 | FRA | control |
| ERR479053 | ERP005534 | F | 72 | 37 | FRA | colorectal cancer |
| ERR479057 | ERP005534 | M | 68 | 23 | FRA | adenoma |
| ERR479060 | ERP005534 | F | 71 | 23 | FRA | control |
| ERR479064 | ERP005534 | M | 59 | 25 | FRA | control |
| ERR479066 | ERP005534 | M | 63 | 25 | FRA | colorectal cancer |
| ERR479069 | ERP005534 | M | 62 | 29 | FRA | adenoma |
| ERR479072 | ERP005534 | M | 82 | 24.49 | FRA | colorectal cancer |
| ERR479075 | ERP005534 | M | 77 | 23 | FRA | adenoma |
| ERR479078 | ERP005534 | M | 60 | 26 | FRA | colorectal cancer |
| ERR479082 | ERP005534 | M | 62 | 23 | FRA | control |
| ERR479084 | ERP005534 | F | 69 | 24 | FRA | colorectal cancer |
| ERR479086 | ERP005534 | M | 59 | 31 | FRA | adenoma |
| ERR479088 | ERP005534 | M | 60 | 27 | FRA | adenoma |
| ERR479092 | ERP005534 | F | 52 | 20 | FRA | control |
| ERR479094 | ERP005534 | M | 72 | 24 | FRA | control |
| ERR479098 | ERP005534 | M | 89 | 24 | FRA | adenoma |
| ERR479100 | ERP005534 | M | 64 | 27 | FRA | control |
| ERR479101 | ERP005534 | M | 50 | 25 | FRA | control |
| ERR479104 | ERP005534 | M | 66 | 24 | FRA | adenoma |
| ERR479106 | ERP005534 | M | 53 | 30 | FRA | adenoma |
| ERR479109 | ERP005534 | M | 68 | 26 | FRA | control |
| ERR479112 | ERP005534 | M | 64 | 21 | FRA | colorectal cancer |
| ERR479117 | ERP005534 | M | 80 | 29.4 | FRA | colorectal cancer |
| ERR479119 | ERP005534 | F | 63 | 27 | FRA | control |
| ERR479123 | ERP005534 | F | 62 | 21 | FRA | adenoma |
| ERR479127 | ERP005534 | M | 73 | 40 | FRA | colorectal cancer |
| ERR479129 | ERP005534 | F | 68 | 25 | FRA | control |
| ERR479134 | ERP005534 | M | 63 | 25 | FRA | control |
| ERR479137 | ERP005534 | F | 59 | 21 | FRA | adenoma |
| ERR479139 | ERP005534 | F | 79 | 22 | FRA | colorectal cancer |
| ERR479140 | ERP005534 | F | 87 | 32.42 | FRA | colorectal cancer |
| ERR479144 | ERP005534 | F | 73 | 24 | FRA | colorectal cancer |
| ERR479147 | ERP005534 | F | 74 | 19 | FRA | colorectal cancer |
| ERR479148 | ERP005534 | M | 78 | 19 | FRA | colorectal cancer |
| ERR479153 | ERP005534 | F | 67 | 28 | FRA | control |
| ERR479155 | ERP005534 | F | 59 | NA | FRA | adenoma |
| ERR479158 | ERP005534 | F | 62 | 20 | FRA | control |
| ERR479163 | ERP005534 | M | 66 | 22 | FRA | adenoma |
| ERR479166 | ERP005534 | M | 75 | 37 | FRA | colorectal cancer |
| ERR479167 | ERP005534 | F | 55 | 20 | FRA | control |
| ERR479170 | ERP005534 | M | 67 | 25 | FRA | control |
| ERR479174 | ERP005534 | M | 65 | 24 | FRA | colorectal cancer |
| ERR479176 | ERP005534 | F | 54 | 22 | FRA | colorectal cancer |
| ERR479178 | ERP005534 | M | 67 | 25 | FRA | colorectal cancer |
| ERR479182 | ERP005534 | M | 63 | 27 | FRA | adenoma |
| ERR479185 | ERP005534 | M | 66 | 25 | FRA | control |
| ERR479189 | ERP005534 | F | 72 | 32 | FRA | adenoma |
| ERR479191 | ERP005534 | F | 52 | 23 | FRA | adenoma |
| ERR479194 | ERP005534 | F | 62 | 21 | FRA | colorectal cancer |
| ERR479195 | ERP005534 | F | 66 | 28 | FRA | control |
| ERR479199 | ERP005534 | F | 87 | 15.15 | FRA | colorectal cancer |
| ERR479202 | ERP005534 | F | 53 | 33 | FRA | adenoma |
| ERR479204 | ERP005534 | F | 61 | 23 | FRA | control |
| ERR479208 | ERP005534 | F | 60 | 26 | FRA | adenoma |
| ERR479210 | ERP005534 | F | 63 | 22 | FRA | control |
| ERR479213 | ERP005534 | M | 63 | 26 | FRA | adenoma |
| ERR479218 | ERP005534 | M | 64 | 28 | FRA | adenoma |
| ERR479220 | ERP005534 | M | 53 | 25 | FRA | colorectal cancer |
| ERR479224 | ERP005534 | M | 72 | 22 | FRA | colorectal cancer |
| ERR479226 | ERP005534 | M | 76 | NA | FRA | colorectal cancer |
| ERR479227 | ERP005534 | M | 45 | 24 | FRA | adenoma |
| ERR479231 | ERP005534 | M | 52 | 33 | FRA | adenoma |
| ERR479236 | ERP005534 | M | 64 | 30 | FRA | colorectal cancer |
| ERR479240 | ERP005534 | F | 45 | 24 | FRA | adenoma |
| ERR479241 | ERP005534 | M | 68 | 21 | FRA | adenoma |
| ERR479244 | ERP005534 | F | 70 | 26 | FRA | colorectal cancer |
| ERR479245 | ERP005534 | F | 80 | 23.19 | FRA | colorectal cancer |
| ERR479249 | ERP005534 | F | 76 | 34 | FRA | adenoma |
| ERR479253 | ERP005534 | M | 76 | 25 | FRA | colorectal cancer |
| ERR479255 | ERP005534 | M | 68 | 23 | FRA | colorectal cancer |
| ERR479262 | ERP005534 | F | 61 | 34 | FRA | control |
| ERR479264 | ERP005534 | F | 69 | 25 | FRA | control |
| ERR479268 | ERP005534 | F | 49 | 23 | FRA | control |
| ERR479271 | ERP005534 | F | 63 | 23 | FRA | control |
| ERR479275 | ERP005534 | F | 59 | 25 | FRA | control |
| ERR479279 | ERP005534 | F | 69 | 25 | FRA | colorectal cancer |
| ERR479280 | ERP005534 | M | 55 | 24 | FRA | adenoma |
| ERR479285 | ERP005534 | F | 50 | 24 | FRA | colorectal cancer |
| ERR479287 | ERP005534 | M | 70 | 25 | FRA | control |
| ERR479292 | ERP005534 | F | 74 | 26 | FRA | colorectal cancer |
| ERR479295 | ERP005534 | M | 62 | 24 | FRA | control |
| ERR479300 | ERP005534 | M | 68 | 36 | FRA | adenoma |
| ERR479303 | ERP005534 | M | 44 | 20 | FRA | colorectal cancer |
| ERR479306 | ERP005534 | M | 73 | 26 | FRA | adenoma |
| ERR479309 | ERP005534 | M | 65 | 26 | FRA | colorectal cancer |
| ERR479314 | ERP005534 | F | 64 | 30 | FRA | control |
| ERR479317 | ERP005534 | F | 69 | 30 | FRA | colorectal cancer |
| ERR479319 | ERP005534 | M | 65 | 30 | FRA | control |
| ERR479321 | ERP005534 | F | 60 | 28 | FRA | control |
| ERR479326 | ERP005534 | F | 64 | 20 | FRA | control |
| ERR479328 | ERP005534 | M | 49 | 20 | FRA | adenoma |
| ERR479330 | ERP005534 | M | 68 | 26 | FRA | adenoma |
| ERR479332 | ERP005534 | M | 59 | 27 | FRA | colorectal cancer |
| ERR479336 | ERP005534 | M | 51 | 24 | FRA | colorectal cancer |
| ERR479339 | ERP005534 | M | 57 | 24 | FRA | control |
| ERR479342 | ERP005534 | F | 38 | 22 | FRA | control |
| ERR479344 | ERP005534 | M | 66 | 29 | FRA | adenoma |
| ERR479346 | ERP005534 | M | 60 | 24 | FRA | adenoma |
| ERR479348 | ERP005534 | F | 48 | 24 | FRA | colorectal cancer |
| ERR479351 | ERP005534 | F | 45 | 28 | FRA | colorectal cancer |
| ERR479354 | ERP005534 | M | 63 | 22 | FRA | colorectal cancer |
| ERR479356 | ERP005534 | F | 85 | 20.31 | FRA | colorectal cancer |
| ERR479358 | ERP005534 | M | 79 | 30 | FRA | colorectal cancer |
| ERR479359 | ERP005534 | M | 73 | 17 | FRA | colorectal cancer |
| ERR479364 | ERP005534 | M | 71 | 29 | FRA | adenoma |
| ERR479367 | ERP005534 | F | 73 | 26 | FRA | colorectal cancer |
| ERR479371 | ERP005534 | F | 67 | 23 | FRA | control |
| ERR479374 | ERP005534 | M | 74 | 27 | FRA | control |
| ERR479379 | ERP005534 | M | 84 | 23 | FRA | control |
| ERR479383 | ERP005534 | F | 78 | 23 | FRA | adenoma |
| ERR479385 | ERP005534 | M | 63 | 30 | FRA | adenoma |
| ERR479388 | ERP005534 | F | 71 | 22 | FRA | adenoma |
| ERR479390 | ERP005534 | M | 63 | 31 | FRA | colorectal cancer |
| ERR479392 | ERP005534 | M | 65 | 30 | FRA | control |
| ERR479394 | ERP005534 | F | 59 | 25 | FRA | control |
| ERR479395 | ERP005534 | M | 64 | 28 | FRA | adenoma |
| ERR479398 | ERP005534 | F | 80 | 21 | FRA | control |
| ERR479403 | ERP005534 | M | 52 | 24 | FRA | control |
| ERR479407 | ERP005534 | F | 63 | 21 | FRA | control |
| ERR479409 | ERP005534 | M | 66 | 20 | FRA | adenoma |
| ERR479414 | ERP005534 | F | 53 | 30 | FRA | control |
| ERR479416 | ERP005534 | M | 63 | 29 | FRA | colorectal cancer |
| ERR479418 | ERP005534 | F | 55 | 25 | FRA | colorectal cancer |
| DRR127478 | DRA006684 | F | 66 | 22.600263 | JPN | colorectal cancer |
| DRR127481 | DRA006684 | M | 61 | 28.2933454 | JPN | colorectal cancer |
| DRR127485 | DRA006684 | F | 70 | 21.9075825 | JPN | colorectal cancer |
| DRR127488 | DRA006684 | M | 72 | 22.1367397 | JPN | control |
| DRR127491 | DRA006684 | F | 62 | 21.0828133 | JPN | colorectal cancer |
| DRR127507 | DRA006684 | M | 65 | 21.3039486 | JPN | colorectal cancer |
| DRR127509 | DRA006684 | M | 59 | 25.4580659 | JPN | colorectal cancer |
| DRR127514 | DRA006684 | M | 78 | 22.6799524 | JPN | colorectal cancer |
| DRR127515 | DRA006684 | M | 30 | 25.6895619 | JPN | colorectal cancer |
| DRR127517 | DRA006684 | F | 69 | 23.5555556 | JPN | colorectal cancer |
| DRR127519 | DRA006684 | F | 57 | 18.3690034 | JPN | colorectal cancer |
| DRR127521 | DRA006684 | M | 38 | 24.3910588 | JPN | colorectal cancer |
| DRR127524 | DRA006684 | M | 73 | 25.8166302 | JPN | control |
| DRR127532 | DRA006684 | F | 59 | 20.0288415 | JPN | control |
| DRR127535 | DRA006684 | M | 64 | 20.5191341 | JPN | control |
| DRR127536 | DRA006684 | F | 44 | 26.6727633 | JPN | colorectal cancer |
| DRR127537 | DRA006684 | F | 50 | 20.3427978 | JPN | control |
| DRR127546 | DRA006684 | F | 67 | 26.0416667 | JPN | control |
| DRR127552 | DRA006684 | M | 45 | 22.2222222 | JPN | control |
| DRR127557 | DRA006684 | M | 62 | 24.6181038 | JPN | colorectal cancer |
| DRR127559 | DRA006684 | M | 46 | 19.1511918 | JPN | colorectal cancer |
| DRR127567 | DRA006684 | F | 70 | 17.3484295 | JPN | colorectal cancer |
| DRR127579 | DRA006684 | M | 70 | 20.5761317 | JPN | colorectal cancer |
| DRR127580 | DRA006684 | F | 63 | 17.4817898 | JPN | colorectal cancer |
| DRR127583 | DRA006684 | F | 65 | 19.8189271 | JPN | control |
| DRR127588 | DRA006684 | M | 76 | 22.1002896 | JPN | control |
| DRR127594 | DRA006684 | M | 69 | 21.5138585 | JPN | colorectal cancer |
| DRR127596 | DRA006684 | F | 76 | 23.4220907 | JPN | control |
| DRR127597 | DRA006684 | F | 53 | 18.6620147 | JPN | control |
| DRR127603 | DRA006684 | F | 45 | 23.4223684 | JPN | colorectal cancer |
| DRR127613 | DRA006684 | F | 75 | 22.6666667 | JPN | control |
| DRR127616 | DRA006684 | M | 78 | 22.8623685 | JPN | control |
| DRR127617 | DRA006684 | F | 59 | 22.65625 | JPN | colorectal cancer |
| DRR127619 | DRA006684 | M | 45 | 23.9512266 | JPN | control |
| DRR127625 | DRA006684 | M | 64 | 23.1084346 | JPN | colorectal cancer |
| DRR127628 | DRA006684 | M | 76 | 21.799308 | JPN | control |
| DRR127634 | DRA006684 | F | 42 | 25.5937371 | JPN | control |
| DRR127638 | DRA006684 | F | 32 | 22.3477821 | JPN | colorectal cancer |
| DRR127646 | DRA006684 | F | 49 | 19.3821447 | JPN | colorectal cancer |
| DRR127647 | DRA006684 | F | 67 | 20.1745537 | JPN | colorectal cancer |
| DRR127649 | DRA006684 | F | 49 | 22.3081499 | JPN | control |
| DRR127666 | DRA006684 | M | 78 | 25.909457 | JPN | colorectal cancer |
| DRR127669 | DRA006684 | F | 54 | 20.5688066 | JPN | colorectal cancer |
| DRR127672 | DRA006684 | M | 42 | 26.1738326 | JPN | control |
| DRR127674 | DRA006684 | F | 59 | 20.9566075 | JPN | colorectal cancer |
| DRR127683 | DRA006684 | M | 40 | 22.9819018 | JPN | control |
| DRR127685 | DRA006684 | M | 70 | 24.2424242 | JPN | colorectal cancer |
| DRR127692 | DRA006684 | M | 76 | 25.9695291 | JPN | control |
| DRR127700 | DRA006684 | F | 46 | 17.4449268 | JPN | colorectal cancer |
| DRR127704 | DRA006684 | F | 49 | 21.230572 | JPN | control |
| DRR127707 | DRA006684 | M | 63 | 22.0580512 | JPN | control |
| DRR127708 | DRA006684 | M | 62 | 25.5937371 | JPN | colorectal cancer |
| DRR127713 | DRA006684 | M | 49 | 27.3981757 | JPN | control |
| DRR127718 | DRA006684 | F | 75 | 22.2222222 | JPN | colorectal cancer |
| DRR127720 | DRA006684 | F | 73 | 20.5456936 | JPN | colorectal cancer |
| DRR127721 | DRA006684 | M | 51 | 28.3848022 | JPN | control |
| DRR127724 | DRA006684 | F | 66 | 25.4108997 | JPN | control |
| DRR127728 | DRA006684 | F | 71 | 18.0172064 | JPN | control |
| DRR127731 | DRA006684 | M | 66 | 23.6652444 | JPN | control |
| DRR127736 | DRA006684 | F | 64 | 23.1910946 | JPN | control |
| DRR127748 | DRA006684 | M | 44 | 22.0521902 | JPN | control |
| DRR127751 | DRA006684 | M | 74 | 20.6851502 | JPN | control |
| DRR127752 | DRA006684 | M | 70 | 23.6614386 | JPN | control |
| DRR127754 | DRA006684 | M | 55 | 24.5778672 | JPN | colorectal cancer |
| DRR127755 | DRA006684 | M | 70 | 25.7116621 | JPN | control |
| DRR127756 | DRA006684 | M | 75 | 22.3081499 | JPN | control |
| DRR127762 | DRA006684 | M | 76 | 23.7953599 | JPN | control |
| DRR127763 | DRA006684 | M | 76 | 25.8805706 | JPN | control |
| DRR127767 | DRA006684 | M | 28 | 26.2975779 | JPN | colorectal cancer |
| DRR127774 | DRA006684 | F | 69 | 25.6369172 | JPN | colorectal cancer |
| DRR127776 | DRA006684 | F | 73 | 20.703125 | JPN | control |
| DRR127777 | DRA006684 | F | 77 | 23.634033 | JPN | control |
| DRR127789 | DRA006684 | M | 51 | 20.7612457 | JPN | colorectal cancer |
| DRR162775 | DRA006684 | M | 65 | 21.5645449 | JPN | control |
| DRR162776 | DRA006684 | F | 66 | 22.3477821 | JPN | control |
| DRR162777 | DRA006684 | M | 48 | 23.0300454 | JPN | colorectal cancer |
| DRR162778 | DRA006684 | M | 65 | 21.383942 | JPN | colorectal cancer |
| DRR162779 | DRA006684 | M | 70 | 18.9619274 | JPN | colorectal cancer |
| DRR171459 | DRA008156 | F | 69 | 22.50692521 | JPN | colorectal cancer |
| DRR171460 | DRA008156 | M | 58 | 24.02380867 | JPN | colorectal cancer |
| DRR171464 | DRA008156 | F | 64 | 18.02595738 | JPN | colorectal cancer |
| DRR171468 | DRA008156 | M | 76 | 22.86253448 | JPN | control |
| DRR171473 | DRA008156 | F | 68 | 24.32322868 | JPN | control |
| DRR171476 | DRA008156 | F | 67 | 21.90758246 | JPN | control |
| DRR171478 | DRA008156 | F | 78 | 20.69049454 | JPN | control |
| DRR171482 | DRA008156 | M | 61 | 22.39541342 | JPN | colorectal cancer |
| DRR171483 | DRA008156 | M | 58 | 22.53061224 | JPN | colorectal cancer |
| DRR171484 | DRA008156 | M | 69 | 24.6097337 | JPN | colorectal cancer |
| DRR171486 | DRA008156 | M | 67 | 25.0995016 | JPN | control |
| DRR171487 | DRA008156 | F | 62 | 25.15315023 | JPN | control |
| DRR171488 | DRA008156 | M | 66 | 20.54569362 | JPN | colorectal cancer |
| DRR171489 | DRA008156 | F | 74 | 19.43634597 | JPN | colorectal cancer |
| DRR171491 | DRA008156 | F | 48 | 22.89281998 | JPN | colorectal cancer |
| DRR171496 | DRA008156 | F | 70 | 25.33333333 | JPN | control |
| DRR171497 | DRA008156 | M | 77 | 20.43816558 | JPN | control |
| DRR171500 | DRA008156 | M | 60 | 19.48696145 | JPN | control |
| DRR171503 | DRA008156 | M | 55 | 24.09297052 | JPN | control |
| DRR171504 | DRA008156 | M | 59 | 24.50894577 | JPN | colorectal cancer |
| DRR171505 | DRA008156 | M | 48 | 28.30599506 | JPN | colorectal cancer |
| DRR171506 | DRA008156 | M | 69 | 22.40817899 | JPN | control |
| DRR171507 | DRA008156 | M | 67 | 30.47721924 | JPN | colorectal cancer |
| DRR171508 | DRA008156 | M | 46 | 22.85714286 | JPN | colorectal cancer |
| DRR171509 | DRA008156 | M | 73 | 23.72528616 | JPN | colorectal cancer |
| DRR171510 | DRA008156 | F | 54 | 20.77562327 | JPN | control |
| DRR171511 | DRA008156 | F | 68 | 18.66201469 | JPN | control |
| DRR171512 | DRA008156 | M | 37 | 25.01352082 | JPN | control |
| DRR171513 | DRA008156 | M | 61 | 27.47562949 | JPN | colorectal cancer |
| DRR171514 | DRA008156 | M | 46 | 22.47120876 | JPN | control |
| DRR171515 | DRA008156 | F | 46 | 23.046875 | JPN | control |
| DRR171516 | DRA008156 | M | 67 | 24.67550027 | JPN | colorectal cancer |
| DRR171517 | DRA008156 | M | 66 | 22.13673973 | JPN | colorectal cancer |
| DRR171518 | DRA008156 | M | 74 | 22.54657648 | JPN | colorectal cancer |
| DRR171519 | DRA008156 | M | 55 | 25.35154137 | JPN | colorectal cancer |
| DRR171520 | DRA008156 | M | 58 | 20.30369756 | JPN | control |
| DRR171521 | DRA008156 | M | 66 | 22.14532872 | JPN | colorectal cancer |
| DRR171522 | DRA008156 | F | 43 | 16.796875 | JPN | control |
| DRR171523 | DRA008156 | F | 60 | 20.02884153 | JPN | colorectal cancer |
| DRR171524 | DRA008156 | M | 52 | 25.01352082 | JPN | control |
| DRR171525 | DRA008156 | F | 65 | 18.26150475 | JPN | colorectal cancer |
| DRR171526 | DRA008156 | M | 65 | 20.01549587 | JPN | colorectal cancer |
| DRR171527 | DRA008156 | F | 58 | 18.5528757 | JPN | control |
| DRR171528 | DRA008156 | M | 58 | 23.1206236 | JPN | colorectal cancer |
| DRR171529 | DRA008156 | M | 75 | 19.70553242 | JPN | colorectal cancer |
| DRR171530 | DRA008156 | F | 61 | 22.47658689 | JPN | colorectal cancer |
| DRR171531 | DRA008156 | M | 75 | 22.86253448 | JPN | colorectal cancer |
| DRR171532 | DRA008156 | M | 72 | 23.66524436 | JPN | colorectal cancer |
| DRR171533 | DRA008156 | M | 66 | 23.87511478 | JPN | colorectal cancer |
| DRR171534 | DRA008156 | F | 58 | 17.1875 | JPN | colorectal cancer |
| DRR171535 | DRA008156 | M | 66 | 24.8015873 | JPN | control |
| DRR171536 | DRA008156 | F | 73 | 21.60410478 | JPN | control |
| DRR171537 | DRA008156 | M | 48 | 25.64891762 | JPN | colorectal cancer |
| DRR171538 | DRA008156 | M | 73 | 23.87511478 | JPN | colorectal cancer |
| DRR171539 | DRA008156 | F | 65 | 20.3125 | JPN | colorectal cancer |
| DRR171540 | DRA008156 | F | 61 | 21.07719523 | JPN | control |
| DRR171541 | DRA008156 | M | 75 | 20.9566075 | JPN | colorectal cancer |
| DRR171542 | DRA008156 | F | 71 | 17.34842951 | JPN | control |
| DRR171543 | DRA008156 | M | 48 | 21.67211013 | JPN | control |
| DRR171545 | DRA008156 | M | 72 | 19.94321255 | JPN | colorectal cancer |
| DRR171546 | DRA008156 | M | 63 | 23.50780533 | JPN | colorectal cancer |
| DRR171548 | DRA008156 | M | 44 | 28.99789384 | JPN | colorectal cancer |
| DRR171549 | DRA008156 | M | 44 | 23.83673469 | JPN | control |
| DRR171550 | DRA008156 | M | 61 | 22.58955144 | JPN | colorectal cancer |
| DRR171551 | DRA008156 | M | 46 | 25.40281608 | JPN | colorectal cancer |
| DRR171552 | DRA008156 | M | 77 | 20.57142857 | JPN | control |
| DRR171553 | DRA008156 | M | 67 | 21.33333333 | JPN | colorectal cancer |
| DRR171554 | DRA008156 | M | 62 | 19.83471074 | JPN | colorectal cancer |
| DRR171555 | DRA008156 | F | 66 | 21.23057202 | JPN | colorectal cancer |
| DRR171556 | DRA008156 | M | 76 | 19.00390835 | JPN | colorectal cancer |
| DRR171557 | DRA008156 | F | 63 | 27.54820937 | JPN | control |
| DRR171558 | DRA008156 | M | 62 | 21.09375 | JPN | colorectal cancer |
| DRR171559 | DRA008156 | M | 55 | 23.38868656 | JPN | control |
| DRR171560 | DRA008156 | M | 32 | 18.73278237 | JPN | control |
| DRR171561 | DRA008156 | M | 77 | 20.43816558 | JPN | control |
| DRR171562 | DRA008156 | M | 34 | 21.85727788 | JPN | control |
| DRR171563 | DRA008156 | M | 37 | 25.34435262 | JPN | control |
| DRR171564 | DRA008156 | F | 68 | 19.22337562 | JPN | control |
| DRR171565 | DRA008156 | M | 51 | 19.15708812 | JPN | control |
| DRR171566 | DRA008156 | M | 63 | 21.10726644 | JPN | control |
| DRR171567 | DRA008156 | F | 78 | 21.2191358 | JPN | colorectal cancer |
| DRR171568 | DRA008156 | F | 51 | 19.43301326 | JPN | control |
| DRR171569 | DRA008156 | M | 72 | 23.05456246 | JPN | control |
| DRR171570 | DRA008156 | F | 66 | 22.0603538 | JPN | colorectal cancer |
| DRR171571 | DRA008156 | M | 73 | 21.20310523 | JPN | colorectal cancer |
| DRR171572 | DRA008156 | M | 54 | 25.46938776 | JPN | colorectal cancer |
| DRR171573 | DRA008156 | M | 40 | 27.37966468 | JPN | control |
| DRR171575 | DRA008156 | M | 47 | 26.17519452 | JPN | control |
| DRR171576 | DRA008156 | M | 64 | 22.40587695 | JPN | colorectal cancer |
| DRR171577 | DRA008156 | M | 57 | 26.25958475 | JPN | control |
| DRR171578 | DRA008156 | M | 54 | 23.52941176 | JPN | control |
| DRR171579 | DRA008156 | M | 63 | 24.44180209 | JPN | control |
| DRR171582 | DRA008156 | F | 44 | 21.63114885 | JPN | control |
| DRR171583 | DRA008156 | M | 55 | 24.50894577 | JPN | control |
| DRR171585 | DRA008156 | F | 79 | 24.609375 | JPN | control |
| DRR171586 | DRA008156 | M | 38 | 22.4996371 | JPN | control |
| DRR171588 | DRA008156 | F | 54 | 20.23950076 | JPN | control |
| DRR171589 | DRA008156 | M | 61 | 26.42356982 | JPN | control |
| DRR171590 | DRA008156 | M | 50 | 23.66143862 | JPN | colorectal cancer |
| DRR171591 | DRA008156 | M | 55 | 22.91303307 | JPN | control |
| DRR171592 | DRA008156 | M | 66 | 28.9340689 | JPN | colorectal cancer |
| DRR171594 | DRA008156 | M | 51 | 26.64359862 | JPN | colorectal cancer |
| DRR171595 | DRA008156 | F | 58 | 17.90885536 | JPN | control |
| DRR171597 | DRA008156 | F | 59 | 19.14672216 | JPN | colorectal cancer |
| DRR171598 | DRA008156 | F | 64 | 16.42365006 | JPN | control |
| DRR171599 | DRA008156 | F | 61 | 17.84651993 | JPN | colorectal cancer |
| DRR171600 | DRA008156 | M | 68 | 22.95908766 | JPN | colorectal cancer |
| DRR171601 | DRA008156 | F | 40 | 17.63085399 | JPN | control |
| DRR171602 | DRA008156 | F | 46 | 20.3125 | JPN | control |
| DRR171603 | DRA008156 | M | 72 | 22.94811574 | JPN | control |
| DRR171604 | DRA008156 | F | 62 | 21.92612582 | JPN | control |
| DRR171605 | DRA008156 | M | 47 | 21.4532872 | JPN | control |
| DRR171606 | DRA008156 | F | 71 | 18.49112426 | JPN | colorectal cancer |
| DRR171607 | DRA008156 | F | 47 | 18.44254582 | JPN | control |
| DRR171610 | DRA008156 | F | 67 | 23.93606231 | JPN | control |
| DRR171613 | DRA008156 | M | 56 | 23.83673469 | JPN | control |
| DRR171614 | DRA008156 | M | 72 | 21.71925011 | JPN | control |
| DRR171615 | DRA008156 | F | 73 | 21.05170826 | JPN | colorectal cancer |
| DRR171616 | DRA008156 | M | 54 | 27.33564014 | JPN | control |
| DRR171617 | DRA008156 | F | 63 | 19.14672216 | JPN | control |
| DRR171618 | DRA008156 | M | 75 | 20.13477975 | JPN | control |
| DRR171619 | DRA008156 | F | 68 | 25.68370987 | JPN | colorectal cancer |
| DRR171620 | DRA008156 | F | 66 | 23.03004535 | JPN | control |
| DRR171621 | DRA008156 | F | 62 | 20.23950076 | JPN | control |
| DRR171622 | DRA008156 | F | 66 | 23.72528616 | JPN | control |
| DRR171623 | DRA008156 | M | 55 | 23.56663223 | JPN | control |
| DRR171624 | DRA008156 | M | 64 | 24.48979592 | JPN | colorectal cancer |
| DRR171625 | DRA008156 | F | 69 | 23.53036634 | JPN | control |
| DRR171626 | DRA008156 | F | 71 | 26.63495838 | JPN | control |
| DRR171627 | DRA008156 | M | 58 | 20.51508648 | JPN | colorectal cancer |
| DRR171628 | DRA008156 | F | 68 | 21.19274242 | JPN | control |
| DRR171631 | DRA008156 | M | 64 | 22.58955144 | JPN | colorectal cancer |
| DRR171633 | DRA008156 | F | 69 | 18.17867036 | JPN | colorectal cancer |
| DRR171634 | DRA008156 | M | 37 | 19.26717144 | JPN | control |
| DRR171636 | DRA008156 | F | 49 | 19.72386588 | JPN | control |
| DRR171637 | DRA008156 | M | 31 | 39.32834034 | JPN | control |
| DRR171638 | DRA008156 | F | 52 | 22.58270917 | JPN | control |
| DRR171639 | DRA008156 | F | 35 | 22.18934911 | JPN | control |
| DRR171640 | DRA008156 | M | 57 | 30.11621315 | JPN | control |
| DRR171641 | DRA008156 | F | 21 | 17.89802289 | JPN | control |
| DRR171642 | DRA008156 | F | 70 | 29.96432818 | JPN | control |
| DRR171643 | DRA008156 | M | 50 | 21.46193662 | JPN | control |
| DRR171644 | DRA008156 | F | 57 | 21.484375 | JPN | control |
| DRR171645 | DRA008156 | M | 52 | 27.63605442 | JPN | control |
| DRR171647 | DRA008156 | F | 51 | 21.90758246 | JPN | control |
| DRR171648 | DRA008156 | F | 61 | 17.087445 | JPN | control |
| DRR171649 | DRA008156 | F | 63 | 17.89802289 | JPN | control |
| DRR171650 | DRA008156 | M | 40 | 22.77318641 | JPN | control |
| DRR171651 | DRA008156 | M | 70 | 17.96875 | JPN | control |
| DRR171652 | DRA008156 | M | 27 | 24.96800974 | JPN | control |
| DRR171653 | DRA008156 | M | 34 | 21.92810713 | JPN | control |
| DRR171654 | DRA008156 | F | 75 | 30.21121884 | JPN | control |
| DRR171655 | DRA008156 | M | 75 | 21.30394858 | JPN | colorectal cancer |
| DRR171656 | DRA008156 | M | 37 | 22.12973973 | JPN | control |
| DRR171657 | DRA008156 | M | 72 | 24.91349481 | JPN | colorectal cancer |
| DRR171658 | DRA008156 | F | 49 | 19.28937927 | JPN | colorectal cancer |
| DRR171659 | DRA008156 | F | 52 | 17.90885536 | JPN | control |
| DRR171660 | DRA008156 | F | 55 | 21.08281329 | JPN | control |
| DRR171661 | DRA008156 | F | 71 | 21.92886277 | JPN | colorectal cancer |
| DRR171662 | DRA008156 | F | 48 | 29.51593861 | JPN | colorectal cancer |
| DRR171663 | DRA008156 | M | 43 | 21.63331531 | JPN | colorectal cancer |
| DRR171664 | DRA008156 | M | 60 | 19.1953028 | JPN | colorectal cancer |
| DRR171665 | DRA008156 | M | 51 | 22.22906193 | JPN | colorectal cancer |
| DRR171666 | DRA008156 | M | 69 | 22.75830678 | JPN | colorectal cancer |
| DRR171667 | DRA008156 | M | 69 | 25.68007405 | JPN | colorectal cancer |
| DRR171668 | DRA008156 | M | 76 | 21.92612582 | JPN | colorectal cancer |
| DRR171669 | DRA008156 | F | 64 | 25.33304989 | JPN | colorectal cancer |
| DRR171670 | DRA008156 | M | 78 | 23.30905307 | JPN | control |
| DRR171671 | DRA008156 | F | 64 | 29.67657562 | JPN | colorectal cancer |
| DRR171672 | DRA008156 | F | 63 | 23.55555556 | JPN | colorectal cancer |
| DRR171673 | DRA008156 | M | 73 | 22.89281998 | JPN | control |
| DRR171674 | DRA008156 | M | 71 | 21.0498179 | JPN | colorectal cancer |
| DRR171675 | DRA008156 | F | 72 | 20.32443826 | JPN | colorectal cancer |
| DRR171676 | DRA008156 | M | 53 | 31.23140988 | JPN | colorectal cancer |
| DRR171677 | DRA008156 | M | 64 | 26.25958475 | JPN | colorectal cancer |
| DRR171678 | DRA008156 | M | 59 | 28.06642387 | JPN | colorectal cancer |
| DRR171679 | DRA008156 | F | 68 | 25.29937595 | JPN | colorectal cancer |
| DRR171680 | DRA008156 | M | 68 | 23.4375 | JPN | colorectal cancer |
| DRR171681 | DRA008156 | M | 40 | 22.5981405 | JPN | colorectal cancer |
| DRR171682 | DRA008156 | F | 58 | 22.50692521 | JPN | colorectal cancer |
| DRR171683 | DRA008156 | M | 58 | 22.22906193 | JPN | control |
| DRR171684 | DRA008156 | M | 57 | 27.01685989 | JPN | colorectal cancer |
| DRR171685 | DRA008156 | M | 61 | 27.10204082 | JPN | colorectal cancer |
| DRR171686 | DRA008156 | F | 76 | 21.50188649 | JPN | control |
| DRR171687 | DRA008156 | F | 77 | 20.26935724 | JPN | colorectal cancer |
| DRR171688 | DRA008156 | M | 70 | 22.67995241 | JPN | control |
| DRR171689 | DRA008156 | M | 45 | 22.27530992 | JPN | colorectal cancer |
| DRR171690 | DRA008156 | M | 59 | 20.19946976 | JPN | colorectal cancer |
| DRR171691 | DRA008156 | F | 41 | 26.07897153 | JPN | control |
| DRR171692 | DRA008156 | M | 67 | 23.62444749 | JPN | colorectal cancer |
| DRR171693 | DRA008156 | M | 69 | 23.87511478 | JPN | colorectal cancer |
| DRR171694 | DRA008156 | F | 68 | 22.43230252 | JPN | colorectal cancer |
| DRR171695 | DRA008156 | M | 62 | 21.70792339 | JPN | control |
| DRR171696 | DRA008156 | F | 73 | 21.49028551 | JPN | colorectal cancer |
| DRR171697 | DRA008156 | F | 51 | 21.23057202 | JPN | control |
| DRR171698 | DRA008156 | M | 55 | 22.5981405 | JPN | colorectal cancer |
| DRR171699 | DRA008156 | F | 48 | 23.19109462 | JPN | colorectal cancer |
| DRR171701 | DRA008156 | M | 59 | 25.95155709 | JPN | colorectal cancer |
| DRR171702 | DRA008156 | F | 60 | 18.59012493 | JPN | control |
| DRR171704 | DRA008156 | F | 74 | 23.55555556 | JPN | colorectal cancer |
| DRR171705 | DRA008156 | M | 73 | 19.84126984 | JPN | colorectal cancer |
| DRR171706 | DRA008156 | F | 46 | 20.54569362 | JPN | colorectal cancer |
| DRR171707 | DRA008156 | F | 49 | 20.82999519 | JPN | control |
| DRR171708 | DRA008156 | M | 30 | NA | JPN | control |
| DRR171709 | DRA008156 | M | 56 | 24.09297052 | JPN | control |
| DRR171710 | DRA008156 | F | 77 | 21.92612582 | JPN | control |
| DRR171711 | DRA008156 | F | 64 | 21.09375 | JPN | control |
| DRR171712 | DRA008156 | M | 43 | 24.74093729 | JPN | control |
| DRR171713 | DRA008156 | F | 55 | 19.77769867 | JPN | control |
| DRR171714 | DRA008156 | F | 33 | 22.20633069 | JPN | control |
| DRR171715 | DRA008156 | F | 74 | 19.9099723 | JPN | control |
| DRR171716 | DRA008156 | M | 46 | 21.79944511 | JPN | control |
| DRR171717 | DRA008156 | M | 59 | 26.53375883 | JPN | control |
| DRR171718 | DRA008156 | F | 65 | 18.25631871 | JPN | control |
| DRR171719 | DRA008156 | M | 35 | 24.22145329 | JPN | control |
| DRR171720 | DRA008156 | F | 38 | 17.85062274 | JPN | control |
| DRR171721 | DRA008156 | M | 68 | 21.383942 | JPN | control |
| DRR171722 | DRA008156 | M | 60 | 22.22906193 | JPN | control |
| DRR171723 | DRA008156 | M | 66 | 24.6097337 | JPN | control |
| DRR171724 | DRA008156 | M | 72 | 25.20920136 | JPN | control |
| DRR171725 | DRA008156 | M | 47 | 23.56663223 | JPN | colorectal cancer |
| DRR171727 | DRA008156 | M | 66 | 22.05805119 | JPN | colorectal cancer |
| DRR171728 | DRA008156 | M | 45 | 20.61925365 | JPN | colorectal cancer |
| DRR171729 | DRA008156 | F | 63 | 21.36752137 | JPN | colorectal cancer |
| DRR171730 | DRA008156 | F | 75 | 18.36547291 | JPN | control |
| DRR171731 | DRA008156 | F | 68 | 21.78649237 | JPN | colorectal cancer |
| DRR171732 | DRA008156 | M | 66 | 24.67550027 | JPN | colorectal cancer |
| DRR171734 | DRA008156 | F | 55 | 23.55555556 | JPN | control |
| DRR171735 | DRA008156 | M | 76 | 19.17 | JPN | colorectal cancer |
| DRR171736 | DRA008156 | M | 73 | 22.05805119 | JPN | colorectal cancer |
| DRR171737 | DRA008156 | F | 51 | 18.82711104 | JPN | colorectal cancer |
| DRR171738 | DRA008156 | F | 78 | 24.03440715 | JPN | control |
| DRR171739 | DRA008156 | F | 67 | 24.32322868 | JPN | colorectal cancer |
| DRR171740 | DRA008156 | M | 69 | 22.75830678 | JPN | colorectal cancer |
| DRR171741 | DRA008156 | M | 52 | 31.02040816 | JPN | colorectal cancer |
| DRR171742 | DRA008156 | M | 50 | 24.81632653 | JPN | control |
| DRR171743 | DRA008156 | F | 63 | 36.5230095 | JPN | colorectal cancer |
| DRR171744 | DRA008156 | M | 65 | 24.28097534 | JPN | control |
| DRR171745 | DRA008156 | F | 64 | 22.47658689 | JPN | colorectal cancer |
| DRR171746 | DRA008156 | F | 73 | 16.88019391 | JPN | colorectal cancer |
| DRR171747 | DRA008156 | M | 73 | 23.33546614 | JPN | colorectal cancer |
| DRR171750 | DRA008156 | F | 60 | 20.82999519 | JPN | colorectal cancer |
| DRR171752 | DRA008156 | M | 61 | 26.2335558 | JPN | control |
| DRR171754 | DRA008156 | M | 71 | 26.21882086 | JPN | colorectal cancer |
| DRR171755 | DRA008156 | M | 67 | 23.59700421 | JPN | colorectal cancer |
| DRR171756 | DRA008156 | M | 65 | 21.71806609 | JPN | colorectal cancer |
| DRR171757 | DRA008156 | M | 64 | 23.05175491 | JPN | colorectal cancer |
| DRR171758 | DRA008156 | M | 60 | 27.06405159 | JPN | colorectal cancer |
| DRR171759 | DRA008156 | M | 71 | 25.15589569 | JPN | control |
| DRR171760 | DRA008156 | M | 77 | 24.609375 | JPN | control |
| DRR171761 | DRA008156 | M | 49 | 26.12244898 | JPN | colorectal cancer |
| DRR171762 | DRA008156 | F | 78 | 20.42941836 | JPN | control |
| DRR171763 | DRA008156 | M | 69 | 19.43301326 | JPN | colorectal cancer |
| DRR171764 | DRA008156 | F | 49 | 22.64086462 | JPN | colorectal cancer |
| DRR171765 | DRA008156 | F | 69 | 20.613131 | JPN | colorectal cancer |
| DRR171766 | DRA008156 | M | 70 | 26.76977989 | JPN | colorectal cancer |
| DRR171767 | DRA008156 | M | 68 | 23.1206236 | JPN | control |
| DRR171768 | DRA008156 | F | 77 | 22.85714286 | JPN | control |
| DRR171769 | DRA008156 | F | 60 | 21.11090261 | JPN | control |
| DRR171770 | DRA008156 | F | 71 | 24.609375 | JPN | control |
| DRR171771 | DRA008156 | F | 61 | 24.609375 | JPN | colorectal cancer |
| DRR171773 | DRA008156 | M | 57 | 25.43268596 | JPN | colorectal cancer |
| DRR171774 | DRA008156 | F | 58 | 22.54657648 | JPN | control |
| DRR171775 | DRA008156 | F | 73 | 26.57312925 | JPN | colorectal cancer |
| DRR171776 | DRA008156 | F | 62 | 19.921875 | JPN | control |
| DRR171777 | DRA008156 | M | 62 | 23.95122659 | JPN | colorectal cancer |
| DRR171778 | DRA008156 | M | 47 | 21.4532872 | JPN | colorectal cancer |
| DRR171779 | DRA008156 | M | 47 | 20.67901235 | JPN | control |
| DRR171780 | DRA008156 | M | 42 | 33.14 | JPN | control |
| DRR171781 | DRA008156 | F | 63 | 20.13477975 | JPN | colorectal cancer |
| DRR171782 | DRA008156 | M | 73 | 24.6097337 | JPN | colorectal cancer |
| DRR171783 | DRA008156 | M | 74 | 25.05930703 | JPN | control |
| DRR171786 | DRA008156 | F | 73 | 19.56295525 | JPN | colorectal cancer |
| DRR171787 | DRA008156 | F | 61 | 21.64127424 | JPN | colorectal cancer |
| DRR171788 | DRA008156 | F | 76 | 19.11111111 | JPN | control |
| DRR171789 | DRA008156 | M | 76 | 24.65303141 | JPN | colorectal cancer |
| DRR171790 | DRA008156 | F | 47 | 22.37567995 | JPN | control |
| DRR171791 | DRA008156 | M | 52 | 23.25501864 | JPN | colorectal cancer |
| DRR171792 | DRA008156 | M | 69 | 16.14152893 | JPN | colorectal cancer |
| DRR171793 | DRA008156 | M | 71 | 22.64737696 | JPN | control |
| DRR171794 | DRA008156 | M | 53 | 20.76124567 | JPN | colorectal cancer |
| DRR171795 | DRA008156 | M | 68 | 23.7953599 | JPN | control |
| DRR171796 | DRA008156 | M | 70 | 21.04804761 | JPN | colorectal cancer |
| DRR171797 | DRA008156 | M | 59 | 24.84098009 | JPN | colorectal cancer |
| DRR171798 | DRA008156 | M | 71 | 22.94213045 | JPN | control |
| DRR171799 | DRA008156 | M | 74 | 20.61925365 | JPN | control |
| DRR171802 | DRA008156 | M | 63 | 24.85907356 | JPN | colorectal cancer |
| DRR171803 | DRA008156 | M | 69 | 25.72755521 | JPN | colorectal cancer |
| DRR171804 | DRA008156 | M | 52 | 21.30394858 | JPN | control |
| DRR171807 | DRA008156 | M | 78 | 21.09375 | JPN | control |
| DRR171809 | DRA008156 | F | 77 | 21.28742654 | JPN | control |
| DRR171810 | DRA008156 | F | 59 | 22.22222222 | JPN | control |
| DRR171811 | DRA008156 | M | 50 | 23.49 | JPN | colorectal cancer |
| DRR171812 | DRA008156 | M | 68 | 23.87511478 | JPN | colorectal cancer |
| DRR171813 | DRA008156 | M | 51 | 29.04370899 | JPN | control |
| DRR171814 | DRA008156 | M | 54 | 23.71184463 | JPN | control |
| DRR171815 | DRA008156 | M | 65 | 25.05930703 | JPN | colorectal cancer |
| DRR171816 | DRA008156 | M | 60 | 23.22543185 | JPN | colorectal cancer |
| DRR171817 | DRA008156 | F | 71 | 31.11111111 | JPN | colorectal cancer |
| DRR171818 | DRA008156 | M | 55 | 26.81221871 | JPN | control |
| DRR171819 | DRA008156 | F | 66 | 20.42941836 | JPN | colorectal cancer |
| DRR171821 | DRA008156 | F | 74 | 25.73 | JPN | colorectal cancer |
| DRR171822 | DRA008156 | F | 41 | 20.32443826 | JPN | control |
| DRR171824 | DRA008156 | M | 72 | 22.03856749 | JPN | colorectal cancer |
| DRR171826 | DRA008156 | M | 78 | 22.15102251 | JPN | control |
| DRR171828 | DRA008156 | M | 51 | 18.16620425 | JPN | colorectal cancer |
| DRR171829 | DRA008156 | F | 65 | 17.99307958 | JPN | colorectal cancer |
| DRR171830 | DRA008156 | M | 67 | 21.45357371 | JPN | control |
| DRR171831 | DRA008156 | M | 61 | 25.95155709 | JPN | control |
| DRR171835 | DRA008156 | F | 61 | 27.00513097 | JPN | control |
| DRR171836 | DRA008156 | M | 51 | 22.72440348 | JPN | colorectal cancer |
| DRR171837 | DRA008156 | F | 66 | 31.62421994 | JPN | control |
| DRR171838 | DRA008156 | F | 75 | 18.97453196 | JPN | colorectal cancer |
| DRR171839 | DRA008156 | F | 62 | 18.19548277 | JPN | control |
| DRR171840 | DRA008156 | M | 57 | 23.24380165 | JPN | control |
| DRR171841 | DRA008156 | F | 70 | 23.14049587 | JPN | colorectal cancer |
| DRR171842 | DRA008156 | M | 73 | 24.30248706 | JPN | control |
| DRR171843 | DRA008156 | M | 71 | 22.48132907 | JPN | colorectal cancer |
| DRR171844 | DRA008156 | M | 47 | 35.20104514 | JPN | colorectal cancer |
| DRR171845 | DRA008156 | M | 66 | 18.71094925 | JPN | colorectal cancer |
| DRR171846 | DRA008156 | F | 68 | 19.50059453 | JPN | control |
| DRR171847 | DRA008156 | F | 74 | 24.34960912 | JPN | control |
| DRR171848 | DRA008156 | M | 64 | 23.83673469 | JPN | colorectal cancer |
| DRR171849 | DRA008156 | M | 74 | 20.703125 | JPN | control |
| DRR171850 | DRA008156 | M | 65 | 27.18089991 | JPN | colorectal cancer |
| DRR171852 | DRA008156 | M | 63 | 20.89795918 | JPN | control |
| DRR171853 | DRA008156 | M | 60 | 20.80856124 | JPN | control |
| DRR171855 | DRA008156 | M | 77 | 23.33768443 | JPN | colorectal cancer |
| DRR171856 | DRA008156 | F | 67 | 25.43748081 | JPN | colorectal cancer |
| DRR171857 | DRA008156 | M | 74 | 25.95155709 | JPN | colorectal cancer |
| DRR171858 | DRA008156 | M | 69 | 19.36247266 | JPN | colorectal cancer |
| DRR171859 | DRA008156 | F | 61 | 18.59103675 | JPN | control |
| DRR171860 | DRA008156 | F | 54 | 22.18934911 | JPN | control |
| DRR171861 | DRA008156 | F | 54 | 19.6282647 | JPN | control |
| DRR171862 | DRA008156 | F | 74 | 20.83 | JPN | control |
| DRR171863 | DRA008156 | M | 78 | 29.7210791 | JPN | colorectal cancer |
| DRR171864 | DRA008156 | F | 70 | 23.06805075 | JPN | colorectal cancer |
| DRR171866 | DRA008156 | M | 73 | 22.91303307 | JPN | colorectal cancer |
| DRR171867 | DRA008156 | F | 75 | 21.17021756 | JPN | control |
| DRR171868 | DRA008156 | M | 58 | 19.605192 | JPN | colorectal cancer |
| DRR171869 | DRA008156 | M | 71 | 23.38435374 | JPN | control |
| DRR171870 | DRA008156 | F | 56 | 23.01117686 | JPN | colorectal cancer |
| DRR171871 | DRA008156 | M | 69 | 22.83950617 | JPN | colorectal cancer |
| DRR171872 | DRA008156 | M | 52 | 23.66524436 | JPN | colorectal cancer |
| DRR171873 | DRA008156 | M | 69 | 22.14532872 | JPN | control |
| DRR171874 | DRA008156 | F | 65 | 24.91990032 | JPN | colorectal cancer |
| DRR171875 | DRA008156 | F | 63 | 19.81784449 | JPN | colorectal cancer |
| DRR171876 | DRA008156 | M | 49 | 37.55102041 | JPN | colorectal cancer |
| DRR171877 | DRA008156 | M | 63 | 19.75308642 | JPN | colorectal cancer |
| DRR171878 | DRA008156 | M | 39 | 19.37919743 | JPN | colorectal cancer |
| DRR171879 | DRA008156 | F | 67 | 21.45726808 | JPN | colorectal cancer |
| DRR171881 | DRA008156 | F | 69 | 19.77769867 | JPN | colorectal cancer |
| DRR171882 | DRA008156 | M | 50 | 22.05219018 | JPN | colorectal cancer |
| DRR171883 | DRA008156 | F | 73 | 20.69049454 | JPN | control |
| DRR171884 | DRA008156 | F | 73 | 18.31425598 | JPN | colorectal cancer |
| DRR171887 | DRA008156 | M | 69 | 24.28097534 | JPN | control |
| DRR171888 | DRA008156 | M | 64 | 20.44674202 | JPN | colorectal cancer |
| DRR171889 | DRA008156 | M | 55 | 26.17519452 | JPN | colorectal cancer |
| DRR171890 | DRA008156 | M | 53 | 26.36560303 | JPN | colorectal cancer |
| DRR171893 | DRA008156 | M | 64 | 22.67995241 | JPN | colorectal cancer |
| DRR171894 | DRA008156 | M | 76 | 20.79672989 | JPN | control |
| DRR171895 | DRA008156 | M | 68 | 24.76756592 | JPN | colorectal cancer |
| DRR171896 | DRA008156 | F | 46 | 19.95935549 | JPN | control |
| DRR171897 | DRA008156 | F | 47 | 18.06616734 | JPN | colorectal cancer |
| DRR171898 | DRA008156 | M | 77 | 24.6097337 | JPN | colorectal cancer |
| DRR171899 | DRA008156 | M | 70 | 24.22145329 | JPN | control |
| DRR171901 | DRA008156 | M | 72 | 24.69040546 | JPN | colorectal cancer |
| DRR171902 | DRA008156 | M | 60 | 23.42355741 | JPN | colorectal cancer |
| DRR171903 | DRA008156 | M | 73 | 23.30668005 | JPN | colorectal cancer |
| DRR171904 | DRA008156 | M | 60 | 21.29529475 | JPN | colorectal cancer |
| DRR171906 | DRA008156 | F | 59 | 20.504934 | JPN | colorectal cancer |
| DRR171907 | DRA008156 | M | 59 | 25.05930703 | JPN | colorectal cancer |
| DRR171908 | DRA008156 | F | 61 | 23.61275089 | JPN | colorectal cancer |
| DRR171909 | DRA008156 | F | 62 | 22.03172568 | JPN | colorectal cancer |
| DRR171912 | DRA008156 | F | 65 | 24.32322868 | JPN | control |
| DRR171913 | DRA008156 | M | 52 | 26.42356982 | JPN | colorectal cancer |
| DRR171914 | DRA008156 | F | 64 | 21.1552942 | JPN | control |
| DRR171915 | DRA008156 | F | 56 | 17.4817898 | JPN | colorectal cancer |
| DRR171916 | DRA008156 | M | 77 | 24.4646016 | JPN | control |
| DRR171917 | DRA008156 | F | 54 | 24.74745426 | JPN | colorectal cancer |
| DRR171918 | DRA008156 | M | 65 | 25.39343112 | JPN | control |
| DRR171919 | DRA008156 | M | 70 | 29.27099403 | JPN | control |
| DRR171920 | DRA008156 | M | 70 | 22.0603538 | JPN | control |
| DRR171921 | DRA008156 | F | 76 | 29.01307967 | JPN | colorectal cancer |
| DRR171922 | DRA008156 | F | 49 | 19.6311176 | JPN | control |
| DRR171925 | DRA008156 | F | 62 | 22.64086462 | JPN | control |
| DRR171927 | DRA008156 | F | 67 | 19.67516685 | JPN | control |
| DRR171928 | DRA008156 | M | 69 | 24.39105884 | JPN | control |
| DRR171930 | DRA008156 | F | 76 | 24.97398543 | JPN | control |
| DRR171931 | DRA008156 | M | 68 | 22.83737024 | JPN | control |
| DRR171932 | DRA008156 | F | 69 | 22.95908766 | JPN | control |
| DRR171933 | DRA008156 | F | 62 | 19.05197378 | JPN | control |
| DRR171935 | DRA008156 | F | 66 | 19.55555556 | JPN | control |
| DRR171936 | DRA008156 | F | 72 | 22.37034332 | JPN | control |
| DRR171938 | DRA008156 | M | 76 | 23.828125 | JPN | control |
| DRR171939 | DRA008156 | F | 45 | 19.22768787 | JPN | control |
| DRR171940 | DRA008156 | M | 66 | 23.4375 | JPN | control |
| DRR171941 | DRA008156 | M | 72 | 20.90419501 | JPN | colorectal cancer |
| DRR171942 | DRA008156 | F | 57 | 24.45606342 | JPN | colorectal cancer |
| DRR171943 | DRA008156 | M | 71 | 27.34375 | JPN | colorectal cancer |
| DRR171944 | DRA008156 | F | 73 | 22.15102251 | JPN | colorectal cancer |
| DRR171945 | DRA008156 | M | 74 | 19.33372992 | JPN | control |
| DRR171946 | DRA008156 | M | 57 | 26.8809517 | JPN | colorectal cancer |
| DRR171947 | DRA008156 | M | 65 | 26.5625 | JPN | control |
| DRR171948 | DRA008156 | F | 66 | 19.06771066 | JPN | control |
| DRR171949 | DRA008156 | F | 66 | 21.64127424 | JPN | control |
| DRR171950 | DRA008156 | M | 59 | 28.84153181 | JPN | control |
| DRR171951 | DRA008156 | M | 65 | 23.66524436 | JPN | control |
| DRR171952 | DRA008156 | M | 73 | 22.32142857 | JPN | colorectal cancer |
| DRR171954 | DRA008156 | M | 40 | 25 | JPN | control |
| DRR171955 | DRA008156 | F | 71 | 29.96432818 | JPN | colorectal cancer |
| DRR171956 | DRA008156 | F | 67 | 20.17325264 | JPN | control |
| DRR171957 | DRA008156 | M | 61 | 22.22906193 | JPN | colorectal cancer |
| DRR171958 | DRA008156 | F | 64 | 24.21875 | JPN | colorectal cancer |
| DRR171959 | DRA008156 | M | 65 | 21.8299522 | JPN | colorectal cancer |
| DRR171960 | DRA008156 | F | 70 | 24.73246136 | JPN | colorectal cancer |
| DRR171961 | DRA008156 | F | 79 | 24.44444444 | JPN | control |
| DRR171962 | DRA008156 | M | 70 | 29.76083761 | JPN | control |
| DRR171963 | DRA008156 | F | 62 | 21.875 | JPN | colorectal cancer |
| DRR171464 | DRA008156 | M | 50 | 23.67125363 | JPN | adenoma |
| DRR171468 | DRA008156 | M | 78 | 23.03004535 | JPN | adenoma |
| DRR171470 | DRA008156 | M | 44 | 22.12973973 | JPN | adenoma |
| DRR171473 | DRA008156 | M | 64 | 24.15881797 | JPN | adenoma |
| DRR171475 | DRA008156 | M | 64 | 18.77834467 | JPN | adenoma |
| DRR171476 | DRA008156 | M | 71 | 18.359375 | JPN | adenoma |
| DRR171478 | DRA008156 | F | 65 | 20.42941836 | JPN | adenoma |
| DRR171480 | DRA008156 | M | 52 | 22.58270917 | JPN | adenoma |
| DRR171481 | DRA008156 | F | 72 | 19.31295201 | JPN | adenoma |
| DRR171482 | DRA008156 | M | 53 | 23.38435374 | JPN | adenoma |
| DRR171486 | DRA008156 | M | 58 | 25.40281608 | JPN | adenoma |
| DRR171495 | DRA008156 | M | 72 | 49.53513184 | JPN | adenoma |
| DRR171496 | DRA008156 | M | 77 | 19.48696145 | JPN | adenoma |
| DRR171498 | DRA008156 | M | 55 | 25.2493372 | JPN | adenoma |
| DRR171505 | DRA008156 | M | 58 | 22.85714286 | JPN | adenoma |
| DRR171577 | DRA008156 | F | 73 | 16.65249433 | JPN | adenoma |
| DRR171583 | DRA008156 | F | 70 | 18.7961895 | JPN | adenoma |
| DRR171590 | DRA008156 | F | 41 | 20.88888889 | JPN | adenoma |
| DRR171611 | DRA008156 | M | 65 | 19.72318339 | JPN | adenoma |
| DRR171612 | DRA008156 | M | 62 | 24.96800974 | JPN | adenoma |
| DRR171614 | DRA008156 | F | 74 | 20.13477975 | JPN | adenoma |
| DRR171615 | DRA008156 | F | 57 | 19.36849691 | JPN | adenoma |
| DRR171632 | DRA008156 | F | 63 | 21.22788762 | JPN | adenoma |
| DRR171633 | DRA008156 | F | 64 | 19.81784449 | JPN | adenoma |
| DRR171635 | DRA008156 | M | 72 | 22.12973973 | JPN | adenoma |
| DRR171638 | DRA008156 | F | 47 | 30.757401 | JPN | adenoma |
| DRR171649 | DRA008156 | M | 67 | 25.39343112 | JPN | adenoma |
| DRR171729 | DRA008156 | M | 60 | 28.40909091 | JPN | adenoma |
| DRR171736 | DRA008156 | F | 67 | 22.0603538 | JPN | adenoma |
| DRR171751 | DRA008156 | F | 74 | 19.14672216 | JPN | adenoma |
| DRR171752 | DRA008156 | M | 78 | 27.34375 | JPN | adenoma |
| DRR171754 | DRA008156 | M | 58 | 21.95247934 | JPN | adenoma |
| DRR171756 | DRA008156 | M | 51 | 21.79820239 | JPN | adenoma |
| DRR171775 | DRA008156 | M | 67 | 23.14725512 | JPN | adenoma |
| DRR171787 | DRA008156 | M | 69 | 26.171875 | JPN | adenoma |
| DRR171788 | DRA008156 | M | 52 | 22.5710475 | JPN | adenoma |
| DRR171803 | DRA008156 | M | 66 | 18.37682515 | JPN | adenoma |
| DRR171804 | DRA008156 | M | 75 | 21.45357371 | JPN | adenoma |
| DRR171808 | DRA008156 | M | 73 | 21.0076678 | JPN | adenoma |
| DRR171809 | DRA008156 | M | 74 | 28.68514468 | JPN | adenoma |
| DRR171811 | DRA008156 | M | 72 | 23.66524436 | JPN | adenoma |
| DRR171823 | DRA008156 | M | 71 | 21.1552942 | JPN | adenoma |
| DRR171826 | DRA008156 | M | 58 | 23.87511478 | JPN | adenoma |
| DRR171828 | DRA008156 | F | 72 | 17.14677641 | JPN | adenoma |
| DRR171830 | DRA008156 | M | 73 | 21.79930796 | JPN | adenoma |
| DRR171835 | DRA008156 | M | 59 | 24.39105884 | JPN | adenoma |
| DRR171836 | DRA008156 | M | 65 | 24.85907356 | JPN | adenoma |
| DRR171837 | DRA008156 | M | 64 | 22.40587695 | JPN | adenoma |
| DRR171854 | DRA008156 | M | 59 | 22.39541342 | JPN | adenoma |
| DRR171857 | DRA008156 | M | 70 | 20.90419501 | JPN | adenoma |
| DRR171868 | DRA008156 | M | 74 | 23.05175491 | JPN | adenoma |
| DRR171883 | DRA008156 | M | 63 | 23.59700421 | JPN | adenoma |
| DRR171888 | DRA008156 | M | 52 | 21.04804761 | JPN | adenoma |
| DRR171889 | DRA008156 | M | 60 | 25.95155709 | JPN | adenoma |
| DRR171894 | DRA008156 | M | 53 | 27.99036281 | JPN | adenoma |
| DRR171895 | DRA008156 | M | 58 | 21.97133586 | JPN | adenoma |
| DRR171903 | DRA008156 | M | 69 | 25.01352082 | JPN | adenoma |
| DRR171908 | DRA008156 | M | 63 | 20.14797199 | JPN | adenoma |
| DRR171913 | DRA008156 | F | 66 | 20.23950076 | JPN | adenoma |
| DRR171914 | DRA008156 | M | 48 | 19.10009183 | JPN | adenoma |
| DRR171926 | DRA008156 | F | 64 | 31.11111111 | JPN | adenoma |
| DRR171927 | DRA008156 | F | 58 | 34.80874965 | JPN | adenoma |
| DRR171929 | DRA008156 | M | 64 | 21.79930796 | JPN | adenoma |
| DRR171932 | DRA008156 | F | 70 | 25.5367036 | JPN | adenoma |
| DRR171937 | DRA008156 | F | 59 | 20.06095444 | JPN | adenoma |
| DRR171940 | DRA008156 | M | 54 | 22.40817899 | JPN | adenoma |
| DRR171956 | DRA008156 | F | 41 | 19.921875 | JPN | adenoma |

##### Table S10. Topology species detected in public cohorts.

| **Species** | **Topology group** |
| --- | --- |
| *Anaerostipes caccae* | T1 |
| *Blautia caecimuris* | T1 |
| *Blautia hansenii* | T1 |
| *Blautia producta* | T1 |
| *Clostridium symbiosum* | T1 |
| *Enterobacter hormaechei* | T1 |
| *Enterobacter roggenkampii* | T1 |
| *Enterobacter kobei* | T1 |
| *Erysipelatoclostridium ramosum* | T1 |
| *Faecalimonas umbilicata* | T1 |
| *Hungatella hathewayi* | T1 |
| *Klebsiella variicola* | T1 |
| *Tyzzerella nexilis* | T1 |
| *Anaerosacchariphilus sp NSJ 68* | T2 |
| *Blautia stercoris* | T2 |
| *Clostridiaceae bacterium Marseille Q4143* | T2 |
| *Clostridiaceae bacterium Marseille Q4145* | T2 |
| *Clostridiaceae unclassified SGB4771* | T2 |
| *Clostridium SGB4751* | T2 |
| *Clostridium sp AF12 28* | T2 |
| *Clostridium sp AF32 12BH* | T2 |
| *Coprococcus catus* | T2 |
| *Dorea longicatena* | T2 |
| *Faecalibacterium SGB15346* | T2 |
| *Faecalicatena fissicatena* | T2 |
| *GGB13493 SGB15238* | T2 |
| *GGB34228 SGB72916* | T2 |
| *GGB51884 SGB49168* | T2 |
| *GGB58485 SGB80143* | T2 |
| *GGB9302 SGB14263* | T2 |
| *GGB9342 SGB14306* | T2 |
| *GGB9614 SGB15049* | T2 |
| *GGB9616 SGB15052* | T2 |
| *GGB9634 SGB15093* | T2 |
| *GGB9758 SGB15368* | T2 |
| *Lachnospira sp NSJ 43* | T2 |
| *Lachnospiraceae bacterium AM48 27BH* | T2 |
| *Oscillibacter sp ER4* | T2 |
| *Oscillospiraceae bacterium Marseille Q3528* | T2 |
| *Oscillospiraceae bacterium* | T2 |

**Table S11. Associations between T-score and plasma metabolite profiles. Related to Figure 5.**

| **Topology** | **Plasma metabolite** | **Spearman's correlation** | **FDR** |
| --- | --- | --- | --- |
| T-score | Sulfolithocholylglycine | -0.55 | <0.001 |
| T-score | Isoursodeoxycholic acid | 0.50 | <0.001 |
| T-score | Glycoursodeoxycholate-3-sulfate | 0.45 | <0.001 |
| T-score | Deoxycholic acid glycine conjugate | -0.44 | <0.001 |
| T-score | Glycodeoxycholate-3-sulfate | -0.42 | <0.001 |
| T-score | Taurodeoxycholic acid | -0.40 | <0.001 |
| T-score | Ursodeoxycholic acid | 0.31 | <0.001 |
| T-score | Hyodeoxycholic acid | 0.31 | <0.001 |
| T-score | Indole-3-propionic acid | -0.31 | <0.001 |
| T-score | Glycoursodeoxycholic acid | 0.30 | <0.001 |
| T-score | 4-ethylphenyl sulfate | -0.28 | <0.001 |
| T-score | Tauroursodeoxycholic acid | 0.26 | <0.001 |
| T-score | Leu-Glu | 0.23 | <0.001 |
| T-score | Tetradecanedioic acid(FFA(14:0-DC)) | -0.21 | <0.001 |
| T-score | Uracil | -0.20 | <0.001 |
| T-score | alpha-Ketoisovaleric acid | 0.20 | <0.001 |
| T-score | LysoPC(0:0/16:1) | 0.20 | <0.001 |
| T-score | p-Cresol sulfate | -0.19 | <0.001 |
| T-score | LysoPC(22:4/0:0) | 0.19 | <0.001 |
| T-score | LysoPC(16:1/0:0) | 0.19 | <0.001 |
| T-score | Nonanoylcarnitine(AcCa(9:0)) | -0.19 | <0.001 |
| T-score | 3b-Hydroxy-5-cholenoic acid glycine conjugate | -0.18 | 0.001 |
| T-score | LysoPE(18:1/0:0) | 0.18 | 0.001 |
| T-score | N-Formyl-L-methionine | 0.18 | 0.001 |
| T-score | Isobutyrylglycine/N-Butyrylglycine | 0.18 | 0.001 |
| T-score | LysoPC(18:1/0:0) | 0.18 | 0.001 |
| T-score | Ribonic acid/Xylonate | -0.18 | 0.001 |
| T-score | Cholic acid+Hyocholic acid+Ursocholic acid | -0.17 | 0.001 |
| T-score | LysoPE(0:0/18:1) | 0.17 | 0.001 |
| T-score | Behenic acid(FFA(22:0)) | -0.17 | 0.001 |
| T-score | Urea | -0.17 | 0.001 |
| T-score | 5alpha-Androstane-3beta,17beta-diol disulfate | 0.17 | 0.002 |
| T-score | Tridecanoyl carnitine(AcCa(13:0)) | -0.17 | 0.002 |
| T-score | LysoPE(22:5/0:0) | 0.17 | 0.002 |
| T-score | Gluconic acid | -0.16 | 0.002 |
| T-score | Ascorbic acid | 0.16 | 0.003 |
| T-score | Proline betaine | -0.16 | 0.003 |
| T-score | LysoPC(0:0/18:1) | 0.16 | 0.003 |
| T-score | 5alpha-Pregnan-3beta,20alpha-diol disulfate | 0.16 | 0.003 |
| T-score | Glucuronic acid | -0.16 | 0.003 |
| T-score | LysoPC(22:5/0:0) | 0.16 | 0.004 |
| T-score | 4-Hydroxyproline | -0.16 | 0.004 |
| T-score | L-2-Hydroxyglutaric acid | -0.15 | 0.004 |
| T-score | gamma-Glu-Phe | 0.15 | 0.005 |
| T-score | Palmitoleic acid(FFA(16:1)) | 0.15 | 0.005 |
| T-score | N,N,N-trimethyl-5-aminovaleric acid | -0.15 | 0.005 |
| T-score | LysoPC(17:0/0:0) | -0.15 | 0.005 |
| T-score | Galactaric acid/Glucaric acid | -0.15 | 0.005 |
| T-score | 2-Hydroxy-3-methylbutyric acid | 0.15 | 0.005 |
| T-score | LysoPC(0:0/22:4) | 0.15 | 0.005 |

FDR: false discovery rate.

**Table S12. Pooled associations of 50 plasma metabolites with the T-score in multivariable linear regression analyses.**

| **Plasma metabolite#** | **Pooled coefficient (*10^-3)** | **95% CI (*10^-3)** | **Pooled *P* value*** | **I^2^ (%)** | ***P* heterogeneity** |
| --- | --- | --- | --- | --- | --- |
| Isoursodeoxycholic acid | 23.71 | 19.43 – 27.99 | <0.001 | 0.00 | 0.497 |
| Sulfolithocholylglycine | -28.54 | -34.24 – -22.85 | <0.001 | 13.97 | 0.299 |
| Deoxycholic acid glycine conjugate | -28.49 | -34.92 – -22.05 | <0.001 | 0.00 | 0.535 |
| Glycoursodeoxycholic acid | 19.93 | 15.08 – 24.77 | <0.001 | 6.30 | 0.185 |
| Glycodeoxycholate-3-sulfate | -19.97 | -25.2 – -14.73 | <0.001 | 18.97 | 0.463 |
| Taurodeoxycholic acid | -14.72 | -18.71 – -10.74 | <0.001 | 0.00 | 0.606 |
| 4-ethylphenyl sulfate | -20.82 | -27.5 – -14.14 | <0.001 | 0.00 | 0.661 |
| Tetradecanedioic acid(FFA(14:0-DC) | -8.34 | -11.12 – -5.55 | <0.001 | 0.00 | 0.652 |
| Indole-3-propionic acid | -17.84 | -23.88 – -11.8 | <0.001 | 0.00 | 0.818 |
| Glycoursodeoxycholate-3-sulfate | 18.58 | 11.8 – 25.36 | <0.001 | 65.29 | 0.020 |
| Hyodeoxycholic acid | 14.26 | 7.7 – 20.83 | <0.001 | 57.27 | 0.080 |
| Ursodeoxycholic acid | 14.21 | 7.5 – 20.92 | <0.001 | 58.48 | 0.074 |
| LysoPC(16:1/0:0) | 2.63 | 1.17 – 4.09 | <0.001 | 0.00 | 0.299 |
| 2-Hydroxy-3-methylbutyric acid | 4.03 | 1.59 – 6.48 | 0.001 | 0.00 | 0.337 |
| LysoPE(22:5/0:0) | 2.23 | 0.88 – 3.58 | 0.001 | 0.00 | 0.675 |
| Tauroursodeoxycholic acid | 6.91 | 0.72 – 13.1 | 0.029 | 77.24 | 0.009 |
| p-Cresol sulfate | -4.58 | -9.21 – 0.04 | 0.052 | 0.00 | 0.084 |
| Leu-Glu | 1.93 | -0.14 – 4.00 | 0.068 | 0.00 | 0.743 |
| N-Formyl-L-methionine | 1.41 | -0.23 – 3.04 | 0.092 | 0.01 | 0.519 |
| Palmitoleic acid(FFA(16:1) | 1.53 | -0.33 – 3.38 | 0.106 | 0.00 | 0.744 |
| gamma-Glu-Phe | 0.80 | -0.19 – 1.79 | 0.113 | 0.00 | 0.987 |
| LysoPC(22:4/0:0) | 1.20 | -0.32 – 2.72 | 0.121 | 0.00 | 0.611 |
| LysoPC(22:5/0:0) | 1.84 | -0.56 – 4.25 | 0.132 | 56.38 | 0.089 |
| Isobutyrylglycine/N-Butyrylglycine | -0.96 | -2.34 – 0.42 | 0.174 | 0.00 | 0.886 |
| 3b-Hydroxy-5-cholenoic acid glycine conjugate | -1.12 | -2.76 – 0.53 | 0.185 | 0.00 | 0.830 |
| Cholic acid+Hyocholic acid+Ursocholic acid | -1.16 | -3.17 – 0.84 | 0.256 | 0.00 | 0.207 |
| Gluconic acid | 2.30 | -1.89 – 6.49 | 0.282 | 83.88 | 0.007 |
| Tridecanoyl carnitine(AcCa(13:0) | -1.50 | -4.34 – 1.35 | 0.303 | 0.00 | 0.617 |
| Glucuronic acid | 0.97 | -1.00 – 2.94 | 0.335 | 31.17 | 0.310 |
| L-2-Hydroxyglutaric acid | -0.51 | -1.68 – 0.65 | 0.386 | 45.89 | 0.123 |
| Galactaric acid/Glucaric acid | -0.74 | -2.42 – 0.95 | 0.391 | 0.00 | 0.871 |
| LysoPE(18:1/0:0) | 0.69 | -0.82 – 2.20 | 0.369 | 0.01 | 0.349 |
| LysoPC(18:1/0:0) | 0.65 | -0.75 – 2.05 | 0.363 | 29.28 | 0.368 |
| LysoPC(0:0/22:4) | 0.49 | -0.68 – 1.67 | 0.413 | 0.01 | 0.771 |
| Ribonic acid/Xylonate | 0.44 | -0.86 – 1.73 | 0.511 | 0.02 | 0.548 |
| 5alpha-Pregnan-3beta,20alpha-diol disulfate | 0.98 | -1.95 – 3.90 | 0.513 | 51.09 | 0.082 |
| LysoPC(0:0/16:1) | 1.85 | -3.4 – 7.11 | 0.489 | 49.15 | 0.166 |
| Uracil | -0.31 | -1.27 – 0.66 | 0.536 | 0.02 | 0.513 |
| Proline betaine | -1.63 | -7.35 – 4.10 | 0.578 | 6.67 | 0.358 |
| Behenic acid(FFA(22:0) | -0.56 | -2.63 – 1.51 | 0.597 | 0.00 | 0.996 |
| LysoPE(0:0/18:1) | 0.32 | -0.86 – 1.51 | 0.594 | 0.01 | 0.246 |
| LysoPC(0:0/18:1) | 0.38 | -0.99 – 1.76 | 0.584 | 35.40 | 0.289 |
| 5alpha-Androstane-3beta,17beta-diol disulfate | 1.50 | -5.17 – 8.18 | 0.659 | 74.13 | 0.006 |
| LysoPC(17:0/0:0) | -0.32 | -1.98 – 1.35 | 0.710 | 23.07 | 0.296 |
| alpha-Ketoisovaleric acid | 0.16 | -0.92 – 1.24 | 0.769 | 0.02 | 0.605 |
| Urea | -0.33 | -2.70 – 2.03 | 0.783 | 0.00 | 0.634 |
| 4-Hydroxyproline | -0.09 | -1.17 – 0.99 | 0.866 | 0.00 | 0.658 |
| N,N,N-trimethyl-5-aminovaleric acid | 0.36 | -3.40 – 4.11 | 0.852 | 53.52 | 0.072 |
| Nonanoylcarnitine(AcCa(9:0) | 0.17 | -1.95 – 2.30 | 0.874 | 0.00 | 0.799 |
| Ascorbic acid | -0.15 | -2.71 – 2.42 | 0.912 | 0.00 | 0.690 |

^#^ These metabolites represent the top 50 metabolites ranked by the absolute values of their Spearman correlation coefficients with the T-score in the derivation set.

^*^ Multivariable linear regression analyses were performed separately in each of the internal validation centers, adjusting for age, sex, body mass index, family history of colorectal cancer in first-degree relatives, smoking status, alcohol use, diabetes, hypertension, dietary pattern, and regular use of antibiotics and probiotics. Center-specific effect estimates were pooled using random-effects meta-analysis. Metabolites with a pooled *P* value < 0.050 were considered significantly associated with the T-score. β represents the pooled change in metabolite level per 0.01-unit increase in the T-score. I^2^ represents the percentage of total variation attributable to between-center heterogeneity. *P* heterogeneity represents the *P* value from Cochran’s Q test for between-center heterogeneity. CI, confidence interval.

**Table S13.Summary of model discrimination and calibration performance.**

| **Author, Dataset** | **Spiegelhalter's z statistic** | **Spiegelhalter's z *P* value** |
| --- | --- | --- |
| **Models based on the presence/absence of topological species** |  |  |
| Gupta A, India | 1.22 | 0.225 |
| Wirbel J, Germany | -2.71 | 0.007 |
| Wirbel J, Germany (after recalibration) | 0.01 | 0.993 |
| Feng Q, Austria | 2.89 | 0.004 |
| Feng Q, Austria (after recalibration) | 0.02 | 0.983 |
| Thomas AM, Italy | 0.10 | 0.922 |
| Zeller G, France | 0.90 | 0.367 |
| Yu J, China | -0.67 | 0.500 |
| **Models based on the relative abundance of topological species** |  |  |
| Gupta A, India | 0.93 | 0.351 |
| Wirbel J, Germany | -1.96 | 0.050 |
| Wirbel J, Germany (after recalibration) | -0.00^#^ | 0.999 |
| Feng Q, Austria | 2.33 | 0.020 |
| Feng Q, Austria (after recalibration) | -0.03 | 0.974 |
| Thomas AM, Italy | 0.24 | 0.812 |
| Zeller G, France | 1.35 | 0.178 |
| Yu J, China | -0.11 | 0.910 |

^#^ -0.00 indicates a rounded value of -0.001.

##### Table S14. Conserved association pairs with consistent directions between SparCC networks and CRA-related microbial association patterns.

| **Species** | **Species** | **Odds Ratio (median)^*^** | **Correlation index^#^** |
| --- | --- | --- | --- |
| *Clostridium innocuum* | *Erysipelatoclostridium ramosum* | 10.07 | 0.65 |
| *Enterobacter kobei* | *Enterobacter roggenkampii* | 39.91 | 0.62 |
| *Hungatella hathewayi* | *Clostridium symbiosum* | 9.48 | 0.61 |
| *Blautia hansenii* | *Faecalimonas umbilicata* | 9.93 | 0.61 |
| *Hungatella hathewayi* | *Clostridium innocuum* | 7.04 | 0.56 |
| *Blautia producta* | *Clostridium innocuum* | 12.38 | 0.55 |
| *Blautia caecimuris* | *Erysipelatoclostridium ramosum* | 8.29 | 0.54 |
| *Blautia producta* | *Erysipelatoclostridium ramosum* | 8.50 | 0.54 |
| *Blautia caecimuris* | *Blautia hansenii* | 7.02 | 0.53 |
| *Anaerostipes caccae* | *Clostridium innocuum* | 8.57 | 0.53 |
| *Blautia caecimuris* | *Faecalimonas umbilicata* | 5.28 | 0.52 |
| *Anaerostipes caccae* | *Erysipelatoclostridium ramosum* | 12.49 | 0.52 |
| *Hungatella hathewayi* | *Erysipelatoclostridium ramosum* | 5.77 | 0.52 |
| *Tyzzerella nexilis* | *Erysipelatoclostridium ramosum* | 5.28 | 0.52 |
| *Clostridium symbiosum* | *Clostridium innocuum* | 4.67 | 0.52 |
| *Enterobacter roggenkampii* | *Klebsiella variicola* | 21.00 | 0.51 |
| *Tyzzerella nexilis* | *Clostridium innocuum* | 5.85 | 0.51 |
| *Faecalimonas umbilicata* | *Tyzzerella nexilis* | 5.47 | 0.51 |
| *Anaerostipes caccae* | *Blautia producta* | 6.48 | 0.50 |
| *Faecalimonas umbilicata* | *Erysipelatoclostridium ramosum* | 4.63 | 0.49 |
| *Blautia hansenii* | *Erysipelatoclostridium ramosum* | 5.49 | 0.49 |
| *Clostridium symbiosum* | *Erysipelatoclostridium ramosum* | 4.59 | 0.49 |
| *Blautia caecimuris* | *Tyzzerella nexilis* | 6.72 | 0.48 |
| *Blautia caecimuris* | *Blautia producta* | 9.39 | 0.48 |
| *Faecalimonas umbilicata* | *Clostridium innocuum* | 4.57 | 0.48 |
| *Blautia caecimuris* | *Clostridium innocuum* | 5.52 | 0.48 |
| *Blautia hansenii* | *Clostridium innocuum* | 5.01 | 0.47 |
| *Enterobacter kobei* | *Klebsiella variicola* | 19.96 | 0.46 |
| *Clostridiaceae bacterium Marseille Q4143* | *Lachnospiraceae bacterium AM48 27BH* | 17.23 | 0.46 |
| *Blautia producta* | *Clostridium symbiosum* | 10.67 | 0.46 |
| *Lachnospiraceae bacterium AM48 27BH* | *GGB9614 SGB15049* | 16.75 | 0.45 |
| *Blautia hansenii* | *Tyzzerella nexilis* | 4.20 | 0.45 |
| *Clostridiaceae bacterium Marseille Q4143* | *GGB9616 SGB15052* | 16.48 | 0.43 |
| *Clostridiaceae bacterium Marseille Q4143* | *GGB9614 SGB15049* | 16.63 | 0.43 |
| *Enterobacter hormaechei* | *Enterobacter roggenkampii* | 9.37 | 0.43 |
| *Enterobacter hormaechei* | *Enterobacter kobei* | 8.87 | 0.43 |
| *Oscillibacter sp ER4* | *Oscillospiraceae bacterium Marseille Q3528* | 15.28 | 0.42 |
| *Clostridiaceae bacterium Marseille Q4143* | *Oscillospiraceae bacterium Marseille Q3528* | 17.65 | 0.42 |
| *Anaerostipes caccae* | *Blautia caecimuris* | 6.06 | 0.42 |
| *Clostridiaceae bacterium Marseille Q4143* | *Oscillibacter sp ER4* | 11.30 | 0.41 |
| *Lachnospiraceae bacterium CLA AA H244* | *GGB9614 SGB15049* | 11.60 | 0.41 |
| *GGB9342 SGB14306* | *GGB9602 SGB15031* | 21.12 | 0.41 |
| *Clostridiaceae bacterium Marseille Q4143* | *Clostridiaceae bacterium Marseille Q4145* | 19.81 | 0.40 |
| *Lachnospiraceae bacterium AM48 27BH* | *Oscillibacter sp ER4* | 8.04 | 0.40 |
| *Clostridiaceae bacterium Marseille Q4143* | *Clostridium sp AF32 12BH* | 17.88 | 0.40 |
| *Lachnospiraceae bacterium CLA AA H244* | *Oscillibacter sp ER4* | 9.65 | 0.39 |
| *Lachnospiraceae bacterium AM48 27BH* | *Lachnospiraceae bacterium CLA AA H244* | 10.90 | 0.39 |
| *Enterobacter hormaechei* | *Klebsiella variicola* | 11.03 | 0.39 |
| *Hungatella hathewayi* | *Tyzzerella nexilis* | 4.39 | 0.39 |
| *Lachnospiraceae bacterium CLA AA H244* | *Faecalibacterium SGB15346* | 10.03 | 0.39 |
| *GGB9730 SGB15291* | *Oscillibacter sp ER4* | 9.71 | 0.38 |
| *Hungatella hathewayi* | *Blautia caecimuris* | 5.34 | 0.38 |
| *GGB9614 SGB15049* | *Oscillibacter sp ER4* | 9.90 | 0.38 |
| *Clostridiaceae unclassified SGB4771* | *Oscillospiraceae bacterium Marseille Q3528* | 16.71 | 0.38 |
| *Coprococcus catus* | *Lachnospiraceae bacterium AM48 27BH* | 9.26 | 0.38 |
| *Clostridiaceae bacterium Marseille Q4143* | *Clostridium sp AF12 28* | 10.84 | 0.38 |
| *Oscillibacter sp ER4* | *Oscillospiraceae bacterium* | 13.63 | 0.38 |
| *GGB9593 SGB15015* | *GGB9602 SGB15031* | 17.86 | 0.37 |
| *Faecalibacterium SGB15346* | *GGB9614 SGB15049* | 8.46 | 0.37 |
| *Faecalibacterium SGB15346* | *Oscillibacter sp ER4* | 10.61 | 0.37 |
| *Faecalicatena fissicatena* | *Lachnospiraceae bacterium CLA AA H244* | 9.29 | 0.37 |
| *GGB9758 SGB15368* | *Oscillibacter sp ER4* | 17.49 | 0.37 |
| *GGB9602 SGB15031* | *Oscillospiraceae bacterium* | 15.81 | 0.36 |
| *Clostridiaceae bacterium Marseille Q4143* | *Coprococcus catus* | 10.65 | 0.36 |
| *Clostridiaceae bacterium Marseille Q4143* | *GGB9730 SGB15291* | 10.69 | 0.36 |
| *Coprococcus catus* | *Oscillibacter sp ER4* | 8.90 | 0.36 |
| *GGB34228 SGB72916* | *Oscillospiraceae bacterium* | 14.85 | 0.36 |
| *Faecalicatena fissicatena* | *Lachnospiraceae bacterium AM48 27BH* | 6.42 | 0.36 |
| *GGB9616 SGB15052* | *Oscillospiraceae bacterium Marseille Q3528* | 10.75 | 0.36 |
| *GGB9616 SGB15052* | *GGB9730 SGB15291* | 15.74 | 0.36 |
| *Lachnospiraceae bacterium AM48 27BH* | *Oscillospiraceae bacterium Marseille Q3528* | 10.61 | 0.36 |
| *Clostridiaceae unclassified SGB4771* | *GGB9616 SGB15052* | 12.68 | 0.36 |
| *Clostridium sp AF32 12BH* | *Lachnospiraceae bacterium AM48 27BH* | 13.21 | 0.35 |
| *Clostridiaceae unclassified SGB4771* | *Anaerosacchariphilus sp NSJ 68* | 14.05 | 0.35 |
| *GGB9730 SGB15291* | *Oscillospiraceae bacterium Marseille Q3528* | 12.64 | 0.35 |
| *GGB9758 SGB15368* | *GGB9760 SGB15373* | 8.07 | 0.35 |
| *GGB9614 SGB15049* | *Oscillospiraceae bacterium Marseille Q3528* | 14.90 | 0.35 |
| *Clostridiaceae bacterium Marseille Q4145* | *GGB9616 SGB15052* | 10.05 | 0.35 |
| *Lachnospiraceae bacterium CLA AA H244* | *GGB9730 SGB15291* | 12.69 | 0.35 |
| *Clostridiaceae bacterium Marseille Q4143* | *Lachnospiraceae bacterium CLA AA H244* | 17.49 | 0.35 |
| *GGB9616 SGB15052* | *Oscillibacter sp ER4* | 11.24 | 0.35 |
| *Clostridiaceae bacterium Marseille Q4145* | *Oscillospiraceae bacterium Marseille Q3528* | 16.80 | 0.34 |
| *Coprococcus catus* | *GGB9730 SGB15291* | 10.55 | 0.34 |
| *Lachnospiraceae bacterium CLA AA H244* | *Oscillospiraceae bacterium Marseille Q3528* | 13.31 | 0.34 |
| *Clostridium sp AF12 28* | *GGB9616 SGB15052* | 8.69 | 0.34 |
| *Clostridium sp AF32 12BH* | *GGB9616 SGB15052* | 10.99 | 0.34 |
| *Lachnospiraceae bacterium AM48 27BH* | *Faecalibacterium SGB15346* | 9.68 | 0.34 |
| *Faecalibacterium SGB15346* | *GGB9730 SGB15291* | 15.25 | 0.34 |
| *GGB9342 SGB14306* | *GGB13493 SGB15238* | 21.31 | 0.34 |
| *Lachnospiraceae bacterium AM48 27BH* | *GGB9730 SGB15291* | 6.84 | 0.34 |
| *Clostridiaceae bacterium Marseille Q4143* | *Clostridiaceae unclassified SGB4771* | 12.27 | 0.34 |
| *Clostridiaceae bacterium Marseille Q4145* | *Clostridiaceae unclassified SGB4771* | 11.59 | 0.34 |
| *Oscillospiraceae bacterium* | *Oscillospiraceae bacterium Marseille Q3528* | 9.86 | 0.34 |
| *Anaerosacchariphilus sp NSJ 68* | *Oscillospiraceae bacterium Marseille Q3528* | 20.08 | 0.34 |
| *GGB9760 SGB15373* | *Oscillibacter sp ER4* | 14.98 | 0.34 |
| *Clostridium sp AF12 28* | *Lachnospiraceae bacterium AM48 27BH* | 13.78 | 0.34 |
| *Anaerosacchariphilus sp NSJ 68* | *GGB9616 SGB15052* | 13.19 | 0.33 |
| *Lachnospiraceae bacterium AM48 27BH* | *GGB9616 SGB15052* | 19.90 | 0.33 |
| *GGB9614 SGB15049* | *GGB9616 SGB15052* | 12.06 | 0.33 |
| *Clostridium sp AF12 28* | *Clostridium sp AF32 12BH* | 10.06 | 0.33 |
| *Coprococcus catus* | *Faecalicatena fissicatena* | 6.98 | 0.33 |
| *Clostridiaceae unclassified SGB4771* | *Clostridium sp AF32 12BH* | 11.29 | 0.33 |
| *Clostridiaceae unclassified SGB4771* | *Clostridium sp AF12 28* | 10.08 | 0.33 |
| *Clostridiaceae bacterium Marseille Q4143* | *Faecalicatena fissicatena* | 8.85 | 0.33 |
| *Coprococcus catus* | *Dorea longicatena* | 21.34 | 0.32 |
| *GGB9342 SGB14306* | *GGB9758 SGB15368* | 13.83 | 0.32 |
| *Coprococcus catus* | *Oscillospiraceae bacterium Marseille Q3528* | 14.20 | 0.32 |
| *Faecalicatena fissicatena* | *Faecalibacterium SGB15346* | 7.47 | 0.32 |
| *Clostridiaceae unclassified SGB4771* | *GGB9730 SGB15291* | 11.25 | 0.32 |
| *Clostridiaceae unclassified SGB4771* | *Oscillibacter sp ER4* | 14.17 | 0.32 |
| *Clostridiaceae bacterium Marseille Q4145* | *Anaerosacchariphilus sp NSJ 68* | 13.90 | 0.32 |
| *GGB9758 SGB15368* | *GGB9602 SGB15031* | 8.53 | 0.32 |
| *Clostridiaceae bacterium Marseille Q4145* | *Clostridium sp AF12 28* | 9.66 | 0.32 |
| *Coprococcus catus* | *GGB9614 SGB15049* | 9.07 | 0.32 |
| *Coprococcus catus* | *Faecalibacterium SGB15346* | 7.85 | 0.32 |
| *GGB9758 SGB15368* | *Oscillospiraceae bacterium Marseille Q3528* | 9.12 | 0.32 |
| *GGB9614 SGB15049* | *GGB9730 SGB15291* | 9.59 | 0.31 |
| *Faecalicatena fissicatena* | *Oscillibacter sp ER4* | 6.51 | 0.31 |
| *GGB9595 SGB15019* | *GGB9602 SGB15031* | 20.80 | 0.31 |
| *Clostridiaceae bacterium Marseille Q4143* | *Faecalibacterium SGB15346* | 11.53 | 0.31 |
| *Clostridiaceae unclassified SGB4771* | *Lachnospiraceae bacterium AM48 27BH* | 12.25 | 0.31 |
| *GGB58485 SGB80143* | *Anaerosacchariphilus sp NSJ 68* | 15.66 | 0.31 |
| *Clostridium SGB4751* | *Clostridium sp AF32 12BH* | 11.11 | 0.31 |
| *Faecalicatena fissicatena* | *GGB9614 SGB15049* | 6.85 | 0.31 |
| *GGB9760 SGB15373* | *GGB9602 SGB15031* | 15.23 | 0.31 |
| *GGB34228 SGB72916* | *Oscillospiraceae bacterium Marseille Q3528* | 21.88 | 0.31 |
| *Lachnospiraceae bacterium CLA AA H244* | *Lachnospira sp NSJ 43* | 9.81 | 0.31 |
| *Anaerosacchariphilus sp NSJ 68* | *GGB9602 SGB15031* | 8.89 | 0.30 |
| *GGB3828 SGB5197* | *Lachnospiraceae bacterium AM48 27BH* | 6.99 | 0.30 |
| *Blautia hansenii* | *Coprococcus catus* | 0.18 | -0.31 |
| *Coprococcus catus* | *Tyzzerella nexilis* | 0.24 | -0.31 |
| *Clostridiaceae unclassified SGB4771* | *Tyzzerella nexilis* | 0.18 | -0.31 |
| *Dorea longicatena* | *Tyzzerella nexilis* | 0.16 | -0.32 |
| *Oscillospiraceae bacterium* | *Erysipelatoclostridium ramosum* | 0.19 | -0.32 |
| *Clostridium sp AF32 12BH* | *Clostridium innocuum* | 0.15 | -0.32 |
| *Anaerostipes caccae* | *GGB9730 SGB15291* | 0.11 | -0.32 |
| *Clostridiaceae unclassified SGB4771* | *Clostridium innocuum* | 0.15 | -0.32 |
| *GGB9616 SGB15052* | *Erysipelatoclostridium ramosum* | 0.16 | -0.32 |
| *Dorea longicatena* | *Erysipelatoclostridium ramosum* | 0.10 | -0.33 |
| *Tyzzerella nexilis* | *GGB9614 SGB15049* | 0.26 | -0.33 |
| *Blautia producta* | *GGB9730 SGB15291* | 0.09 | -0.33 |
| *Clostridium sp AF12 28* | *Clostridium innocuum* | 0.20 | -0.33 |
| *Clostridium sp AF12 28* | *Erysipelatoclostridium ramosum* | 0.18 | -0.33 |
| *Hungatella hathewayi* | *GGB9730 SGB15291* | 0.28 | -0.34 |
| *Blautia hansenii* | *GGB9730 SGB15291* | 0.14 | -0.34 |
| *Blautia caecimuris* | *Faecalicatena fissicatena* | 0.16 | -0.34 |
| *Coprococcus catus* | *Faecalimonas umbilicata* | 0.17 | -0.34 |
| *Hungatella hathewayi* | *Coprococcus catus* | 0.25 | -0.34 |
| *Faecalicatena fissicatena* | *Clostridium innocuum* | 0.26 | -0.34 |
| *Blautia caecimuris* | *Coprococcus catus* | 0.18 | -0.34 |
| *Blautia hansenii* | *Oscillospiraceae bacterium Marseille Q3528* | 0.13 | -0.34 |
| *Anaerostipes caccae* | *GGB9614 SGB15049* | 0.15 | -0.35 |
| *Tyzzerella nexilis* | *Faecalibacterium SGB15346* | 0.18 | -0.35 |
| *Faecalicatena fissicatena* | *Tyzzerella nexilis* | 0.19 | -0.35 |
| *Clostridiaceae bacterium Marseille Q4143* | *Blautia producta* | 0.11 | -0.35 |
| *Clostridiaceae bacterium Marseille Q4143* | *Tyzzerella nexilis* | 0.23 | -0.35 |
| *GGB9758 SGB15368* | *Erysipelatoclostridium ramosum* | 0.19 | -0.35 |
| *Blautia producta* | *Oscillospiraceae bacterium Marseille Q3528* | 0.09 | -0.35 |
| *Blautia caecimuris* | *Oscillospiraceae bacterium Marseille Q3528* | 0.14 | -0.36 |
| *Tyzzerella nexilis* | *Oscillospiraceae bacterium Marseille Q3528* | 0.22 | -0.36 |
| *Lachnospiraceae bacterium CLA AA H244* | *Tyzzerella nexilis* | 0.24 | -0.36 |
| *Clostridiaceae bacterium Marseille Q4143* | *Blautia hansenii* | 0.13 | -0.36 |
| *GGB9616 SGB15052* | *Clostridium innocuum* | 0.13 | -0.36 |
| *Blautia producta* | *Lachnospiraceae bacterium CLA AA H244* | 0.18 | -0.37 |
| *Clostridiaceae bacterium Marseille Q4143* | *Faecalimonas umbilicata* | 0.11 | -0.37 |
| *Faecalimonas umbilicata* | *GGB9730 SGB15291* | 0.09 | -0.37 |
| *Tyzzerella nexilis* | *GGB9730 SGB15291* | 0.22 | -0.37 |
| *Lachnospiraceae bacterium AM48 27BH* | *Tyzzerella nexilis* | 0.25 | -0.37 |
| *Faecalicatena fissicatena* | *Erysipelatoclostridium ramosum* | 0.17 | -0.37 |
| *Blautia caecimuris* | *GGB9614 SGB15049* | 0.17 | -0.38 |
| *Anaerostipes caccae* | *Lachnospiraceae bacterium AM48 27BH* | 0.16 | -0.38 |
| *Blautia hansenii* | *Lachnospiraceae bacterium CLA AA H244* | 0.17 | -0.38 |
| *Oscillospiraceae bacterium Marseille Q3528* | *Erysipelatoclostridium ramosum* | 0.17 | -0.38 |
| *Faecalimonas umbilicata* | *Oscillospiraceae bacterium Marseille Q3528* | 0.12 | -0.38 |
| *GGB9730 SGB15291* | *Clostridium innocuum* | 0.17 | -0.39 |
| *Blautia producta* | *Coprococcus catus* | 0.17 | -0.39 |
| *Blautia hansenii* | *Lachnospiraceae bacterium AM48 27BH* | 0.16 | -0.39 |
| *GGB9730 SGB15291* | *Erysipelatoclostridium ramosum* | 0.12 | -0.39 |
| *Blautia caecimuris* | *GGB9730 SGB15291* | 0.07 | -0.39 |
| *Clostridiaceae bacterium Marseille Q4143* | *Blautia caecimuris* | 0.13 | -0.39 |
| *Oscillospiraceae bacterium Marseille Q3528* | *Clostridium innocuum* | 0.18 | -0.40 |
| *Anaerostipes caccae* | *Lachnospiraceae bacterium CLA AA H244* | 0.15 | -0.40 |
| *Blautia caecimuris* | *Lachnospiraceae bacterium AM48 27BH* | 0.16 | -0.40 |
| *Blautia hansenii* | *Faecalibacterium SGB15346* | 0.14 | -0.40 |
| *Blautia hansenii* | *GGB9614 SGB15049* | 0.13 | -0.40 |
| *Clostridiaceae bacterium Marseille Q4143* | *Erysipelatoclostridium ramosum* | 0.18 | -0.40 |
| *Faecalimonas umbilicata* | *Faecalibacterium SGB15346* | 0.16 | -0.41 |
| *Faecalibacterium SGB15346* | *Clostridium innocuum* | 0.16 | -0.41 |
| *Faecalimonas umbilicata* | *GGB9614 SGB15049* | 0.13 | -0.41 |
| *GGB9614 SGB15049* | *Erysipelatoclostridium ramosum* | 0.16 | -0.41 |
| *Clostridiaceae bacterium Marseille Q4143* | *Clostridium innocuum* | 0.18 | -0.42 |
| *Blautia caecimuris* | *Lachnospiraceae bacterium CLA AA H244* | 0.16 | -0.42 |
| *Coprococcus catus* | *Clostridium innocuum* | 0.14 | -0.42 |
| *Lachnospiraceae bacterium CLA AA H244* | *Clostridium innocuum* | 0.17 | -0.42 |
| *Lachnospiraceae bacterium AM48 27BH* | *Erysipelatoclostridium ramosum* | 0.17 | -0.42 |
| *Lachnospiraceae bacterium CLA AA H244* | *Erysipelatoclostridium ramosum* | 0.14 | -0.42 |
| *Blautia caecimuris* | *Faecalibacterium SGB15346* | 0.14 | -0.43 |
| *Faecalibacterium SGB15346* | *Erysipelatoclostridium ramosum* | 0.13 | -0.43 |
| *Lachnospiraceae bacterium AM48 27BH* | *Clostridium innocuum* | 0.18 | -0.43 |
| *GGB9614 SGB15049* | *Clostridium innocuum* | 0.19 | -0.43 |
| *Coprococcus catus* | *Erysipelatoclostridium ramosum* | 0.11 | -0.43 |
| *Blautia caecimuris* | *Oscillibacter sp ER4* | 0.21 | -0.43 |
| *Faecalimonas umbilicata* | *Lachnospiraceae bacterium CLA AA H244* | 0.14 | -0.44 |
| *Blautia hansenii* | *Oscillibacter sp ER4* | 0.18 | -0.44 |
| *Faecalimonas umbilicata* | *Lachnospiraceae bacterium AM48 27BH* | 0.11 | -0.44 |
| *Oscillibacter sp ER4* | *Clostridium innocuum* | 0.26 | -0.45 |
| *Faecalimonas umbilicata* | *Oscillibacter sp ER4* | 0.17 | -0.46 |
| *Oscillibacter sp ER4* | *Erysipelatoclostridium ramosum* | 0.15 | -0.47 |

* Median odds ratio from Fisher's exact test with bootstrap resampling for microbial association patterns.

\# Correlation index was derived from SparCC network.

##### Table S15. Conserved microbial association pairs overlapping between CRA-related microbial association patterns and SPIEC-EASI networks.

| **Species** | **Species** | **Direction** |
| --- | --- | --- |
| *Enterobacter kobei* | *Enterobacter roggenkampii* | Positive |
| *Blautia hansenii* | *Faecalimonas umbilicata* | Positive |
| *Clostridium innocuum* | *Erysipelatoclostridium ramosum* | Positive |
| *Enterobacter roggenkampii* | *Klebsiella variicola* | Positive |
| *Hungatella hathewayi* | *Clostridium symbiosum* | Positive |
| *Anaerostipes caccae* | *Blautia producta* | Positive |
| *Blautia producta* | *Clostridium innocuum* | Positive |
| *Clostridiaceae bacterium Marseille Q4143* | *GGB9616 SGB15052* | Positive |
| *Clostridiaceae bacterium Marseille Q4143* | *Clostridiaceae bacterium Marseille Q4145* | Positive |
| *Clostridiaceae bacterium Marseille Q4143* | *Clostridium sp AF32 12BH* | Positive |
| *Anaerostipes caccae* | *Clostridium innocuum* | Positive |
| *Blautia caecimuris* | *Erysipelatoclostridium ramosum* | Positive |
| *Blautia caecimuris* | *Blautia hansenii* | Positive |
| *Enterobacter hormaechei* | *Enterobacter kobei* | Positive |
| *Anaerostipes caccae* | *Erysipelatoclostridium ramosum* | Positive |
| *Clostridiaceae bacterium Marseille Q4143* | *Lachnospiraceae bacterium AM48 27BH* | Positive |
| *GGB9593 SGB15015* | *GGB9602 SGB15031* | Positive |
| *Faecalimonas umbilicata* | *Tyzzerella nexilis* | Positive |
| *Enterobacter hormaechei* | *Enterobacter roggenkampii* | Positive |
| *Hungatella hathewayi* | *Clostridium innocuum* | Positive |
| *Faecalimonas umbilicata* | *Lachnospiraceae bacterium AM48 27BH* | Negative |
| *Lachnospiraceae bacterium AM48 27BH* | *GGB9614 SGB15049* | Positive |
| *Coprococcus catus* | *Erysipelatoclostridium ramosum* | Negative |
| *Clostridiaceae unclassified SGB4771* | *Oscillospiraceae bacterium Marseille Q3528* | Positive |
| *GGB9758 SGB15368* | *GGB9760 SGB15373* | Positive |
| *Blautia producta* | *Erysipelatoclostridium ramosum* | Positive |
| *Clostridiaceae bacterium Marseille Q4143* | *GGB9614 SGB15049* | Positive |
| *Blautia caecimuris* | *Faecalimonas umbilicata* | Positive |
| *Blautia caecimuris* | *Blautia producta* | Positive |
| *GGB9342 SGB14306* | *GGB9602 SGB15031* | Positive |
| *Clostridiaceae bacterium Marseille Q4143* | *Clostridium sp AF12 28* | Positive |
| *Clostridiaceae bacterium Marseille Q4143* | *Oscillospiraceae bacterium Marseille Q3528* | Positive |
| *Lachnospiraceae bacterium CLA AA H244* | *Lachnospira sp NSJ 43* | Positive |
| *Clostridium SGB4751* | *Clostridium sp AF32 12BH* | Positive |
| *Clostridiaceae unclassified SGB4771* | *Anaerosacchariphilus sp NSJ 68* | Positive |
| *Tyzzerella nexilis* | *Erysipelatoclostridium ramosum* | Positive |
| *Blautia caecimuris* | *Faecalibacterium SGB15346* | Negative |
| *GGB58485 SGB80143* | *Anaerosacchariphilus sp NSJ 68* | Positive |
| *Enterobacter kobei* | *Klebsiella variicola* | Positive |
| *Faecalicatena fissicatena* | *Erysipelatoclostridium ramosum* | Negative |
| *Tyzzerella nexilis* | *Clostridium innocuum* | Positive |
| *Faecalicatena fissicatena* | *Lachnospiraceae bacterium CLA AA H244* | Positive |
| *Faecalicatena fissicatena* | *Lachnospiraceae bacterium AM48 27BH* | Positive |
| *GGB51884 SGB49168* | *GGB9759 SGB15370* | Positive |
| *GGB9760 SGB15373* | *Oscillibacter sp ER4* | Positive |
| *GGB34228 SGB72916* | *Oscillospiraceae bacterium* | Positive |
| *Faecalimonas umbilicata* | *Lachnospiraceae bacterium CLA AA H244* | Negative |
| *Blautia producta* | *Clostridium symbiosum* | Positive |
| *Clostridiaceae bacterium Marseille Q4143* | *Clostridium SGB4751* | Positive |
| *GGB9614 SGB15049* | *Clostridium innocuum* | Negative |
| *Faecalibacterium SGB15346* | *Erysipelatoclostridium ramosum* | Negative |
| *Blautia caecimuris* | *Tyzzerella nexilis* | Positive |
| *GGB9595 SGB15019* | *GGB9602 SGB15031* | Positive |
| *Faecalicatena fissicatena* | *Roseburia lenta* | Positive |
| *Clostridium sp AF32 12BH* | *Lachnospiraceae bacterium AM48 27BH* | Positive |
| *Lachnospiraceae bacterium CLA AA H244* | *GGB9614 SGB15049* | Positive |
| *GGB3828 SGB5197* | *Lachnospiraceae bacterium AM48 27BH* | Positive |
| *Oscillibacter sp ER4* | *Oscillospiraceae bacterium* | Positive |
| *GGB9616 SGB15052* | *GGB9730 SGB15291* | Positive |
| *GGB9602 SGB15031* | *GGB9634 SGB15093* | Positive |
| *Clostridium sp AF32 12BH* | *Blautia stercoris* | Positive |
| *Enterobacter hormaechei* | *Klebsiella variicola* | Positive |
| *Clostridiaceae unclassified SGB4771* | *Clostridium sp AF32 12BH* | Positive |
| *GGB9758 SGB15368* | *Oscillibacter sp ER4* | Positive |
| *GGB9760 SGB15373* | *GGB9602 SGB15031* | Positive |
| *GGB58485 SGB80143* | *GGB34228 SGB72916* | Positive |
| *Blautia producta* | *Blautia stercoris* | Negative |
| *Faecalicatena fissicatena* | *Tyzzerella nexilis* | Negative |
| *Coprococcus catus* | *Clostridium innocuum* | Negative |
| *Anaerosacchariphilus sp NSJ 68* | *GGB9616 SGB15052* | Positive |
| *Oscillibacter sp ER4* | *Oscillospiraceae bacterium Marseille Q3528* | Positive |
| *Anaerosacchariphilus sp NSJ 68* | *Oscillospiraceae bacterium Marseille Q3528* | Positive |
| *Clostridium sp AF32 12BH* | *GGB3828 SGB5197* | Positive |
| *Blautia caecimuris* | *GGB9730 SGB15291* | Negative |
| *Clostridium sp AF12 28* | *GGB9616 SGB15052* | Positive |
| *Blautia producta* | *Coprococcus catus* | Negative |
| *Blautia hansenii* | *Erysipelatoclostridium ramosum* | Positive |
| *Blautia stercoris* | *Roseburia lenta* | Positive |
| *Clostridiaceae bacterium Marseille Q4145* | *Anaerosacchariphilus sp NSJ 68* | Positive |
| *Clostridium sp AF32 12BH* | *GGB9616 SGB15052* | Positive |
| *Blautia caecimuris* | *Lachnospiraceae bacterium CLA AA H244* | Negative |
| *Clostridium sp AF32 12BH* | *Roseburia lenta* | Positive |
| *Clostridiaceae bacterium Marseille Q4145* | *Oscillospiraceae bacterium Marseille Q3528* | Positive |
| *Clostridium sp AF12 28* | *Clostridium sp AF32 12BH* | Positive |
| *Anaerostipes caccae* | *Lachnospiraceae bacterium CLA AA H244* | Negative |
| *GGB9342 SGB14306* | *GGB13493 SGB15238* | Positive |
| *Clostridiaceae unclassified SGB4771* | *GGB9616 SGB15052* | Positive |
| *Blautia hansenii* | *Faecalibacterium SGB15346* | Negative |
| *GGB58485 SGB80143* | *Clostridiaceae bacterium Marseille Q4145* | Positive |
| *Oscillibacter sp ER4* | *Erysipelatoclostridium ramosum* | Negative |
| *Coprococcus catus* | *Lachnospiraceae bacterium AM48 27BH* | Positive |
| *GGB9730 SGB15291* | *Oscillibacter sp ER4* | Positive |
| *Clostridiaceae unclassified SGB4771* | *Clostridium sp AF12 28* | Positive |
| *GGB51884 SGB49168* | *GGB9593 SGB15015* | Positive |
| *GGB51884 SGB49168* | *GGB9602 SGB15031* | Positive |
| *Clostridiaceae bacterium Marseille Q4143* | *Oscillibacter sp ER4* | Positive |
| *Faecalimonas umbilicata* | *Faecalibacterium SGB15346* | Negative |
| *Faecalimonas umbilicata* | *Oscillibacter sp ER4* | Negative |
| *GGB9595 SGB15019* | *GGB9634 SGB15093* | Positive |
| *Clostridiaceae bacterium Marseille Q4145* | *Clostridiaceae unclassified SGB4771* | Positive |
| *Hungatella hathewayi* | *Erysipelatoclostridium ramosum* | Positive |
| *Blautia hansenii* | *GGB9614 SGB15049* | Negative |
| *Hungatella hathewayi* | *GGB3828 SGB5197* | Negative |
| *GGB3828 SGB5197* | *GGB9614 SGB15049* | Positive |
| *Anaerosacchariphilus sp NSJ 68* | *GGB9602 SGB15031* | Positive |
| *Clostridium sp AF12 28* | *Lachnospiraceae bacterium AM48 27BH* | Positive |
| *Clostridium sp AF12 28* | *Erysipelatoclostridium ramosum* | Negative |
| *Faecalimonas umbilicata* | *GGB9614 SGB15049* | Negative |
| *Clostridiaceae bacterium Marseille Q4145* | *GGB9616 SGB15052* | Positive |
| *GGB51884 SGB49168* | *GGB34228 SGB72916* | Positive |
| *Coprococcus catus* | *Faecalicatena fissicatena* | Positive |
| *Faecalibacterium SGB15346* | *Clostridium innocuum* | Negative |
| *Anaerostipes caccae* | *Blautia hansenii* | Positive |
| *GGB9342 SGB14306* | *GGB9758 SGB15368* | Positive |
| *GGB9602 SGB15031* | *Oscillospiraceae bacterium* | Positive |
| *GGB58485 SGB80143* | *Oscillospiraceae bacterium Marseille Q3528* | Positive |
| *Clostridiaceae bacterium Marseille Q4143* | *Blautia caecimuris* | Negative |
| *Dorea longicatena* | *Tyzzerella nexilis* | Negative |
| *Clostridium symbiosum* | *Clostridium innocuum* | Positive |
| *GGB9616 SGB15052* | *Oscillospiraceae bacterium Marseille Q3528* | Positive |
| *Lachnospiraceae bacterium AM48 27BH* | *Clostridium innocuum* | Negative |
| *Clostridiaceae bacterium Marseille Q4143* | *Roseburia lenta* | Positive |
| *Lachnospiraceae bacterium AM48 27BH* | *Erysipelatoclostridium ramosum* | Negative |
| *Blautia stercoris* | *Lachnospiraceae bacterium AM48 27BH* | Positive |
| *Blautia hansenii* | *Blautia stercoris* | Negative |
| *Roseburia lenta* | *GGB9614 SGB15049* | Positive |
| *Clostridiaceae bacterium Marseille Q4143* | *Coprococcus catus* | Positive |
| *Clostridiaceae bacterium Marseille Q4143* | *Clostridium innocuum* | Negative |
| *Lachnospiraceae bacterium CLA AA H244* | *Clostridium innocuum* | Negative |
| *Clostridiaceae bacterium Marseille Q4145* | *Clostridium sp AF12 28* | Positive |
| *Blautia stercoris* | *Coprococcus catus* | Positive |
| *Blautia hansenii* | *Clostridium innocuum* | Positive |
| *GGB9302 SGB14263* | *GGB9602 SGB15031* | Positive |
| *Hungatella hathewayi* | *Faecalicatena fissicatena* | Negative |
| *Clostridium sp AF12 28* | *Clostridium innocuum* | Negative |
| *GGB9758 SGB15368* | *GGB9602 SGB15031* | Positive |
| *GGB9758 SGB15368* | *Erysipelatoclostridium ramosum* | Negative |
| *Faecalicatena fissicatena* | *GGB3828 SGB5197* | Positive |
| *Coprococcus catus* | *Dorea longicatena* | Positive |
| *GGB58485 SGB80143* | *GGB9593 SGB15015* | Positive |
| *GGB9614 SGB15049* | *Erysipelatoclostridium ramosum* | Negative |
| *Blautia caecimuris* | *Faecalicatena fissicatena* | Negative |
| *GGB9616 SGB15052* | *Clostridium innocuum* | Negative |
| *GGB9730 SGB15291* | *Erysipelatoclostridium ramosum* | Negative |
| *Lachnospiraceae bacterium AM48 27BH* | *Oscillibacter sp ER4* | Positive |
| *Blautia stercoris* | *Dorea longicatena* | Positive |
| *Clostridiaceae bacterium Marseille Q4143* | *GGB9730 SGB15291* | Positive |
| *Faecalibacterium SGB15346* | *GGB9614 SGB15049* | Positive |
| *GGB9758 SGB15368* | *GGB9634 SGB15093* | Positive |
| *GGB34228 SGB72916* | *GGB9595 SGB15019* | Positive |
| *Blautia hansenii* | *Oscillibacter sp ER4* | Negative |
| *Lachnospiraceae bacterium CLA AA H244* | *Faecalibacterium SGB15346* | Positive |
| *Clostridiaceae bacterium Marseille Q4143* | *Faecalicatena fissicatena* | Positive |
| *GGB9760 SGB15373* | *Oscillospiraceae bacterium Marseille Q3528* | Positive |
| *GGB9760 SGB15373* | *Clostridium innocuum* | Negative |
| *GGB58485 SGB80143* | *GGB9602 SGB15031* | Positive |
| *Coprococcus catus* | *GGB9730 SGB15291* | Positive |
| *Clostridiaceae unclassified SGB4771* | *GGB9730 SGB15291* | Positive |
| *Oscillospiraceae bacterium Marseille Q3528* | *Clostridium innocuum* | Negative |
| *Faecalimonas umbilicata* | *Oscillospiraceae bacterium Marseille Q3528* | Negative |
| *GGB9730 SGB15291* | *Oscillospiraceae bacterium Marseille Q3528* | Positive |
| *GGB9730 SGB15291* | *Clostridium innocuum* | Negative |
| *Clostridium SGB4751* | *Anaerosacchariphilus sp NSJ 68* | Positive |
| *Blautia caecimuris* | *Oscillibacter sp ER4* | Negative |
| *Tyzzerella nexilis* | *GGB9730 SGB15291* | Negative |
| *Clostridiaceae bacterium Marseille Q4145* | *Clostridium SGB4751* | Positive |
| *GGB9614 SGB15049* | *Oscillospiraceae bacterium Marseille Q3528* | Positive |
